# Atomically Precise Engineering of Synergistic Binding Sites in a Zirconium Metal–Organic Framework for the Capture of Perfluorooctanoic Acid

**DOI:** 10.1021/jacs.5c23392

**Published:** 2026-04-10

**Authors:** Sergio Marugán-Benito, Michalis Vlachos, Lutz Ahrens, Miguel Roselló-González, Carlo Marini, Jordi Prat Albert, Andreas Mavrandonakis, Edward Loukopoulos, Ana E. Platero-Prats

**Affiliations:** † Departamento de Química Inorgánica, 83076 Universidad Autónoma de Madrid, Madrid 28049, Spain; ‡ 16379 Material Science Institute of Madrid (ICMM-CSIC), Madrid 28049, Spain; § Department of Aquatic Sciences and Assessment, 8095 Swedish University of Agricultural Sciences (SLU), Uppsala SE-75007, Sweden; ∥ 430128 ALBA Synchrotron Light Source, Cerdanyola del Vallès, Barcelona 08290, Spain; ⊥ Condensed Matter Physics Center (IFIMAC), Universidad Autónoma de Madrid, Madrid 28049, Spain; # Instituto de Catálisis y Petroleoquímica (ICP-CSIC), Madrid 28049, Spain

## Abstract

The persistent contamination of water sources by perfluorooctanoic acid (PFOA) poses a major environmental and public health challenge. PFOA is a representative member of per- and polyfluoroalkyl substances (PFAS), a class of compounds characterized by high chemical stability, bioaccumulation potential, and toxicity. Conventional water treatment processes are not fully effective in removing PFOA, underscoring the urgent need for advanced remediation strategies. Here, we report the development of Fe-MOF-808, a novel porous material obtained by incorporating binuclear iron species into the Zr_6_O_8_ nodes of the MOF-808 framework. Comprehensive structural characterization was performed, including *ex*/*in situ* synchrotron-based techniques combined with computational modeling. The results confirm successful iron integration without compromising the structural integrity and accessibility of the porous network. Moreover, the presence of multiple, spatially accessible binding sites enables Fe-MOF-808 to capture PFAS through a combination of electrostatic, hydrophobic and coordinative interactions. This resulted in high removal efficiencies across various water matrices and for a wide range of PFAS pollutants and concentrations. Fe-MOF-808 notably achieves complete PFOA removal within minutes and demonstrates excellent recyclability over multiple adsorption cycles. The material also reaches experimental uptake and a maximum Langmuir adsorption capacity of 2081 and 3120 mg PFOA g^–1^, respectively, vastly outperforming the pristine MOF-808 and other state-of-the-art MOF materials. Overall, mechanistic insights gained from this study highlight the critical role of designing specific chemical environments within MOFs to maximize pollutant-sorbent interactions.

## Graphical abstract

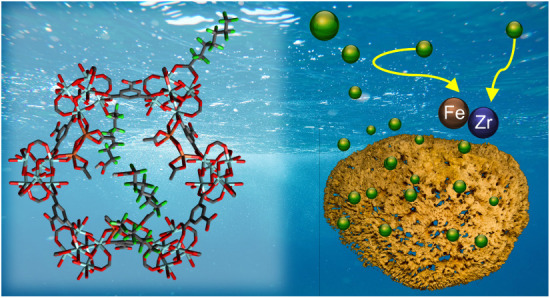

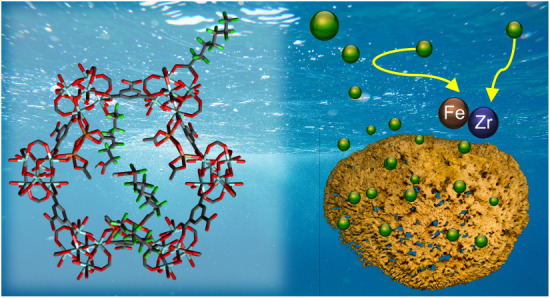

## Introduction

The growing environmental crisis has signaled a global shift to more sustainable practices. Among these, ensuring access to clean water is critical, as it directly impacts agriculture, economy and energy.^1^ However, rising industrial activities over the past decades has brought additional water contamination from man-made pollutants, posing significant environmental and health risks.^2^

One group of particular interest is per- and polyfluoroalkyl substances (PFAS), with perfluorooctanoic acid (PFOA) a key compound of global concern.^3–5^ This chemical shows extreme persistence since its highly stable C–F chain backbone prevents it from fully degrading under conventional wastewater treatments.^6^ Its long biological half-life in humans (from 2 to 14.5 years,^7,8^ depending on community^9^) has led to significant bioaccumulation and toxicity. Exposure to PFOA has been linked to adverse health effects, including cancer, immune and liver system damage, and fetal development issues.^10,11^ In response to these concerns, regulations around the world have set PFOA exposure limits to trace concentrations (ppt or ppb levels).^12,13^ Despite these measures, existing remediation methods (e.g., electrochemical oxidation, thermal treatments, activated carbon, ion exchange resins) are limited by suboptimal efficacy, high energy and cost demands.^14^ Moreover, many PFAS compounds degrade into more stable, water-soluble and mobile species during their life-cycle, with PFOA often serving as a terminal product.^15^ Consequently, PFOA remains widely detected in the environment, typically at low-ppb levels in drinking water and human serum, but potentially reaching up to several ppm in industrial sites and exposed workers.^16,17^ Therefore, more effective strategies are urgently required to fully remediate water sources from this pollutant.

In light of these limitations, porous platforms with higher chemical tunability have attracted increasing interest for PFAS removal via adsorption, thereby bypassing their resistance to other water treatment approaches. Among such materials, metal–organic frameworks (MOFs) have emerged as particularly promising candidates.^18^ MOFs uniquely integrate organic and inorganic building units in their structures, providing unparalleled control at the molecular level.^19,20^ This chemical versatility has enabled development of certain MOFs for water remediation, overcoming typical issues of hydrolytic stability.^21^ However, the specific use of MOFs for PFAS decontamination remains suboptimal; significant challenges arise from the chemical nature (e.g., chain size, functional group) and low concentrations of these pollutants,^14,22^ as well as the presence of interfering agents within complex water matrices. While several pristine MOFs have been employed to remove PFOA, the full potential of these materials beyond their high porosity remains largely untapped.^22^

Recent studies, including our own, have explored design strategies that tailor MOF chemistry to interact specifically with PFOA through electrostatic,^23–25^ hydrophobic,^26–28^ coordinative^23,28–30^ or H-bonding^31^ interactions. The ultimate goal is to engineer multifunctional MOFs with optimized local environments that maximize synergistic pollutant···MOF interactions. This approach would lead to enhanced adsorption capacities with fast kinetics and good recyclability, overcoming common limitations of other state-of-the-art PFAS adsorbents such as activated carbons or ion-exchange resins.^32–34^ Building on these concepts, we reasoned that the incorporation of appropriate secondary metal sites could be an efficient strategy to further tailor the adsorption environment in suitable MOFs. Such a modification can create additional favorable binding sites in close proximity, thereby promoting synergistic interactions with PFOA molecules. To the best of our knowledge, this design approach remains unexplored in MOFs for PFAS remediation. We herein report a novel MOF-based adsorbent featuring binuclear iron species into the Zr_6_O_8_ clusters of the well-known MOF-808 structure.^35^ Advanced characterization, including synchrotron-based techniques and computational modeling, confirms successful Fe incorporation and the preservation of the porous architecture. The resulting Fe-MOF-808 exhibits high stability and enhanced spatial accessibility to multiple binding sites, making it particularly well-suited for PFOA capture. The cooperative effect of multiple interactions leads to significant improvement in adsorption performance compared to the pristine MOF-808 material. Fe-MOF-808 achieves rapid and complete PFOA removal despite the presence of various interfering agents, maintaining performance over multiple cycles. Additional tests for a range of PFAS pollutants and concentrations also reveal initial practical potential for this material. Its experimental and Langmuir-fitted PFOA adsorption capacities surpass all previously reported MOFs and position it among leading state-of-the-art porous materials. Comprehensive *ex situ* and *in situ* synchrotron studies combined with theoretical calculations provide unique mechanistic insights into the adsorption process. These findings establish a novel and clear rational design strategy for the development of high-capacity adsorbents optimized for water remediation from PFAS.

## Results and Discussion

### Synthetic Aspects and Structural Characterization of Fe-MOF-808

MOF-808, with its robust stability, high porosity and largely tunable unsaturated Zr_6_O_8_ building units (Figure 1a), serves as an ideal template for incorporating various chemical moieties, including metal species.^36–38^ As detailed in the ESI (see Sections S1, S3–S8), pristine MOF-808 was synthesized using well-reported protocols and was characterized in detail to confirm successful preparation.^28^ Postsynthetic incorporation of iron into the framework was achieved by treating the as-made material with a methanolic solution of Fe^II^ acetate at 60 °C for 24 h (Section S1). The use of methanol not only provides the required solubility for the metal salt, but also facilitates the removal of formate ligands from the Zr_6_O_8_ clusters in MOF-808, as demonstrated in previous studies.^39,40^ Additionally, the higher basicity of the acetate ion compared to other counterions in Fe salts (e.g., chloride, nitrate) provides a more favorable environment to equilibrate the positive charges following formate removal.^36^ Consequently, this metalation strategy led to the incorporation of 3.65 Fe atoms per Zr_6_ cluster node, as determined by inductively coupled plasma optical emission spectroscopy (ICP-OES, see Section S2). This loading is higher compared to previous attempts to insert iron using other salts in MOF-808 (ranging from 0.5 to 1.2 Fe atoms per Zr_6_ node^41,42^ ) or similar Zr-MOFs like NU-1000 (with Fe loadings ranging from 0.33 to 2.2 Fe per Zr_6_ node).^43,44^

**Figure 1:**
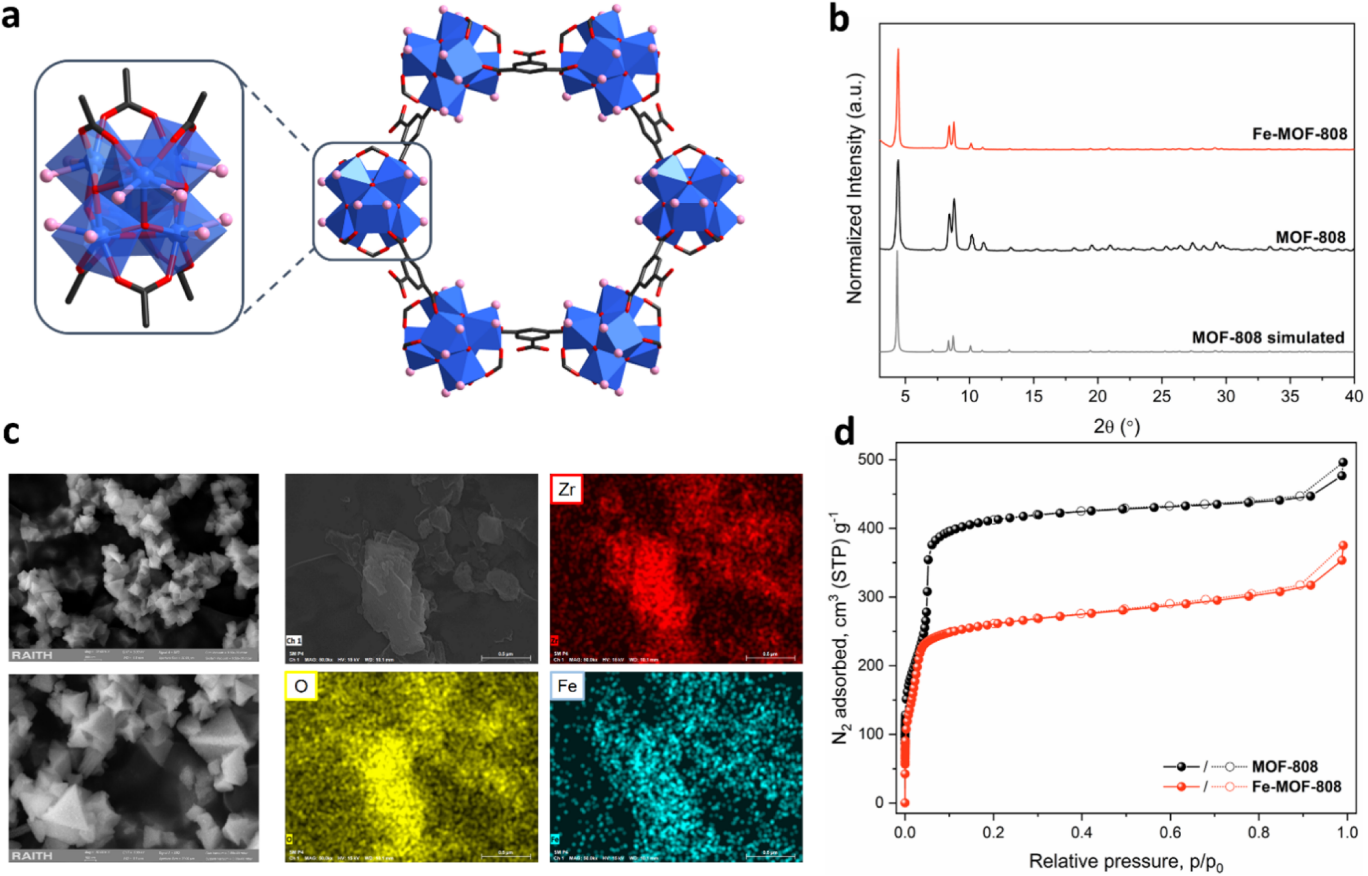
(a) Structure of the unsaturated Zr_6_O_8_ cluster node and resulting hexagonal pores in MOF-808. Color coding: Zr = blue, C = gray, O = red. Pink spheres indicate the potentially available positions for node functionalization. Hydrogen atoms have been omitted for clarity. (b) PXRD data of all synthesized frameworks, along with the calculated pattern for MOF-808. (c) Left: SEM images of the crystallites in MOF-808 (top) and Fe-MOF-808 (bottom). Right: Elemental mapping analysis of the spatial distribution of Zr (red), O (yellow) and Fe (light blue) in Fe-MOF-808. (d) N_2_ sorption isotherms (at 77 K) of the pristine and Fe-MOF-808 frameworks, showing a reduction in uptake following metalation.

Powder X-ray diffraction (PXRD) studies confirmed that the material remains highly crystalline after this treatment, while retaining the structural integrity of the main framework (Figure 1b and Section S3). The lack of additional Bragg peaks also confirmed the high purity of the solid, without the formation of other phases such as iron oxides. Scanning Electron Microscopy (SEM) images as seen in Figure 1c and Section S5 showed that the morphology (octahedral shape) and size (200–400 nm) of the crystallites^45^ are also left unaltered postmetalation. Moreover, Energy Dispersive X-ray (EDX) mapping analysis in Fe-MOF-808 evidenced the homogeneous distribution of iron throughout the particles (Figure 1c), consistent with the placement of Fe sites near the Zr_6_ node locations. This was further verified by N_2_ sorption experiments at 77 K (Figure 1d and Section S7), which showed considerable decreases in gas uptake (from 476.5 to 353.5 cm^3^/g at 0.99 p/p_0_) and specific surface area values (from 1678 to 1026 m^2^/g) for functionalization. Analysis of the isotherms shown in Figures S14–S15 revealed a reduction in pore size and volume contribution for the hexagonal mesopore of Fe-MOF-808, indicating successful insertion of the metalated species into the Zr_6_ clusters associated with these spaces. Additional chemical analysis using proton Nuclear Magnetic Resonance (^1^H NMR) determined that the majority of formate ligands are removed from the framework during the postsynthetic treatment, while *ca*. 1 acetate moiety per Fe center is introduced (Figure S5). The resulting formula of Zr_6_Fe_3.65_­O_4_(OH)_4_­(BTC)_2_­(HCO_2_)_0.59_­(OAc)_3.38_­(H_2_O)_12.98_­(OH)_12.98_, where BTC = benzene-1,3,5-tricarboxylate, was found to be consistent after characterizing several different batches of Fe-MOF-808 using the above measurements.

A series of synchrotron-based characterization techniques along with Density Functional Theory (DFT) calculations were performed to precisely determine the location and structural environment of the iron centers within the functionalized framework (Figure 2a–c). Initially, X-ray total scattering data suitable for pair distribution function (PDF) analysis were collected for both materials as detailed in Section S9. Figures 2b and S18 show dominant short-range signals that are characteristic of the Zr_6_O_8_ node (∼2.2, 3.5, and 5.0 Å, corresponding to Zr–O, Zr···Zr and Zr···Zr_axial_ distances, respectively).^46^ The contribution of the iron species was then isolated through a differential analysis (dPDF), obtained by subtracting the data of pristine MOF-808 from that of Fe-MOF-808. The resulting dPDF profile (Figure 2b) revealed several peaks which indicate formation of binuclear iron-oxo clusters that bridge neighboring Zr_6_O_8_ nodes, binding in positions previously occupied by the now-removed nonstructural formate ligands. A similar configuration has been previously observed when reacting MOF-808 with FeCl_2_, albeit resulting in different metal loading and in additional coordination of chlorine atoms to the iron centers.^41^ In excellent agreement with this model, the dPDF of Fe-MOF-808 displayed signals at ca. 2.0, 3.1, and 3.4 Å, corresponding to respective Fe–O,^47^ Fe···Fe^48^ and Fe···Zr distances. Additional peaks at 4.5 and 4.9 Å matched well with the expected distances between iron sites and terminal oxygen atoms from ^–^OH/H_2_O species within the Zr_6_ node.

**Figure 2:**
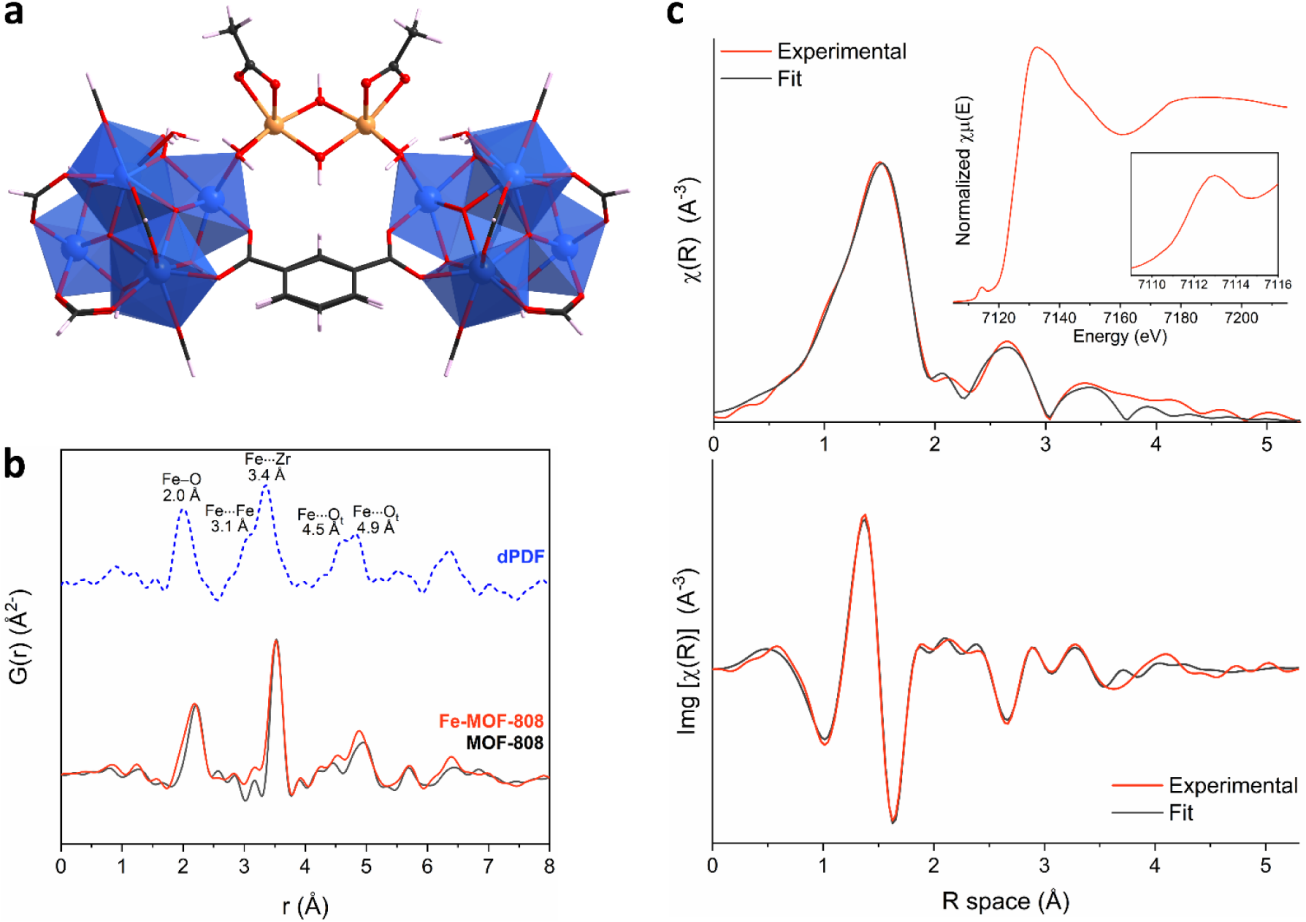
(a) DFT computed structure of the binuclear iron–hydroxo clusters in Fe-MOF-808. Additional details on the calculations can be found in the ESI. (b) PDF data of MOF-808 and Fe-MOF-808, along with the corresponding dPDF signal (blue) which isolates the contribution of the iron species. (c) *k* ^2^-weighted χ­(r) (top) and Img­[χ­(r)] (bottom) Fe EXAFS spectra of Fe-MOF-808 along with the fit calculated from the proposed DFT model. Inset shows the corresponding normalized Fe-XANES data.

Subsequently, *ex situ* X-ray Absorption Spectroscopy (XAS) data were collected (Section S10) at the Fe *K-*edge to fully elucidate the local structure of the iron clusters in Fe-MOF-808. X-ray absorption near edge spectroscopy (XANES) analysis was first performed to gain information on the oxidation state and coordination geometry of the iron centers. The respective data show a single peak at the pre-edge region typically associated with the 1s → 3d electronic transition^49^ at ca. 7113.1 eV, with a rising edge signal at ca. 7123.6 eV (Figures 2c and S20). In agreement with the PDF results, these values are consistent with those observed in diferric complexes.^50,51^ A comparison of the pre-edge feature with well-defined reference compounds of Fe^II^ or Fe^III^ (Figure S20)^48,52,53^ further confirms that the iron centers in Fe-MOF-808 are in the +3 oxidation state. Moreover, the presence of an intense single pre-edge feature points toward a highly distorted octahedral coordination geometry, as the noncentrosymmetric environment allows for 3d-4p orbital mixing which further contributes to the peak intensity.^51,54,55^ Further analysis of the extended X-ray absorption fine structure (EXAFS) data revealed three main peaks, centered at ca. 1.5, 2.6, and 3.3 Å (phase-uncorrected), which can be attributed to Fe^III^–O, Fe···Fe and Fe···Zr distances. These values are in excellent agreement with the PDF data and previous reports.^41,52,56^ These results strongly support the incorporation of octahedral Fe^III^-oxo dimers within the Zr_6_O_8_ clusters of the MOF-808 structure.

Density Functional Theory (DFT) calculations were performed (Section S11) in order to elucidate the possible conformations of the iron bimetallic core of the Fe-MOF-808. The starting configuration of the iron core is taken from our previous work, where chlorine atoms were coordinated to the iron sites.^33^ Three different models were constructed by considering different binding modes of the acetate ligands to the iron bimetallic core, which also contains two bridging hydroxide anions. The structures of the optimized models are presented in the ESI (Figure S24). The most stable conformation is presented in Figure 2a, where each acetate ligand is connected in a bidentate mode to an iron atom. The Fe–O, Fe···Fe and Fe···Zr distances of the optimized geometry are computed at ∼2.0, 3.2, and 3.4 Å, in excellent agreement to the PDF and EXAFS results (Figure 2b–c). The other two configurations are energetically higher by 4.1 and 15.9 kJ/mol, with the important structural properties being significantly different than the experimentally observed ones.

### PFOA Adsorption Tests

An extensive series of experiments was conducted to thoroughly evaluate the PFOA adsorption performance of Fe-MOF-808 (Section S12). In these tests, a fixed dosage of MOF sample was added to an aqueous PFOA solution of known concentration at room temperature, and the mixture was allowed to equilibrate for 24 h. Quantitative ^19^F-NMR analysis was then performed on the solution using a trifluoroethanol (TFE)/D_2_O internal standard, to determine the remaining amount of contaminant.^57,58^ Initial screening experiments were performed at pollutant concentrations potentially found in water systems near industrial sites ([PFOA]_0_ = 100 ppm), to begin assessing the sorbent’s capabilities under environmentally relevant conditions. The results showed that Fe-MOF-808 is capable of completely removing PFOA (Figures 3a, S30 and Table S8), a significant improvement over the performance of pristine MOF-808, which only removed 30% of the contaminant.^28^ This was achieved using relatively low material amounts (0.15 g/L, with the typical dosage in MOFs ranging from 0.1 to 10 g/L).^22^ Moreover, the material can be easily reused for up to eight PFOA sorption cycles without appreciable losses in removal efficiency, by simply washing the solid with water and acetone to reactivate its pores (Figure S31). Further tests were performed to investigate the selectivity of Fe-MOF-808 toward PFOA in the presence of common ionic species (Cl^–^, NO_3_^–^, SO_4_^2–^, Na^+^, Ca^2+^, Mg^2+^, Fe^3+^, Al^3+^). As shown in Figure 3b, the material retained high PFOA removal efficiency at relatively low concentrations of competing ions (0.3–0.8 mM) regardless of their nature. When the levels of all competing species were increased by 1 order of magnitude, Fe-MOF-808 still exhibited high PFOA removal (87% to >99% against several cations and anions (Cl^–^, NO_3_^–^, Na^+^, Ca^2+^, Mg^2+^ and Fe^3+^). However, Al^3+^ and SO_4_^2–^ were found to highly interfere at these concentrations, limiting PFOA removal during individual tests to 20% and 31%, respectively, and to 50% in mixed-ion solutions. These results can be attributed to competitive interactions with Zr sites, including cation exchange (Al^3+^) and coordination (SO_4_^2–^).

**Figure 3:**
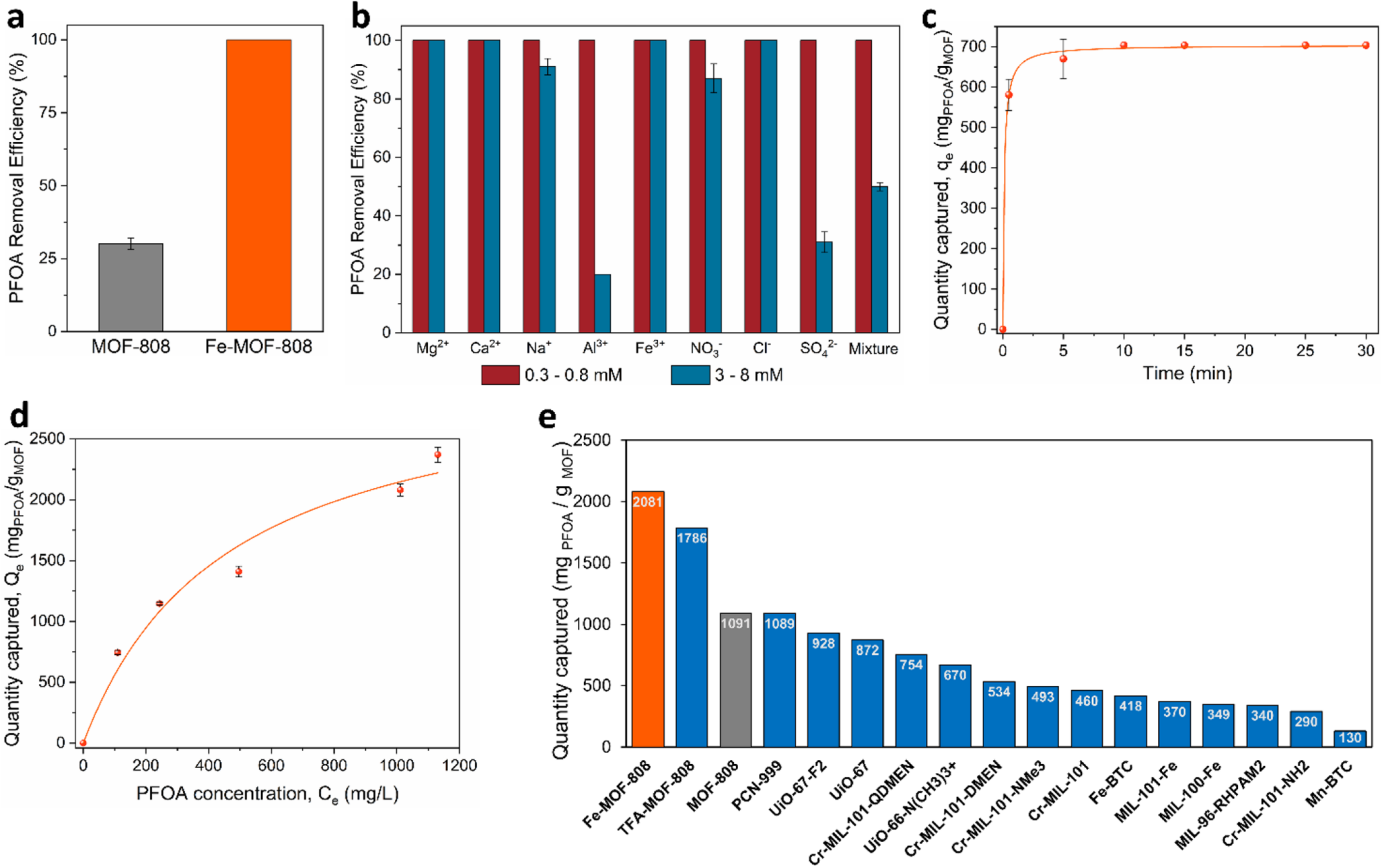
Various PFOA adsorption performance indicators for Fe-MOF-808: (a) Removal efficiency in comparison to pristine MOF-808.^28^ (b) Removal efficiencies in the presence of various ionic species. (c) Sorption kinetics, fitted using a pseudo-second order model. (d) Measured adsorption isotherm data, fitted using the Langmuir model. Experimental conditions for graphs (a–d): [PFOA]_0_ = 100 ppm, [MOF] = 0.15 g/L. All experiments were performed at least twice and average values are reported. (e) Experimental PFOA uptake of Fe-MOF-808 (C_0_ = 1000 ppm), compared to other reported MOF materials at the same conditions. Relevant data and references are detailed in the ESI (Table S15).

### PFOA Adsorption Kinetics

Time-resolved experiments revealed that Fe-MOF-808 exhibits exceptionally rapid adsorption kinetics, with 84% of the contaminant captured in less than 30 s upon contact. Removal efficiencies of 95% and 100% are then achieved within the first 5 and 10 min, respectively (Figures 3c and S32). The data fit well to a pseudo-second order model (R^2^ = 99.8% with a high adsorption rate constant (*k*_2_ = 1.32 × 10^–2^ g mg^–1^ min^–1^), indicating strong and favorable interactions between the MOF and the pollutant. These results demonstrate potential practical use of Fe-MOF-808 for PFOA remediation. Notably, the observed adsorption rate is markedly faster than the majority of MOFs reported to date (Table S12), which typically require 1–2 h to reach equilibrium, and is comparable to the ultrafast kinetics observed in NU-1000 (t_eq_ ∼ 1 min). Additionally, Fe-MOF-808 significantly outperforms other porous adsorbents such as activated carbons and resins, which often require up to 1 week to reach equilibrium.^22,59,60^

### Broad-Spectrum PFAS Capture at Environmentally Relevant Concentrations

Beyond industrial sites, PFAS are frequently detected in drinking water sources at low (ppb) levels.^12,15,61^ To further evaluate the adsorption performance of Fe-MOF-808, additional tests were performed in deionized water samples spiked with a mixture of contaminants at relevant concentrations (individual [PFAS] = 250 ppb). In this case, quantification was performed using a validated ultrahigh performance liquid chromatography with tandem mass spectrometry (UPLC-MS/MS) method as described previously.^62^ The scope of these experiments was also expanded to several PFAS classes of concern, including carboxylic acids, sulfonic acids and fluorotelomersulfonic acids. As shown in Figure S34, Fe-MOF-808 outperformed the pristine MOF-808 in all tests, clearly demonstrating the beneficial role of the secondary metal species for the capture of various PFAS. At the same time, removal efficiencies using Fe-MOF-808 remain moderate (33–67%) for PFAS containing ≤7 perfluorinated carbons, regardless of pollutant class (including PFOA). These differences in adsorption behavior indicate that, at trace concentrations, removal of PFAS is increasingly sensitive to the balance between their mobility in water and their capacity to engage in interactions with the sorbent. This is especially evident in shorter-chain PFAS, where the lower fluorine content weakens both hydrophobic and coordinative interactions with the MOF,^63,64^ likely altering adsorption mechanisms. Consistent with trends observed in other sorbents for these molecules, this effect governs performance even at higher concentrations, as shown by comparative capture tests for perfluorobutanoic acid (PFBA) and perfluorohexanoic acid (PFHxA) in ppm levels (Table S13). In contrast, Fe-MOF-808 shows much stronger performance for longer-chain PFAS, with excellent removal efficiencies of 78 to 94% for molecules of high environmental concern such as perfluorooctanesulfonic acid (PFOS) (93% removal) and 8:2-fluorotelomersulfonic acid (FTSA) (93% removal). Although this simplified matrix does not account for species found in more complex natural waters (e.g., dissolved ions, natural organic matter), the collective results support the initial potential for Fe-MOF-808 as a broad-spectrum PFAS sorbent for the capture of structurally diverse analogues.

### Maximum PFOA Adsorption Capacity of Fe-MOF-808

Encouraged by the promising capture results, we next investigated the maximum adsorption capacity of Fe-MOF-808. Adsorption isotherms were measured using the same MOF dosage in PFOA solutions of varying concentrations. To avoid any adsorption enhancing effect associated with micelle formation,^22^ all experiments were performed well below the critical micelle concentration of PFOA, which has been reported in the 3460–15696 ppm range.^60,65–67^ In the present study, the maximum pollutant concentration was limited to ∼1200 ppm, ensuring accurate measurements without external influence. Such high PFAS concentrations are still regularly encountered in fire-fighting foams, textile agents and several other consumer products.^68^ These experimental conditions further enabled the elucidation of key structure–property relationships and sorbent characteristics for practical use, including saturation limits, stability and material longevity. As shown in Figure 3d and Table S14, Fe-MOF-808 exhibits remarkably high experimental uptakes of PFOA even at elevated concentrations with a value of 2081 mg PFOA g^–1^ at ∼1000 ppm PFOA. Placing this result into context, the highest performing known MOFs at these experimental conditions (e.g., UiO-66-N­(CH_3_)_3_^+^ ^24^, UiO-67 ^26^, UiO-67-F2 ^26^, PCN-999 ^29^, Cr-MIL-101-QDMEN,^23^ TFA-MOF-808 ^28^) reported uptake values of up to 1786 mg PFOA g^–1^ (Figure 3e and Table S15).

The isotherm data were well fitted (R^2^ ∼ 98%) by either the Langmuir or Freundlich adsorption models (Figures 3d, S35–S36). While these models are based on different idealized assumptions, the above results are consistent with the proposed multi-interaction adsorption mechanism for Fe-MOF-808. In this context, the Langmuir model describes the apparent site saturation behavior and is consistent with the strong affinity of Fe-MOF-808 for PFOA. On the other hand, the high fitting value for the Freundlich model also indicates a heterogeneous adsorption environment with a broad distribution of adsorption energies across multiple sites. Considering that pore filling in this MOF can only occur via the hexagonal pore, such a behavior implies coexistence of multiple driving forces. Similar phenomena have been reported in materials that show PFAS adsorption via combined pollutant···MOF interactions, such as NU-1000 ^33^ and TFA-MOF-808 ^28^. The specific interactions within Fe-MOF-808 are discussed in detail in later sections. The Freundlich model indicates respective *K*_*f*_ and *1/n* values of 73.05 and 0.48, while a maximum adsorption capacity of 3120 mg g^–1^ was determined via Langmuir fitting. With the overwhelming majority of reported MOFs adopting the latter model, the calculated capacity represents a large improvement (Table S15), including a 97% increase over the performance of pristine MOF-808 (1581 mg g^–1^). In more context, the experimental and calculated uptakes place Fe-MOF-808 among state-of-the-art porous sorbents for PFOA to the best of our knowledge, including MOFs, activated carbons,^60^ β-cyclodextrin polymers,^69^ porous organic polymers^70^ and covalent organic frameworks^71^ (Table S16).

### Mechanistic Investigations—Initial Studies

Key insights into the PFOA adsorption mechanism in Fe-MOF-808 were obtained through a series of postcapture characterizations (Sections S2–S6). SEM analysis confirmed the preservation of crystallinity, with no detectable changes in particle size or morphology. Complementary EDX mapping revealed the presence of fluorine in high intensity and homogeneous distribution throughout the crystallites, consistent with the uniform incorporation of fluorine-rich PFOA molecules (Figure S11). Notably, the iron distribution also remained homogeneous after adsorption, indicating that the Fe sites are preserved despite the presence of strongly acidic guest species. This stability was corroborated by ICP analysis (Table S1), which showed no Fe leaching after the adsorption cycle (∼3.7 Fe atoms per Zr_6_ node). PXRD data (Figure S3) confirmed the retention of structural integrity, with a slight shift in Bragg peaks indicating minor unit cell expansion due to the inclusion of large PFOA molecules within the pores. FT-IR spectra (Figure S13) further supported the capture process, showing no structural degradation and featuring additional distinct PFOA bands, most notably at 1144, 1201, and 1237 cm^–1^, attributed to symmetric and asymmetric stretching of −CF_3_ and −CF_2_– groups.^28^

Together, these results indicated that PFOA adsorption in Fe-MOF-808 occurs not only via pore-filling but also through coordination to unsaturated metal sites. To investigate this hypothesis, NMR analyses in both solid- (^19^F, ^13^C) and liquid-state (^1^H, ^19^F) were performed on postcapture samples (Sections S4, S12). Regarding the ^19^F solid-state spectra (Figure S37), the characteristic signals of pure PFOA (δ regions of −83.9 and −121.1 to −128.4 ppm) also appear in Fe-MOF-808 after capture, however with a notable upfield shift (δ = −85.3 and −123.5 to −130.0 ppm). Moreover, the latter spectrum displays large overlap of the resonances corresponding to the inner -CF_2_- groups, particularly those nearer to the carboxylate group. These results confirm PFOA loading within the MOF and support the formation of additional interactions via the hydrophobic chain.^72^ After capture, the solid-state ^13^C spectra (Figure S38) for either MOF-808 or Fe-MOF-808 reveal an additional signal at the characteristic region of carboxylate group resonance (163.7 ppm), also clearly shifted in comparison to the pure PFOA pollutant (165.4 ppm). Collectively, these data verify the different local environment of PFOA after loading to Fe-MOF-808, in agreement with previous studies on MOFs reporting PFAS coordination.^31^ The liquid-state ^1^H NMR studies revealed significant changes in the nonstructural ligand environment compared to the as-made material, particularly a partial release of acetate species from the Fe_2_ cluster during adsorption.^28^ ^19^F- analyses confirmed that simultaneously, a substantial amount of PFOA is incorporated directly into the inorganic nodes, accounting for significant portion of the total uptake (Table S18). At low pollutant concentrations, this coordination-based uptake dominates over pore-filling, consistent with the presence of thermodynamically favorable binding sites. As the pollutant concentration increases, the number of PFOA molecules incorporated into the framework rises to a remarkable 5.3 per Zr_6_ cluster. This exceeds previously reported values for MOFs that rely on carboxylate bridging as the primary PFOA binding mechanism, such as PCN-999 (4 molecules/Zr_6_),^29^ TFA-MOF-808 (3.6 molecules/Zr_6_),^28^ and pristine MOF-808 (3.1 molecules/Zr_6_). In Fe-MOF-808, however, traditional bridging coordination (where the carboxylate group of the contaminant bridges adjacent Zr centers within the same cluster) is sterically hindered by the presence of Fe_2_ species, suggesting an alternative binding mode. The observed release of acetate further supports a mechanism in which PFOA coordinates directly to both Zr and Fe centers. This newly identified synergistic capture mechanism, which combines pore-filling with coordination to multiple metal sites, plays a pivotal role in the enhanced adsorption capacity and kinetics of Fe-MOF-808.

### Mechanistic Investigations—*Ex Situ and In Situ* Synchrotron Studies

PDF analysis of Fe-MOF-808 after PFOA capture further supports the incorporation of the contaminant within the framework (Figure 4a). The differential PDF (dPDF), obtained by subtracting the precapture PDF signal from that of the material after capture, reveals additional interatomic correlations not present in the pristine material. Notably, peaks corresponding to C–F and F···F distances, characteristic of fluorocarbon chains, confirm the interaction of PFOA molecules with the MOF. Furthermore, subtle but discernible changes are observed in the metal–oxygen (M–O) region of the PDF around 2 Å, consistent with modifications in the coordination environment at the Zr and Fe nodes. These features confirm the presence of PFOA within the structure and suggest strong interactions with the MOF framework. The appearance of such signals, alongside preserved framework correlations, indicates the coexistence of guest–host interactions and structural stability, supporting a capture mechanism that involves both pore confinement and coordination at the metal nodes.

**Figure 4:**
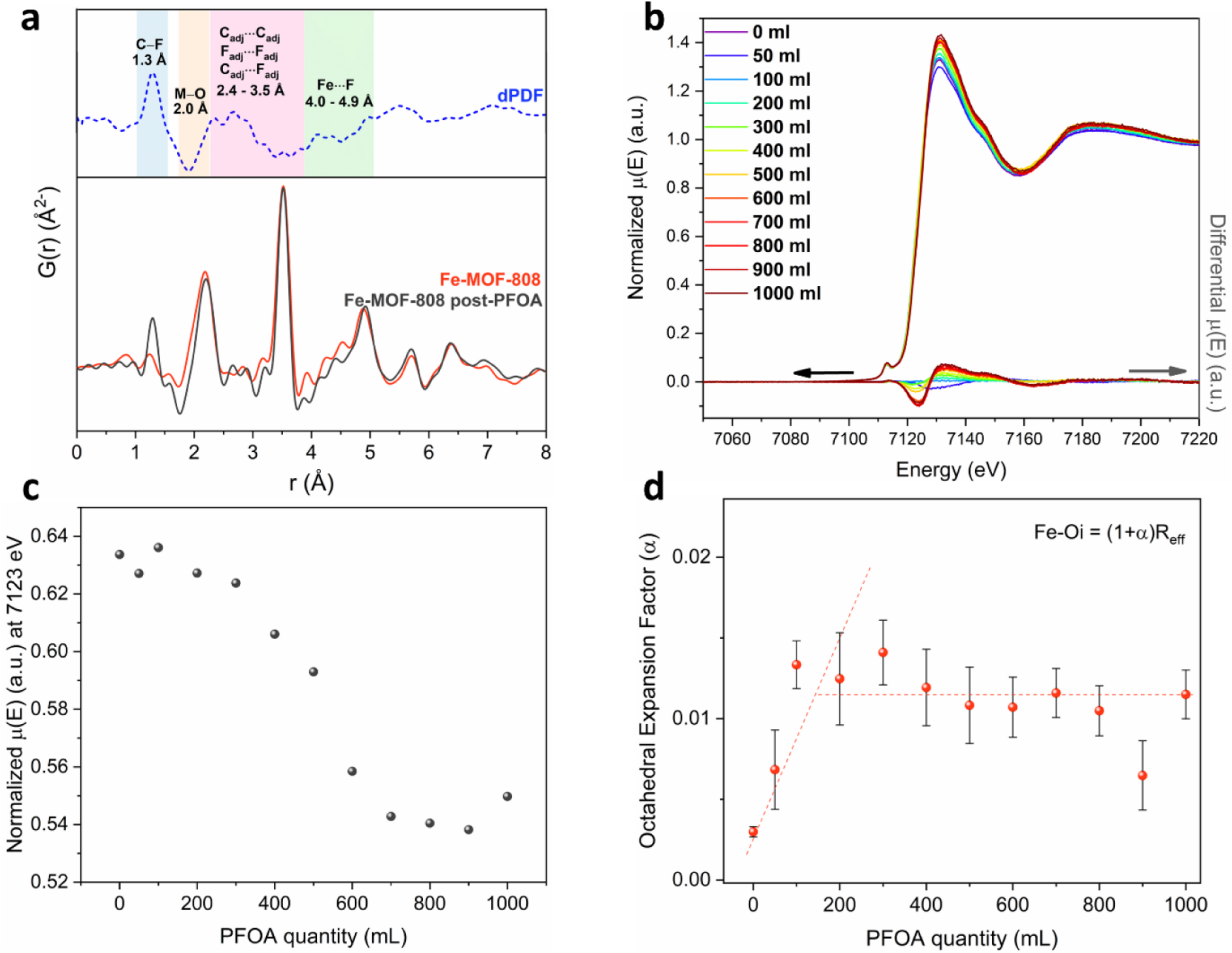
(a) PDF data of Fe-MOF-808 before and after treatment with PFOA. Differences in the highlighted M–O region reveal modifications in the coordination environment of the Zr and Fe centers. The corresponding dPDF signal (blue) confirms the presence of PFOA within the MOF and evidence formation of stabilizing weak interactions. (b–d) *In situ* XAS data analysis (Fe K-edge) for Fe-MOF-808 at the addition of specific volumes of a 100 ppm aqueous PFOA solution, depicting: (b) the XANES region, including the respective differential absorption. (c) The corresponding intensity values at 7123 eV, as a function of the PFOA quantity. (d) Respective changes to the alpha factor, related to expansion/contraction of the Fe–O octahedron.

*Ex situ* XAS characterization of Fe-MOF-808 at the Fe *K*-edge, before and after capture (Figure S21), reveals that the pre-edge peak does not shift in energy, indicating no significant alterations in overall coordination environment and geometry of Fe between the two materials. Furthermore, the postcapture material displays a much higher intensity in both the white line and EXAFS regions, along with a slight increase in pre-edge intensity and a small (*ca*. 0.5 eV) rightward shift in the rising edge. Given the nature of the structure and the chemical processes involved, these variations are unlikely to indicate a change in oxidation state. Instead, a more plausible explanation is subtle electronic modifications in the local structural environment, as highly acidic and electronegative PFOA molecules approach the Fe sites and exchange with acetate species (p*K*_a_ = 4.76), coordinating to the metal centers.^73–75^

To further validate these findings, *in situ* XAS studies at the Fe *K*-edge were conducted by incrementally introducing a 100 ppm aqueous PFOA solution to an MOF sample. As noted in previous sections, the capture kinetics are rapid and occur much faster than the time required for optimal data acquisition. Consequently, these experiments provide snapshots of fully completed pollutant capture, while the incremental addition of PFOA allows for the study of progressive occupation of remaining adsorption sites within the MOF.

As shown in Figure 4b, the corresponding differential XANES clearly shows two points of major variation (located at 7123 and 7136.5 eV), indicating the localized electronic changes. By plotting the normalized intensity at the former energy position (7123 eV, Figure 4c) as a function of the pollutant quantity exposed to the sample, it is observed that these changes reach a plateau after the addition of approximately 600 mL of solution due to saturation. These observations suggest that the Fe centers undergo incremental ligand field changes as acetate ligands are gradually replaced by PFOA molecules. The saturation effect also aligns with the mechanism suggested by theoretical modeling (as discussed below), where PFOA coordination to iron occurs predominantly at higher concentrations. This interaction enhances orbital mixing and increases absorption intensity, without altering the Fe oxidation state.^76,77^ Once ligand exchange is largely complete, the electronic environment stabilizes.

Moreover, fitting of the *in situ* EXAFS data to the structural model of the Fe_2_ dimer (Figure S23 and Table S5) revealed slight dynamic fluctuations in the Fe–O distances, further indicating changes in charge transfer due to the exchange of acetates with PFOA in the coordination environment of the Fe centers. As shown in Figure 4d, the addition of PFOA initially causes an expansion of the octahedron around the Fe sites, correlating with the coordination of pollutant molecules, then its expansion parameters stabilize as PFOA amount increases. The observed charge transfer alterations are further supported by the increase in orbital mixing and peak intensities observed in the XANES data. Overall, the synchrotron XAS results indicate that PFOA adsorption induces subtle electronic and structural changes at the Fe centers without changes in oxidation state and coordination geometry. Taken together with the synchrotron PDF data, the results strongly support a mechanism in which Fe sites participate directly in adsorption through PFOA coordination, leading to associated changes in the local coordination environment. To gain further insight into the capture mechanism at low concentrations, computational modeling approaches were employed.

### Mechanistic Investigations—Theoretical Calculations

A multiscale computational methodology (Section S11) is applied in order to investigate the interactions of the PFOA molecules with the Fe-MOF-808, and underpin the effect of the iron metal sites. The methodology ranges from Density Functional Theory (DFT) calculations for the calculation of intermolecular interactions and thermodynamic properties, to Grand Canonical Monte Carlo (GCMC) simulations for the estimation of the PFOA uptake of the Fe-MOF-808 and Molecular Dynamics (MDs) simulations for the distribution of the PFOA molecules inside the pores of the MOF.

DFT calculations were initially performed to evaluate the energetics and thermodynamics associated with the successive addition of up to three PFOA molecules to the Fe-MOF-808 model (Table S6 and Figure S25). The computed free energies shed light into the spontaneity of the possible reactions and identify the most favorable adsorption sites. Three possible scenarios were considered: (i) a PFOA molecule is bound to two zirconium sites in a bidentate manner by displacing a hydroxide anion and a water molecule from the node and releasing two water molecules, (ii) a PFOA molecule is bound to a zirconium site by displacing a hydroxide anion from the node and releasing a water molecule, or (iii) a PFOA is bound to an iron atom by displacing an acetate ligand and liberating a molecule of acetic acid. Scenario (ii) is predicted to be more exothermic than (i), but after accounting for entropic contributions both scenarios exhibit comparable free energies. This is because of the significant entropic contributions from the liberation of an additional water molecule in case (i). For clarity, the results corresponding to scenarios (ii) and (iii) are presented in Figure 5. For the first PFOA addition, adsorption at the zirconium site is calculated to be more favorable, with a reaction free energy of −89.3 kJ/mol compared to −35.7 kJ/mol for the iron site. In the case of double PFOA addition, full coverage of the iron sites yields a reaction free energy of −81.8 kJ/mol, while double coverage of zirconium sites results in −189.9 kJ/mol. When both iron and zirconium sites are occupied, the reaction free energy is calculated −115.2 kJ/mol. These results suggest that zirconium sites are more preferable than iron sites for PFOA adsorption. In the final case involving the binding of three PFOA molecules, two configurations were evaluated. The coverage of two zirconium sites and one iron gives a reaction free energy of −210.2 kJ/mol, while the coverage of two iron sites and one zirconium gives a higher free energy change of −145.7 kJ/mol. This suggests that zirconium sites are stronger binding sites than iron. Moreover, partial replacement of one acetate by PFOA is more favorable than full replacement of both acetates, in perfect agreement to the experimental findings.

**Figure 5:**
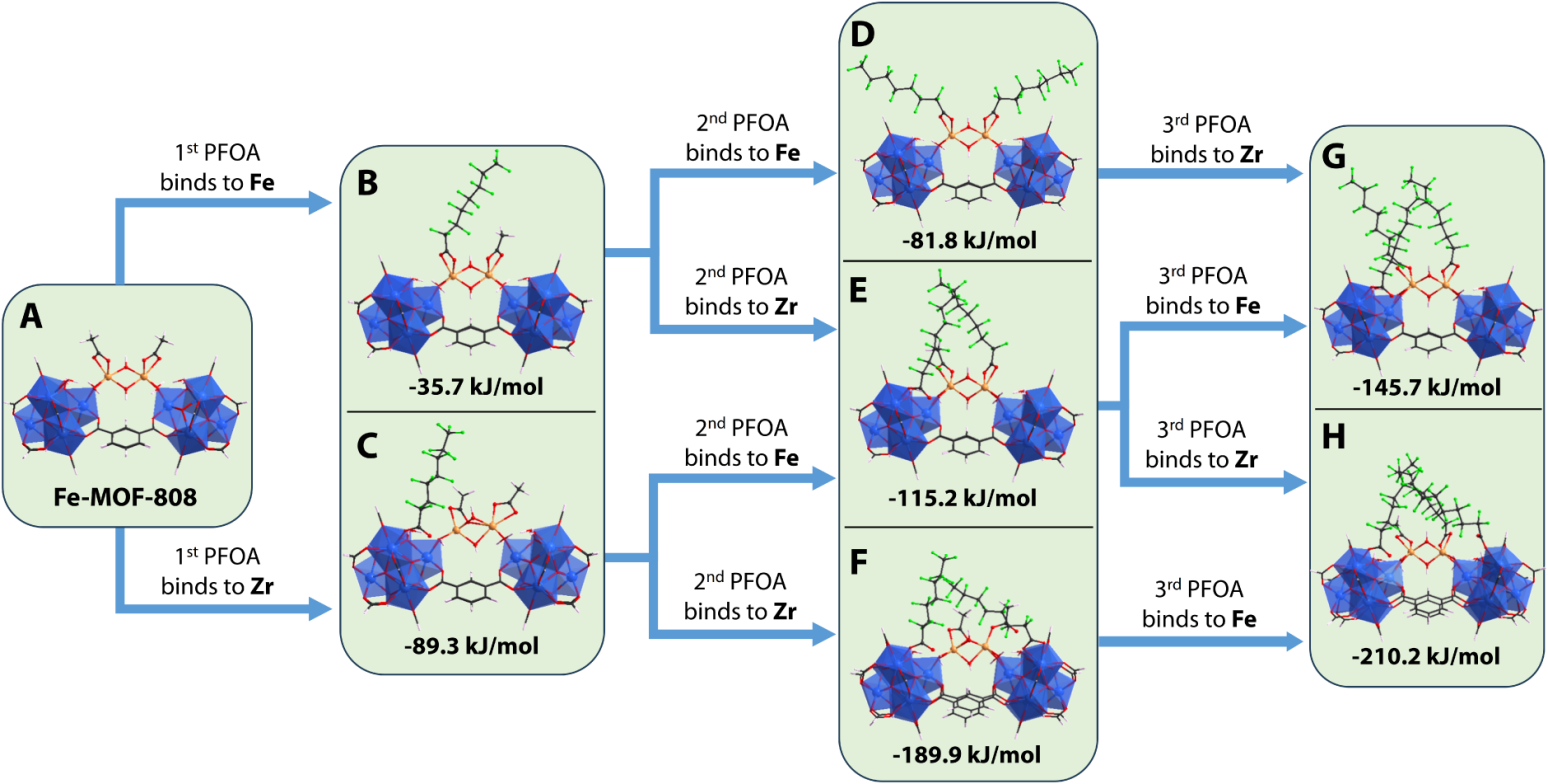
Free energy reaction profile for the successive addition of up to three PFOA molecules to the Fe-MOF-808 model. Total reaction free energies are given in black. All energies are in kJ/mol.

To further investigate the role of iron centers, analogous calculations were performed for the addition of two PFOA molecules to the pristine MOF-808 model. In this case, the reaction free energy for adsorption at zirconium sites is calculated to be −151.6 kJ/mol, which is less favorable than the −194.5 kJ/mol obtained for the Fe-MOF-808 system. This indicates that, although iron atoms are not the primary binding sites, their presence significantly enhances the interaction between PFOA and the zirconium centers.

To further support this conclusion, GCMC simulations are performed to estimate the PFOA uptake of pristine MOF-808 and Fe-MOF-808. It should be noted that the Fe-MOF-808 cell used in the simulations includes four Fe_2_ sites per pore (i.e., 2 Fe per Zr_6_ core). Although this represents a lower iron incorporation than that observed experimentally, the model still provides valuable insights into the enhancement of PFOA uptake. More details about the models are given in the ESI (Section S11). The GCMC results presented in Figure 6, show that at low PFOA fugacities (i.e., low concentrations), Fe-MOF-808 exhibits a higher adsorption capacity compared to the pristine MOF-808. However, at higher concentrations, both materials demonstrate similar uptake performance. It should be mentioned that these GCMC simulations underestimate the real gravimetric capacity, because they do not account for the PFOA molecules that are chemisorbed either on the Zr_6_-nodes or on the Fe sites. Therefore, the computed values for the capacity include only PFOA molecules physisorbed in the pores. This enhanced performance at lower concentrations is attributed to the stronger interactions between PFOA molecules and the pore environment of Fe-MOF-808, as corroborated by the DFT calculations. At low pressures, only a limited number of PFOA molecules are introduced into the material, and they preferentially occupy the most energetically favorable binding sites. In Fe-MOF-808, these high-affinity sites interact more strongly with PFOA than those in the pristine material, resulting in greater uptake under low-loading conditions. At higher loading levels, PFOA uptake is primarily governed by the available free volume within the MOF structure. In this regime, the influence of specific metal sites on adsorption diminishes, as the most energetically favorable binding sites have already been occupied at lower pressures. Consequently, additional PFOA molecules are adsorbed in less selective regions of the pore space, and the overall uptake becomes dominated by the material’s porosity rather than specific host–guest interactions.

**Figure 6:**
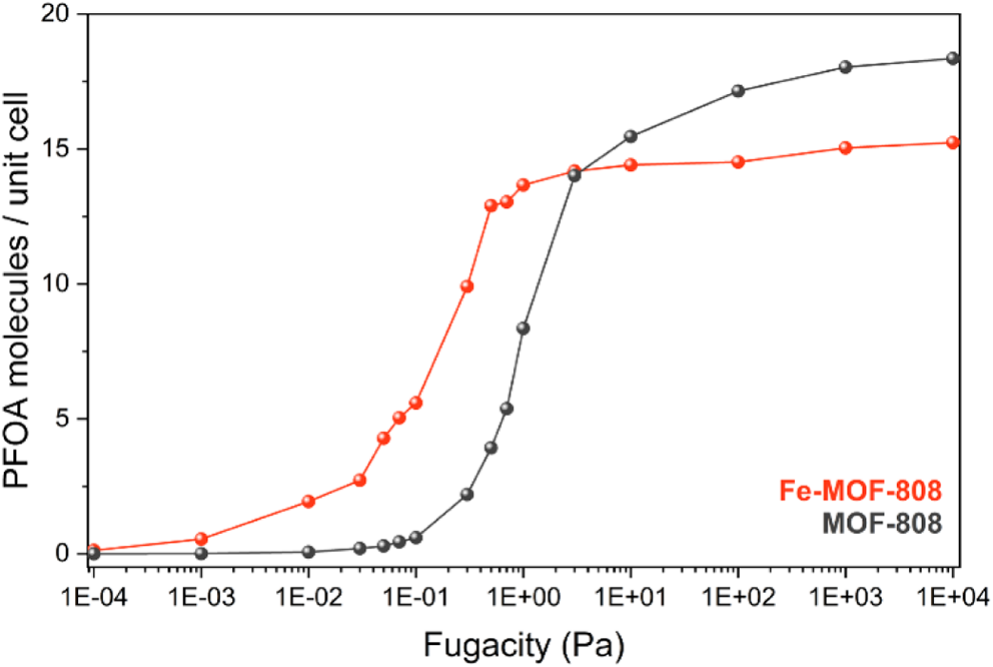
Uptake of PFOA molecules per cell at different fugacities (concentrations) for pristine MOF-808 and Fe-MOF-808 as calculated from GCMC.

Similar conclusions are drawn from the analysis of MD simulations. A key outcome of these simulations is the Radial Distribution Function (RDF) analysis, which examines the spatial correlations between the oxygen atoms of the PFOA molecules and the metal centers—zirconium and iron—used as reference points. Further details are provided in the ESI (Figures S27–S29). Upon incorporation of iron into the MOF-808 framework, the intensity of the RDF peak corresponding to interactions between PFOA oxygen atoms and zirconium centers decreases. Simultaneously, a new, high-intensity peak emerges, indicating strong interactions between PFOA oxygen atoms and the iron centers in Fe-MOF-808. This shift suggests an increased number of favorable interaction sites within the functionalized pores, allowing PFOA molecules to interact with zirconium and iron centers, thereby enhancing the overall adsorption behavior.

## Conclusion

Mechanistic investigations combining synchrotron-based characterization and multiscale theoretical modeling provide a comprehensive understanding of PFOA capture in Fe-MOF-808 and explain its enhanced performance. The adsorption process is found to proceed through a dual mechanism: coordination to open metal sites, primarily at the zirconium nodes, and confinement within the porous structure. Although zirconium sites are identified as the thermodynamically preferred binding locations, the introduction of iron centers enhances the adsorption energetics and modifies the local electronic environment, as demonstrated by *in situ* and *ex situ* XAS analyses. These results are corroborated by DFT calculations, which show stronger binding in the presence of Fe, and by GCMC simulations, which reveal improved uptake performance at low PFOA concentrations. Molecular dynamics further confirm the distribution of PFOA molecules within the pores, highlighting the cooperative role of both metal coordination and physical entrapment.

While the present work clarifies in detail the mechanistic function that enhances PFAS capture in Fe-MOF-808, several key aspects remain to be addressed to translate this material toward practical implementation. In terms of synthesis, efforts will focus on investigating scalability at gram-scale or above. This will also facilitate the realization of cost-to-performance studies against established PFAS sorbents including ion exchange resins and activated carbons. From a water treatment perspective, future work will establish long-term adsorption performance in more realistic water matrices containing natural organic matter, dissolved ions and commonly co-occurring species at environmentally relevant concentrations. Beyond the challenges and limitations of Fe-MOF-808, this study introduces secondary metal incorporation as a highly promising general design strategy to improve PFAS adsorption via multiple-interaction mechanisms in MOF materials. These principles provide a foundation for the development of next-generation tailored adsorbents with improved selectivity and capacity across PFAS species of varying chain length and chemical functionality.

## Supplementary Material



## Notes

Experimental procedures, MOF characterization methods, PDF analysis, XAS analysis; Theoretical calculation methods, and PFAS capture results (PDF)
