# Supplementary material for: Atomically Precise Engineering of Synergistic Binding Sites in a Zirconium Metal–Organic Framework for the Capture of Perfluorooctanoic Acid
Source: J Am Chem Soc. 2026 Apr 10;148(19):19672–83. doi: 10.1021/jacs.5c23392 (PMC13195660; doi:10.1021/jacs.5c23392)
Supplement: Supplementary file 1 [file ja5c23392_si_001.pdf]

Atomically Precise Engineering of Synergistic Binding Sites in a Zirconium Metal-Organic Framework for the Capture of Perfluorooctanoic Acid

Sergio Marugán-Benito[a‡], Michalis Vlachos[ab‡], Lutz Ahrens[c], Miguel Roselló-González[a], Carlo Marini[d], Jordi Prat Albert[d], Andreas Mavrandonakis[b] *, Edward Loukopoulos[aef] * and Ana E. Platero-Prats[aef] *

[a] Departamento de Química Inorgánica, Universidad Autónoma de Madrid, Madrid, 28049, Spain. E-mail: edouardos.loukopoulos@uam.es

[b] Material Science Institute of Madrid (ICMM-CSIC), Madrid, 28049, Spain. E-mail: andreas.mavrantonakis@icmm.csic.es

[c] Department of Aquatic Sciences and Assessment, Swedish University of Agricultural Sciences (SLU), Uppsala SE-75007, Sweden.

[d] ALBA Synchrotron Light Source, Cerdanyola del Vallès, Barcelona, 08290, Spain.

[e] Condensed Matter Physics Center (IFIMAC), Universidad Autónoma de Madrid, Madrid. 28049, Spain.

[f] Instituto de Catálisis y Petroleoquímica (ICP-CSIC), Madrid 28049, Spain. E-mail: ana.platero@icp.csic.es.

SUPPORTING INFORMATION

**Table of contents**	Page
Supplementary Methods	S2
Section S1. MOF Synthesis	S3
Section S2. Metalation analysis	S4
Section S3. Powder X-ray diffraction	S5
Section S4. Nuclear Magnetic Resonance spectra of MOF samples	S6
Section S5. SEM and EDX Spectroscopy	S9
Section S6. FT-IR analysis	S12
Section S7. Gas sorption measurements	S13
Section S8. Thermal gravimetric analysis	S14
Section S9. X-Ray Pair Distribution Function analysis	S15
Section S10. X-Ray Absorption Spectroscopy analysis	S17
Section S11. Theoretical Calculations	S23
Section S12. PFAS capture experiments and analysis	S39
Supplementary References	S55

Supplementary Methods

Materials. All reagents were used as received from commercial suppliers and were used without further purification. Note: the same batch of pristine MOF-808 used for this study was also used in a previous work published by our group.[1] Its relevant characterizations and PFOA capture properties are given in this work without any changes in data analysis, to provide clear comparisons to the respective behavior of Fe-MOF-808.

Inductively coupled plasma optical emission spectroscopy (ICP-OES): ICP analyses were performed using an Inducted Coupled Plasma Emission Spectrometer ICP PERKIN ELMER mod. OPTIMA 2100 DV. 3 mg of each sample was digested in 4 mL of a 1:1 H_2O_2:H_2SO_4 mixture (v:v) and taken to a 10 mL in a volumetric flask volume with distilled water.

NMR spectroscopy: ^{1}H-NMR Spectra were acquired on a Bruker Avance III HD spectrometer, running at 300 MHz. ^{19}F-NMR spectra were recorded on a Bruker Avance NEO-500 spectrometer, running at 500MHz. Each MOF sample was prepared by digesting a small portion (~5 mg) of the solid in a DMSO-d_6 (700 μL) / D_2O (100 μL) / HF (50 μL) solution. In samples necessary to quantify the amount of fluorine, fluorobenzoic acid (FBA) or trifluoroethanol (TFE) was used as the internal standard. Chemical shifts (δ) are reported in parts per million (ppm) relative to the residual solvent signal with a value of 2.50 ppm for DMSO-d_6.

Powder X-Ray Diffraction (PXRD): PXRD patterns were collected using a Bruker D8 diffractometer equipped with a copper source operating at 1600 W. The samples were ground and placed onto a borosilicate sample holder, and the surface was levelled with a clean microscope slide. The diffraction patterns were collected in continuous mode over a 2θ range of 3 to 45 degrees, with a step size of 0.02° and an exposure time of 0.5 s per step. Calculated PXRD patterns from the corresponding single-crystal data were obtained using Mercury 3.8.[2]

Scanning Electron Microscope (SEM) and Energy Dispersive X-ray Spectroscopy (EDX): Images were obtained using a HITACHI S-3000N microscope equipped with EDX analyzer Quantax EDS. All samples were previously metalated with Cr.

Fourier-transform infrared (FT-IR) spectroscopy: Spectra were recorded on a PerkinElmer 100 spectrophotometer using a PIKE Technologies MIRacle Single Reflection Horizontal ATR Accessory from 4000–450 cm^{-1}.

Gas sorption studies: Low pressure N_2 gas sorption measurements were carried out at 77 K using a Micromeritics ASAP 2020 system. Prior to analysis, the as-made samples were activated under dynamic vacuum at 150 °C for 16 hours. Pore-size-distribution (PSD) curves were obtained from the adsorption branches using non-local density functional theory (NLDFT) method for a cylinder pore in pillared clays.

Thermogravimetric Analysis (TGA): Measurements were performed using an SDT Q600 from TA Instruments equipment in a temperature range between 20 °C and 800 °C under air (100 $mL \cdot min^{-1}$ flow) atmosphere and heating rate of 10 $°C \cdot min^{-1}$.

Supplementary Note 1. MOF Synthesis

Synthesis of pristine MOF-808: In a 1 L glass bottle, 4.85 g (15.05 mmol) of $ZrOCl_2 \cdot 8H_2O$ and 1.05 g (5.00 mmol) of H_3BTC were added in 450 mL of a 1:1 DMF/formic acid solution. The mixture was sonicated until complete dissolution of the solids, then placed inside an oven at 130 ºC for 2 days. The obtained white solid was washed with DMF, water and acetone (three times with each solvent), then dried at 60 ºC for 24 hours. Yield: 2.658 g (80.11%). ^{1}H-NMR (Figure S4): 8.61 (s, 6H, 2 × BTC), 8.08 (s, 5H, 5 × HCOO). Other peaks: 7.84 (s, 0.3 H, DMF), 2.85 (s, 1H, DMF), 2.70 (s, 1H, DMF). Chemical Formula: $[Zr_6O_4(BTC)_2(COOH)_5(H_2O)(OH)_5]$

Synthesis of Fe-MOF-808: In a 10 ml glass capped vial, 60 mg (0.044 mmol) of MOF-808 were added to a 6 mL MeOH solution of $Fe(OAc)_2$ (35.5 mg, 0.204 mmol). The mixture was stirred at 60ºC for 24 hours. The resulting brown solid was collected as thin powder and was then washed with water and acetone several times (until the solution remained clear and colourless), then dried at 60 ºC for 24 hours. Yield: 62.9 mg (78.72%). The above synthetic protocol can be easily scaled up (4x) with good reproducibility (Yield: 251 mg). ^{1}H-NMR (Figure S5): 8.61 (s, 6H, 2 × H_3BTC), 8.07 (s, 0.60 H, 0.60 × HCO_2H), 1.88 (s, 10.13 H, 3.38 x AcOH) ppm. Other peaks found: 3.16 (s, 1.52 H, MeOH), 2.85 (s, 1.10 H, DMF), 2.69 (s, 1.08 H, DMF), 2.04 (s, 0.40 H, acetone) ppm. Chemical Formula: $[Zr_6Fe_{3.65}O_4(OH)_4(BTC)_2(HCO_2)_{0.59}(OAc)_{3.38}[(H_2O)(OH)]_{12.98}]$ (also using the ICP results).

Supplementary Note 2. Metalation analysis

Table S1. ICP results on the incorporation of Fe in MOF-808 for various batches.

Material	Concentration (ppm)	Fe/Zr ratio	Fe per Zr_6 cluster
Fe-MOF-808 as made (Batch 1)	Zr 48.44	0.608	3.65
	Fe 18.04		
Fe-MOF-808 as made (Batch 2)	Zr 265.70	0.623	3.74
	Fe 101.39		
Fe-MOF-808 as made (Batch 3)	Zr 52.89	0.585	3.51
	Fe 18.96		
Fe-MOF-808 post-PFOA (Batch 1)	Zr 20.55	0.617	3.70
	Fe 7.755		
Fe-MOF-808 post-PFOA (Batch 2)	Zr 48.45	0.608	3.65
	Fe 18.04		
Fe-MOF-808 post-PFOA (Batch 2)[a]	Zr 40.56	0.607	3.64
	Fe 15.07		
Fe-MOF-808 post-PFOA (Batch 2)[b]	Zr 18.13	0.517	3.10
	Fe 5.66		

[a] After 5 cycles of PFOA captures. [b] After 8 cycles of PFOA captures.

Table S2. Selected reports on MOF-808 and similar Zr-based MOFs incorporating Fe sites.

Initial MOF	Fe salt	Solvent	Fe per Zr_6 cluster	Reference
MOF-808	$FeCl_2 \cdot 4H_2O$	DMF	0.5	[3]
MOF-808	$FeCl_2 \cdot 4H_2O$	DMF	1.2	[3]
MOF-808	$Fe(acac)_3$	DMF, Et_3N	1.25	[4]
MOF-808	$Fe(OAc)_2$	MeOH	3.65	This work
NU-1000	$Fe(NO_3)_3$	MeOH	0.5	[5]
NU-1000	$FeCl_2$	MeOH	2.2	[5]
NU-1000	$FeCl_3 \cdot 6H_2O$	DMF	0.055	[6]

Supplementary Note 3. Powder X-Ray Diffraction

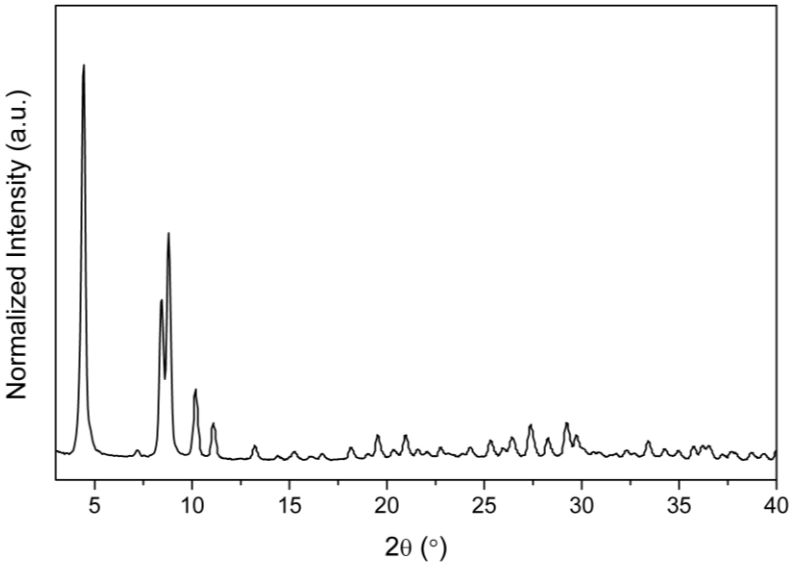

Figure S1. PXRD pattern of pristine MOF-808.

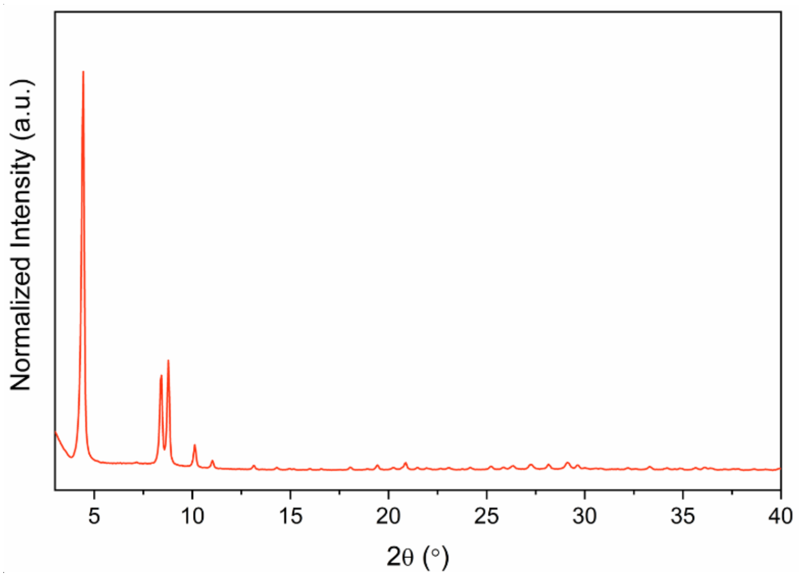

Figure S2. PXRD pattern of Fe-MOF-808.

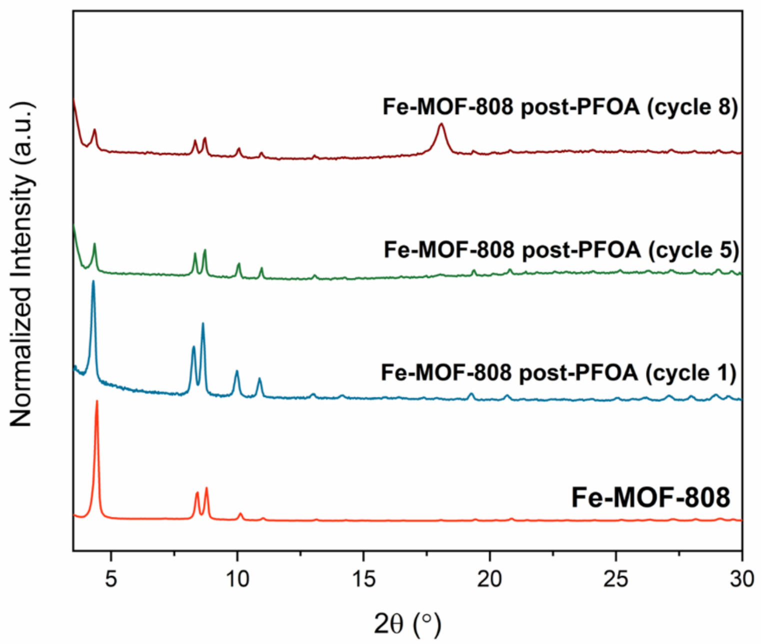

Figure S3. PXRD pattern of Fe-MOF-808, before and after PFOA adsorption (at various cycles).

Supplementary Note 4. Nuclear Magnetic Resonance spectra of MOF samples

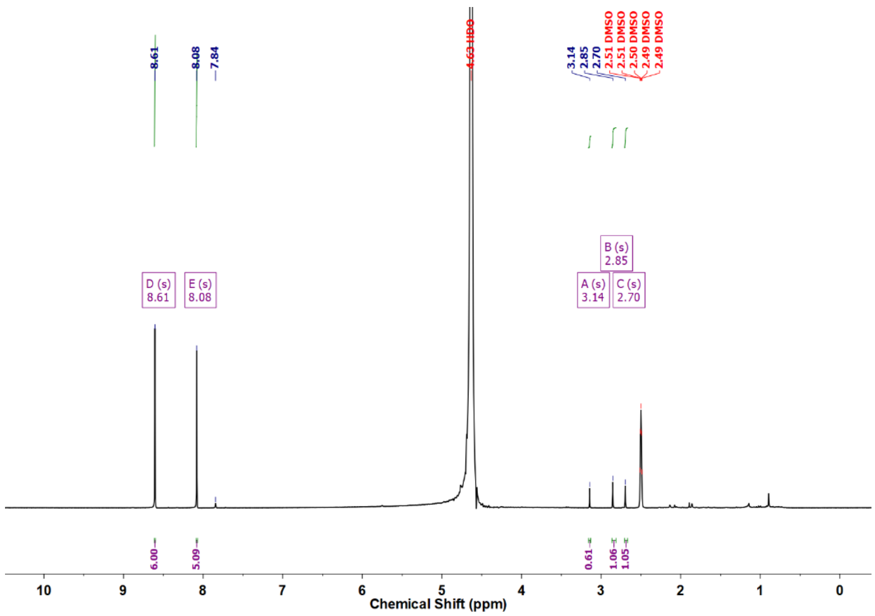

Figure S4. ^{1}H-NMR of MOF-808 in DMSO-d_6:D_2O:HF (300 MHz).

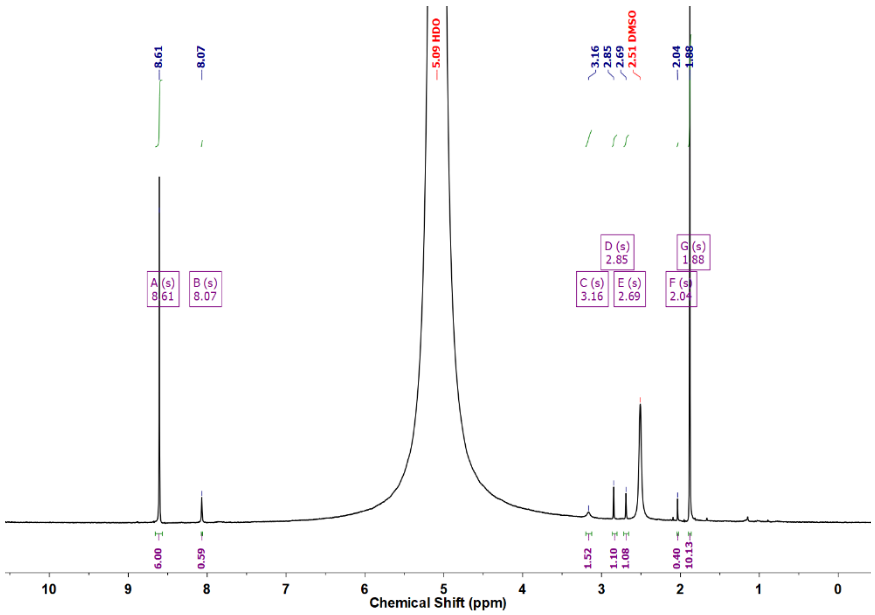

Figure S5. ^{1}H-NMR of Fe-MOF-808 in DMSO-d_6:D_2O:HF (500 MHz).

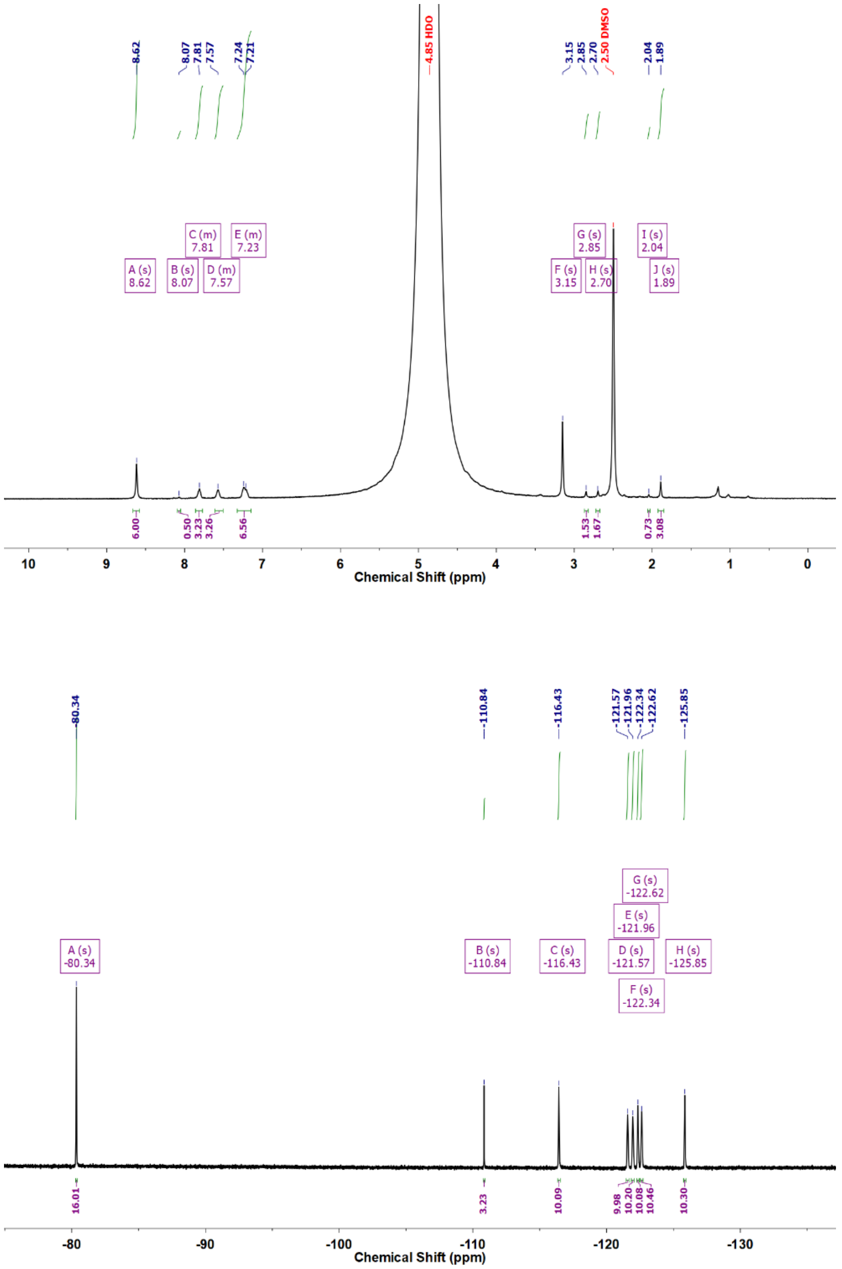

Figure S6. ^{1}H- (top) and ^{19}F-NMR (bottom) of Fe-MOF-808 after PFOA adsorption (~1150 ppm) in DMSO-d_6:D_2O:HF (500 MHz), showing the incorporation of 5.34 PFOA molecules per Zr_6 cluster. 2-fluorobenzoic acid (^{1}H-NMR peaks: all observed signals at the 7.2-7.8 ppm range, ^{19}F-NMR peak: -110.8 ppm) was used as the internal standard.

Supplementary Note 5. SEM and EDX Spectroscopy

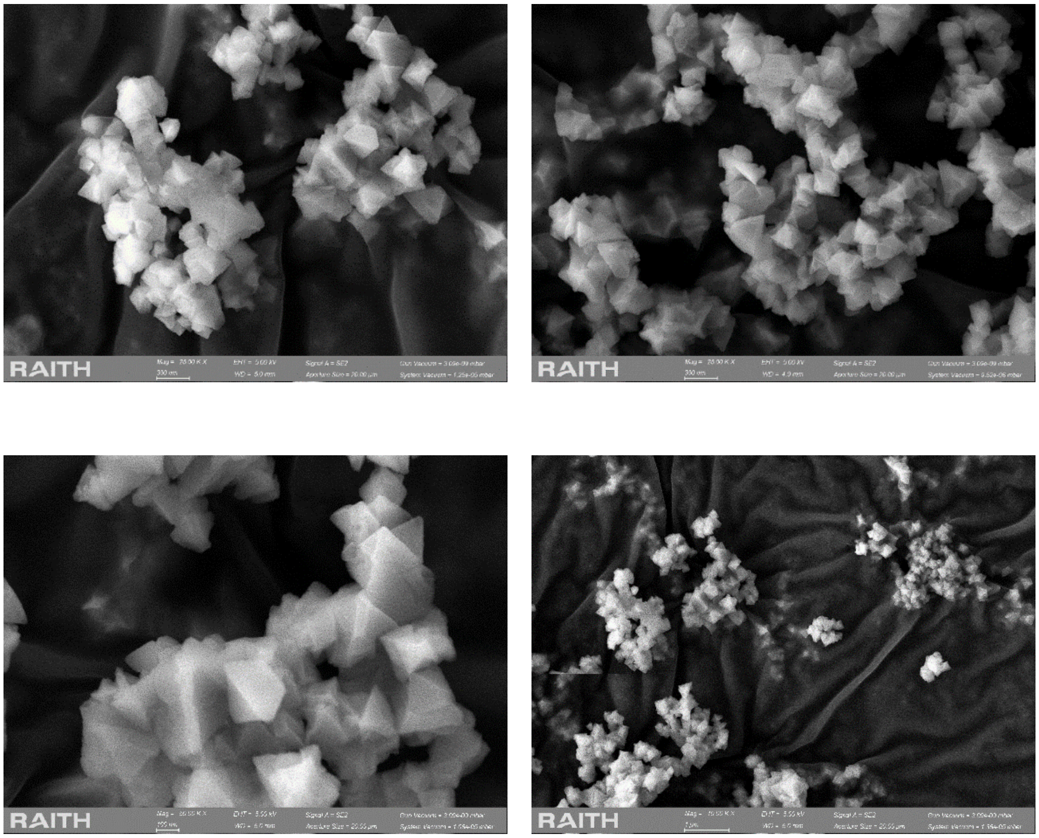

Figure S7. SEM images of pristine MOF-808.

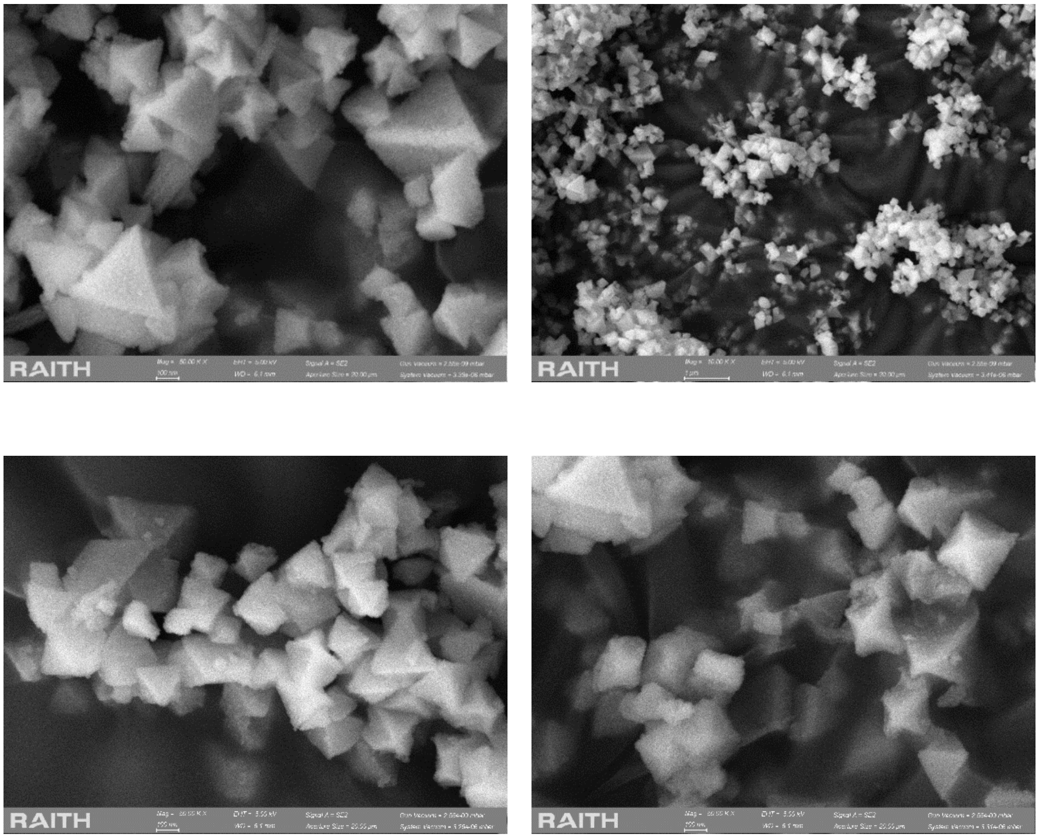

Figure S8. SEM images of Fe-MOF-808.

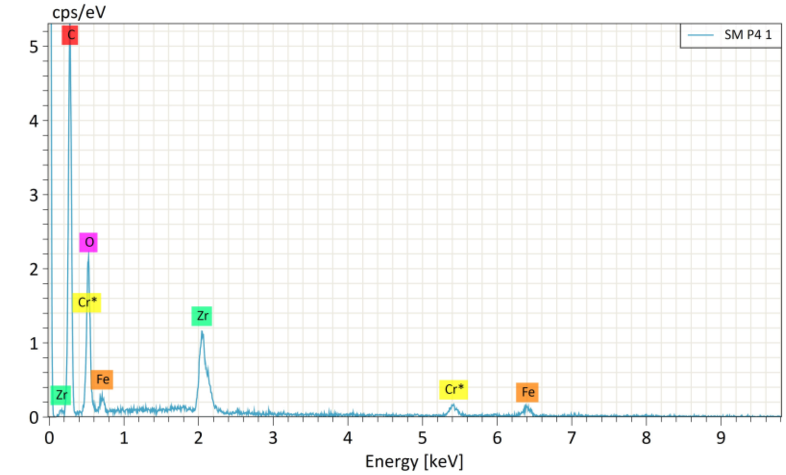

Figure S9. EDX spectrum of Fe-MOF-808 from a corresponding crystallite of Figure S8 (left).

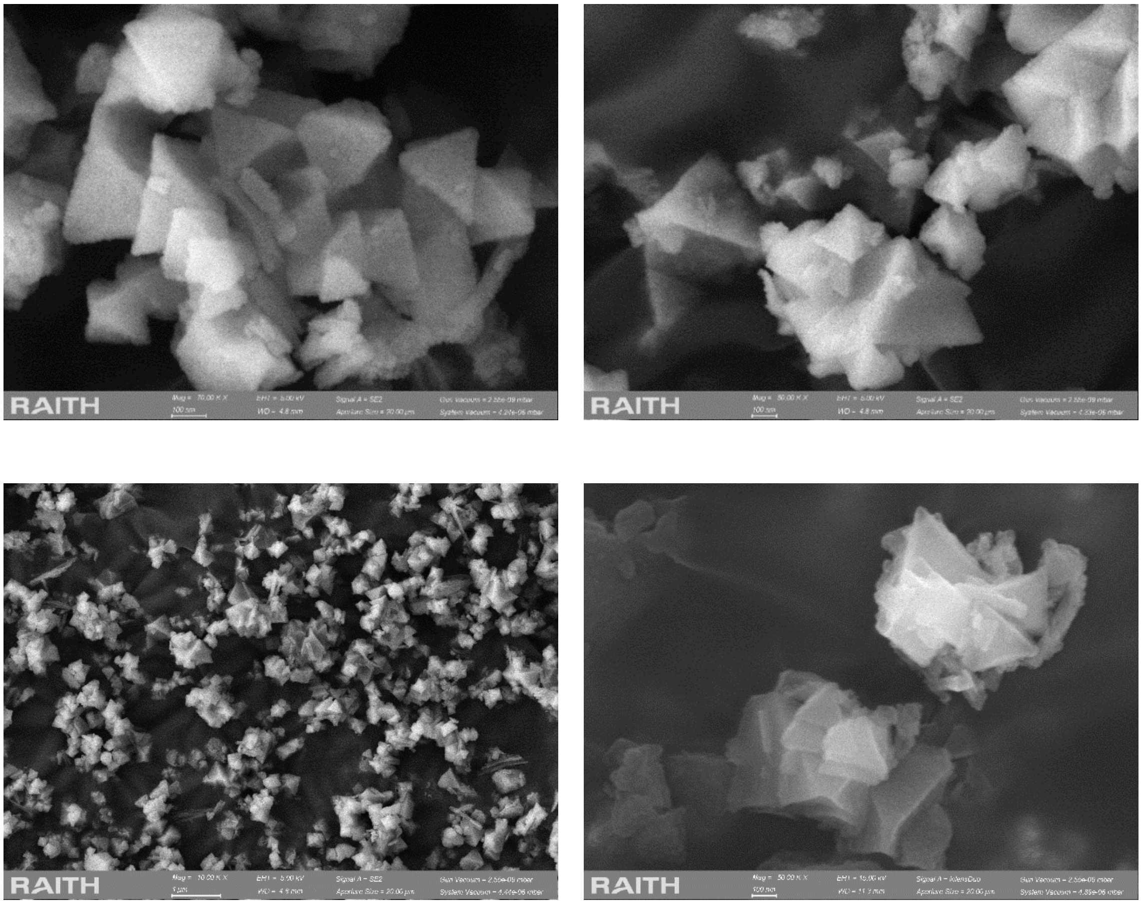

Figure S10. SEM images of Fe-MOF-808 after PFOA adsorption experiments.

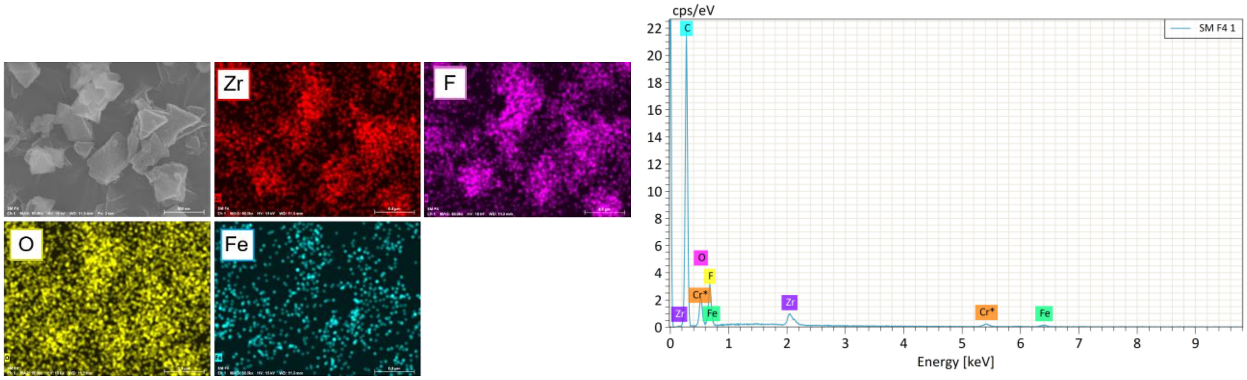

Figure S11. (left) SEM image of a Fe-MOF-808 sample after PFOA adsorption experiments, and the relevant EDX spectroscopy mappings for Zr, F, O and Fe. (right) The corresponding EDX spectrum.

Supplementary Note 6. FT-IR analysis

All MOF spectra (Figures S12-S13) display the characteristic signal of the Zr_6O_8 inorganic node, where the collective vibrations of Zr-O bonds lead to a broad peak at *ca.* 655 cm^{-1}. Other main signals are observed at *ca.* 1620 cm^{-1} (aromatic C=C bond stretching), 1560 cm^{-1}, (asymmetric COO stretching), 1380 and 1445 cm^{-1} (both due to COO symmetric stretching modes). As detailed in the main text, the spectra of a non-washed Fe-MOF-808 sample after PFOA capture shows additional signals in the typical C-F bonding region, in excellent agreement with previous reports.[1] An additional signal at 1652 cm^{-1} appears, attributed to the vibration of uncoordinated COOH, which indicates that non-coordinating PFOA molecules also occupy the pores of the framework.

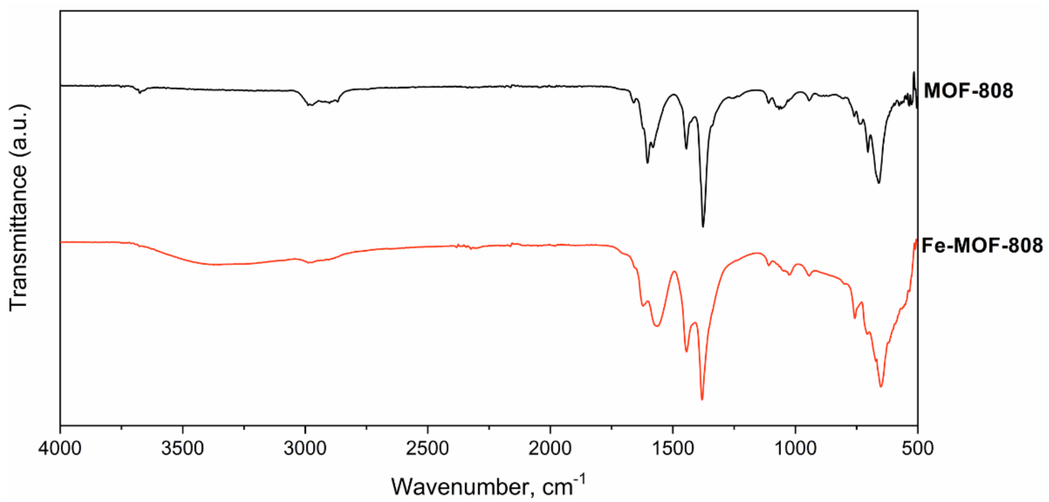

Figure S12. FT-IR spectra of all relevant materials of the MOF-808 system (as synthesized).

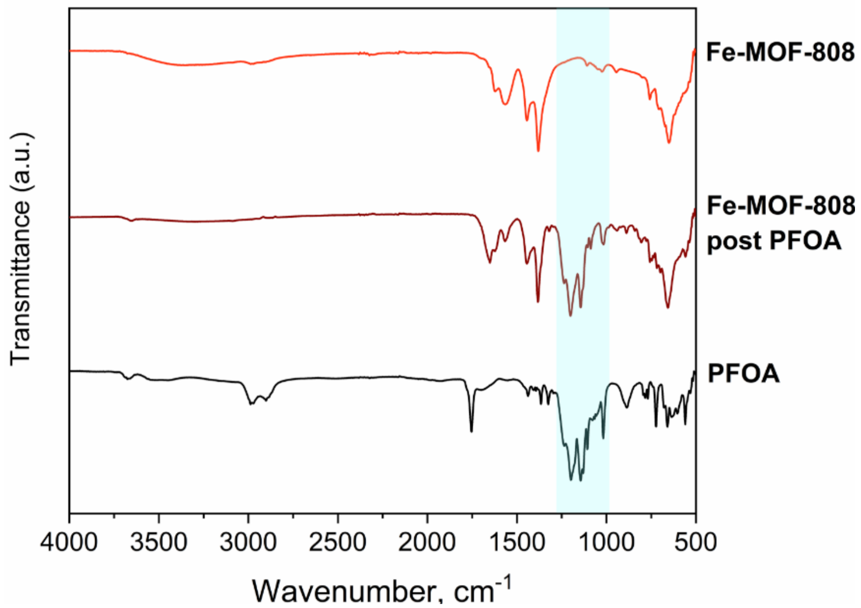

Figure S13. FT-IR spectra comparison of Fe-MOF-808 before and after capture of PFOA. The spectra of solid PFOA is included for comparison, and the characteristic signals due to C-F bonding are highlighted.

Supplementary Note 7. Gas Sorption Measurements

Table S3. Pore volume (calculated at $p/p_0 = 0.95$) and BET surface area values for the materials of this study, as calculated from the N_2 isotherms.

	**Pore volume ($cm^3\ g^{-1}$)**	**BET surface area ($m^2\ g^{-1}$)**
**MOF-808**	0.74	1678
**Fe-MOF-808**	0.54	1026

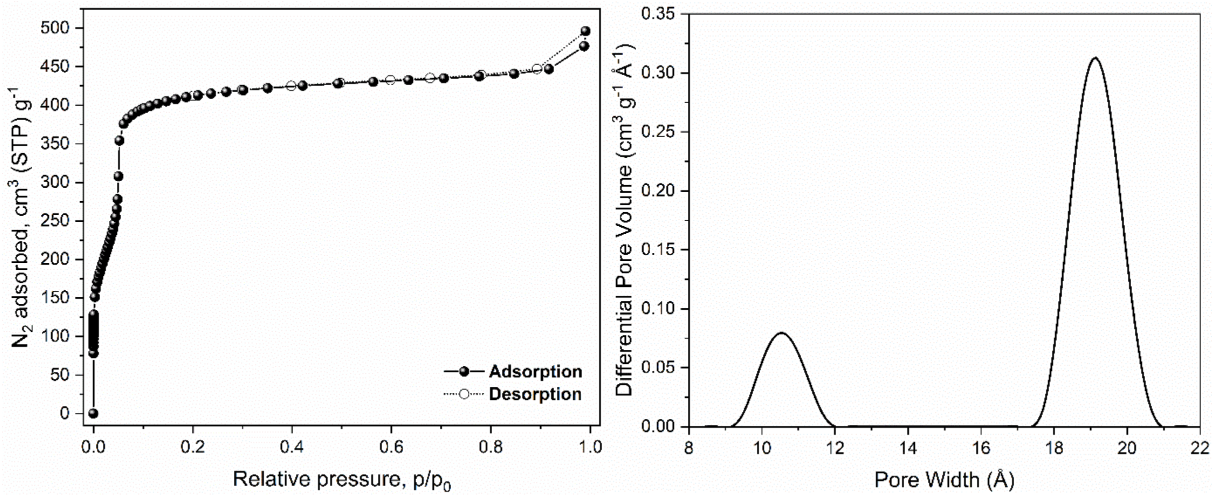

Figure S14. N_2 isotherms (at 77 K) and calculated pore size distribution analysis for pristine MOF-808.

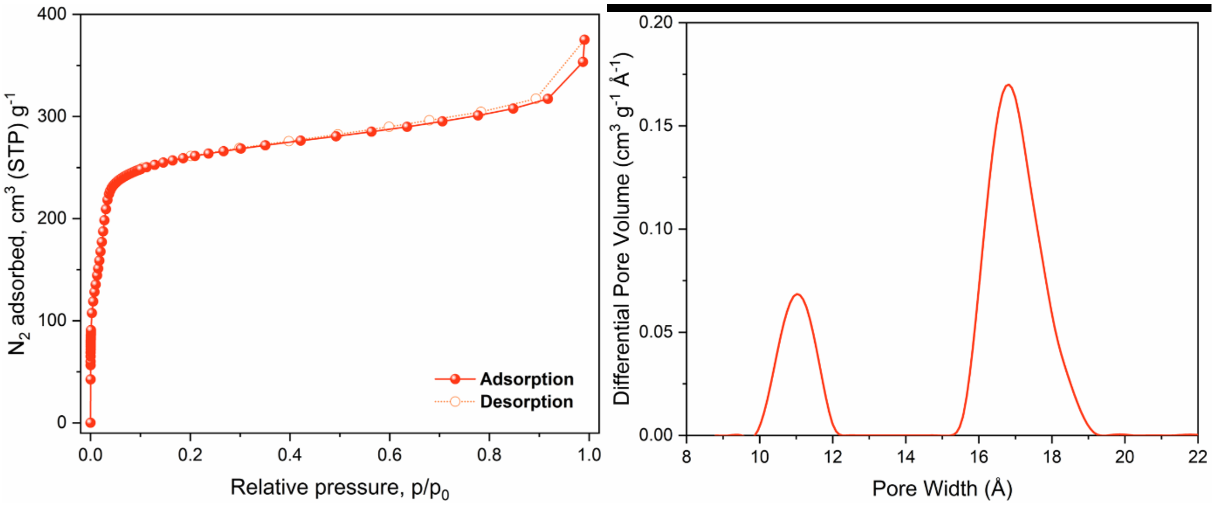

Figure S15. N_2 isotherms (at 77 K) and calculated pore size distribution analysis for as-made Fe-MOF-808.

Supplementary Note 8. Thermogravimetric analysis

For both MOF-808 and Fe-MOF-808 the initial mass loss occurs in the region of 100-130 °C, associated to the loss of any solvent molecules. The frameworks then remain stable up to ~250 °C, where gradual decomposition of organic ligands begins, and by 600 °C only metal oxide remains as the final residue. For Fe-MOF-808 this residue is expected to be a mixture of ZrO_2 and Fe_2O_3, in excellent agreement with the calculated results as seen in Table S3.

Table S4. Calculated and experimental mass losses for MOF-808 and Fe-MOF-808, corresponding to respective framework decomposition down to ZrO_2 and ZrO_2/Fe_2O_3.

MOF material	Calculated mass loss (%)	Experimental mass loss (%)
MOF-808	44.69	44.63
Fe-MOF-808	32.38	31.89

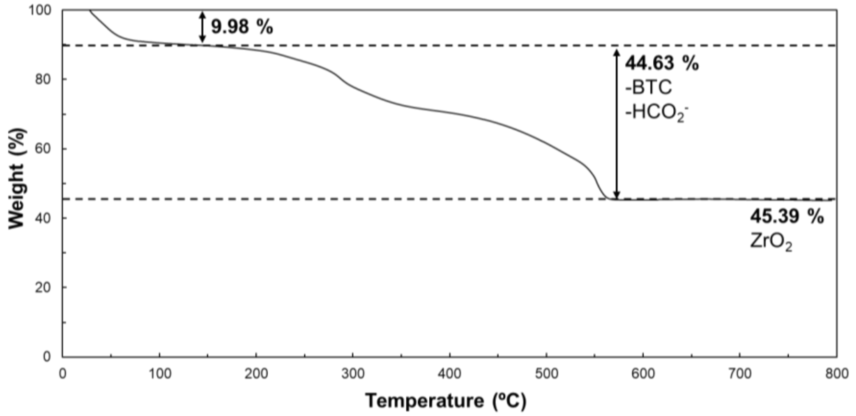

Figure S16. TGA of pristine MOF-808.

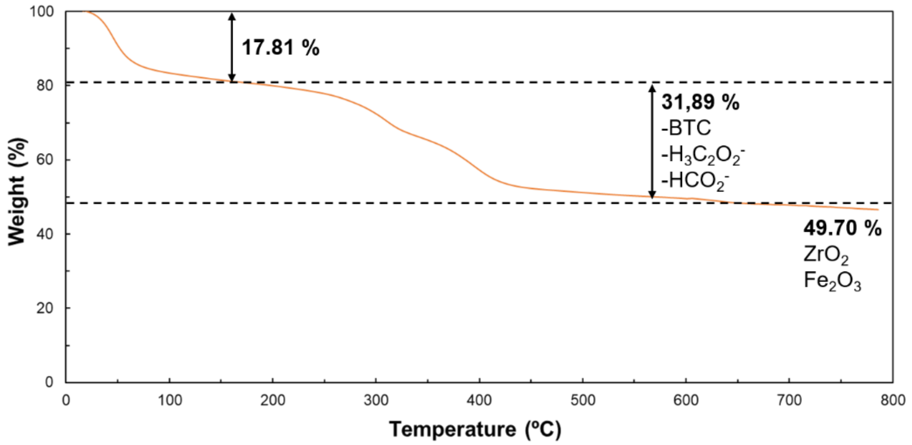

Figure S17. TGA of Fe-MOF-808.

Supplementary Note 9. X-Ray Pair Distribution Function analysis

Synchrotron X-ray total scattering data suitable for pair distribution function (PDF) analysis were acquired at the European Synchrotron Radiation Facility (ESRF), France (beamline ID15A, beamtime MA-5852) using 90 keV (0.13776 Å) X-rays. Samples were first ground into fine

powder, then loaded into kapton capillaries (1.1 mm Ø) and sealed. Data scans were collected for 1 minute. Empty capillary and background total scattering data were also collected for in data processing. To generate PDFs, sample data were processed using the PDFgetX3 programme[7] within the xPDFsuite software package, with a Q_{max} value of 22 $Å^{-1}$. Additional data was collected at Diamond Light Source, UK (beamline I15-1, beamtimes CY34409-1 and CY37864-1) using 76.6 keV (0.1616 Å) X-rays. Samples were first ground into fine powder, then loaded into glass or kapton capillaries (1 mm Ø) and sealed. Data scans were collected for 10 minutes. Empty capillary and background total scattering data were also collected for in data processing. Sample data were processed using PDFgetX3 to a Qmax of 22 $Å^{-1}$.

In all cases, differential PDFs were obtained by subtraction of PDF profiles in real space after applying a normalization factor.

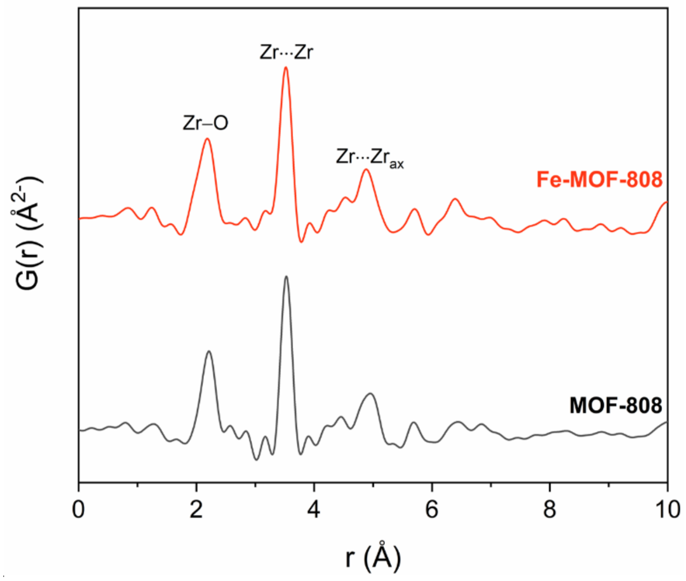

Figure S18. Total PDF data of the as-synthesized materials of this study.

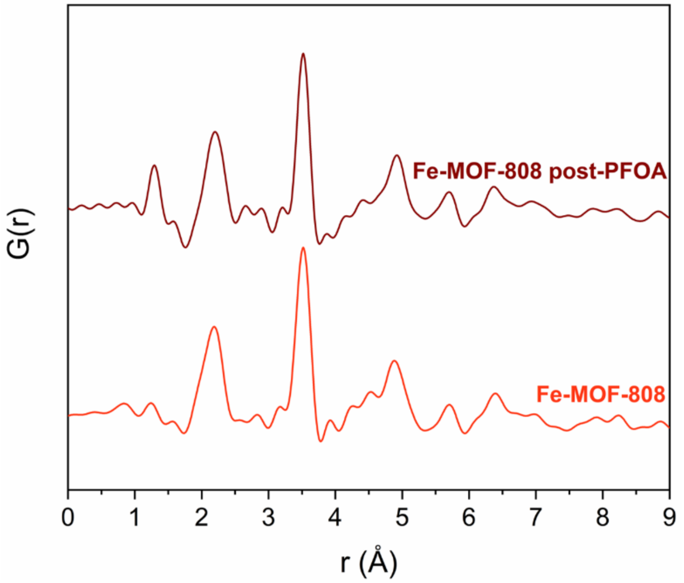

Figure S19. Total PDF data of Fe-MOF-808 before and after PFOA capture.

Supplementary Note 10. X-Ray Absorption Spectroscopy analysis

Preliminary *ex situ* XAS data were collected at Diamond Light Source, UK (beamline B18, beamtime SP35948-1). Main X-ray absorption spectroscopy (XAS) measurements have been collected at NOTOS beamline of the ALBA Synchrotron Light Source facility (Cerdanyola del Vallès, Barcelona, Spain). The synchrotron light coming from a bending magnet has been first vertically collimated, then monochromatized using two pairs of water cooled Si(111) crystals and finally focused on the sample position down to ~800×500 μm2. Si and Rh stripe coatings of the two mirrors were opportunely chosen to guarantee the higher harmonics rejection.

Samples (MOFs or reference systems) were finely grinded and mixed with cellulose to prepare suitable pellets for data collection. Sample/cellulose ratios were estimated for each system considering the targeted absorption edge and the metal content in each solid (as previously determined by chemical analyses).

Fe foil and other Fe standards were measured in transmission mode using Oken ionization chambers, filled with the appropriate mixture of gases. Energy scale have been calibrated at Fe foil edge (7112eV).

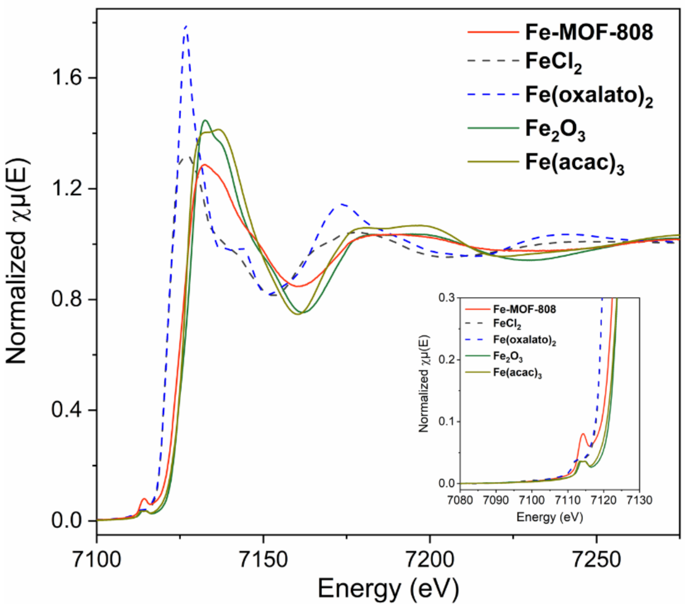

Figure S20. XANES spectra (Fe *K*-edge) of Fe-MOF-808 and reference Fe^{2+} and Fe^{3+} compounds.

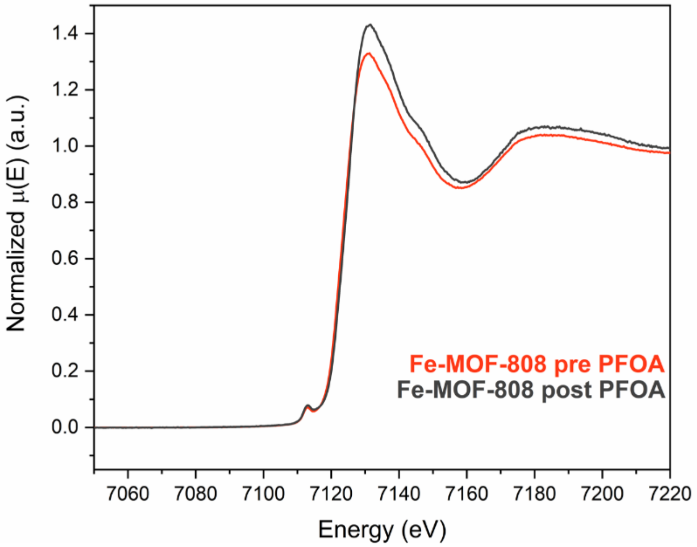

Figure S21. *Ex situ* XANES spectra comparison (Fe *K*-edge) of Fe-MOF-808 batches, before and after capture of PFOA.

In situ XAS measurements were realized by exposing a Fe-MOF-808 pellet to the aqueous solution of PFOA (100 ppm, 0-1000 mL) by using a custom design 3D printed cell (seen in Figure S21), allowing for both transmission and fluorescence measurement. The solution was then allowed to pass through the cell through a constant flow rate of 10 mL/min and XAS data were periodically collected after specific volume amounts. In order to guarantee the interaction solid/liquid particular attention has been paid to the position of inlet and outlet of the liquid circuit with respect the pellet itself. Due to the high absorption of the PFOA solution, spectra at Fe k edge have been collected in fluorescence mode using a 13-element Si detector, from Canberra.

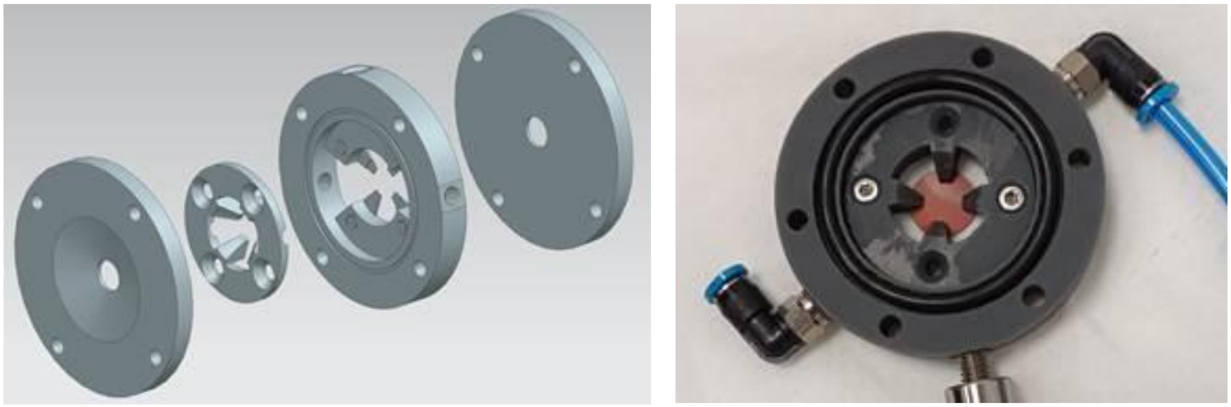

Figure S22. Homemade cell built at BL-16 NOTOS for the performed in situ experiments. The holder contains a pellet sample of Fe-MOF-808.

The raw spectra have been processed according to standard methods, using the Demeter XAS suite.[8] They have been normalized by subtracting pre-edge and post edge line fitted as low-order polynomial curves. The corresponding EXAFS signal has been then extracted in the range 3-12 $Å^{-1}$, k-squared weighted, and Fourier transformed (FT). Pristine Fe-MOF-808 has been used as starting point for all EXAFS modelling, providing the theoretical phases and amplitudes of the scattering paths by means of self-consistent ab-initio calculations performed with FEFF code.[9]
Four shelves were considered for fitting the data (corresponding to $Fe-O_6$ octahedron, Fe-C and Fe-Fe and Fe-Zr contributions). As fitting parameters, we consider α, β, γ, γ1 radial expansion factors for the three different shelves:

$$R_{Fe\text{-}O} = R_{model}\ (1+\alpha)$$

$$R_{Fe\text{-}C} = R_{model}\ (1+\beta)$$

$$R_{Fe\text{-}Fe} = R_{model}\ (1+\gamma)$$

$$R_{Fe\text{-}Zr} = R_{model}\ (1+\gamma1)$$

and four disorder parameters. Coordination numbers of the model have been kept constant. In the following, Figure S23 and Table S4 show the best fitting curve and best fitting parameters:

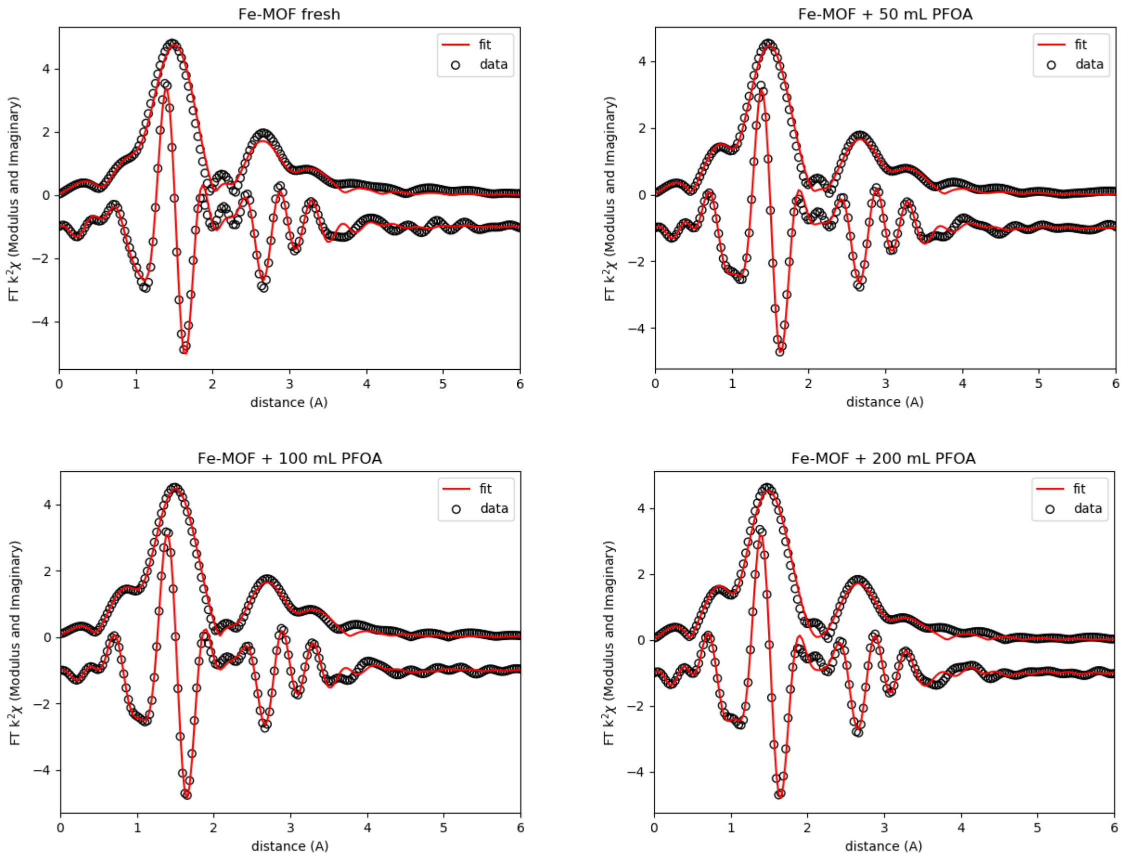

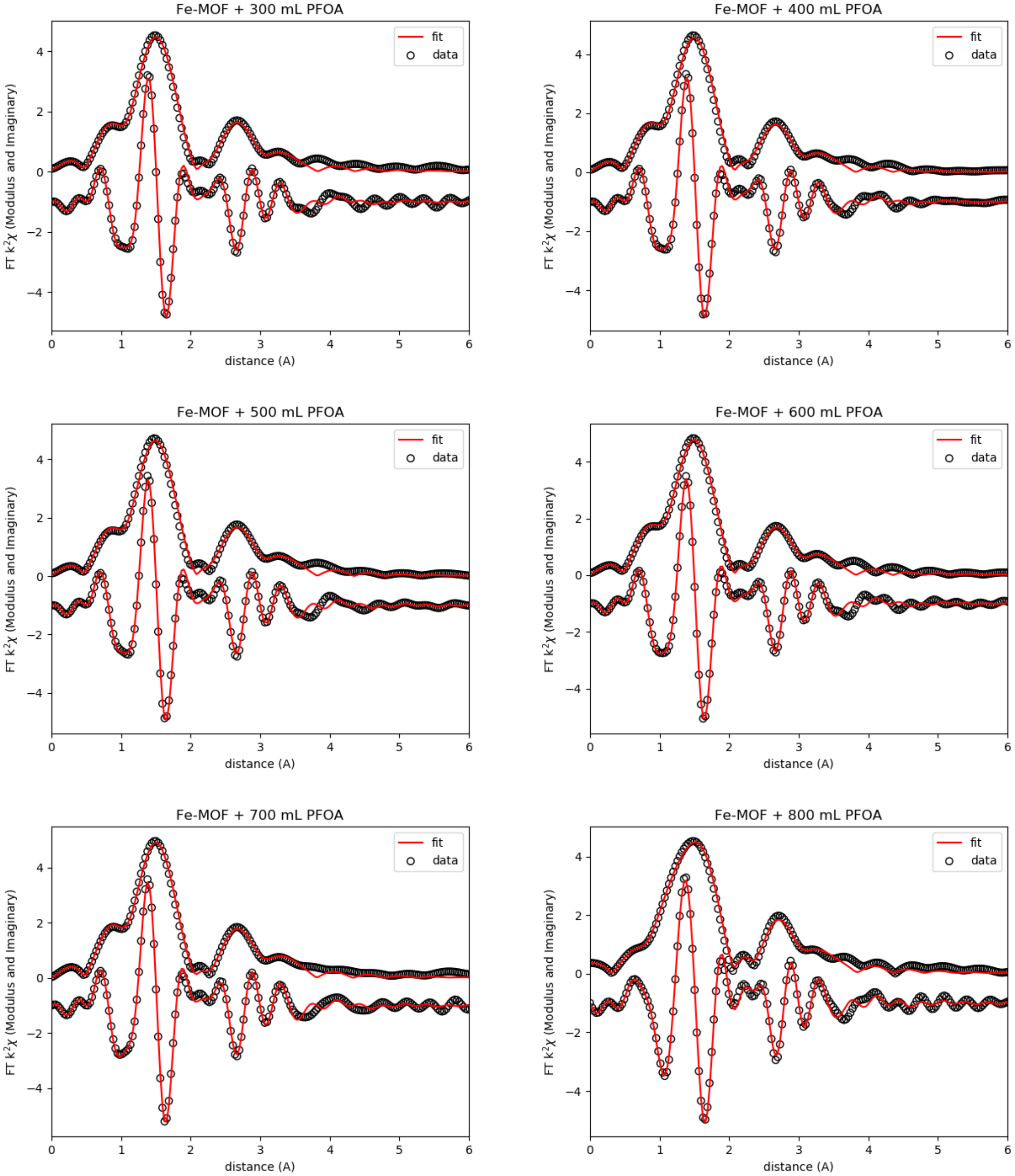

Fe-MOF + 300 mL PFOA
Fe-MOF + 400 mL PFOA
Fe-MOF + 500 mL PFOA
Fe-MOF + 600 mL PFOA
Fe-MOF + 700 mL PFOA
Fe-MOF + 800 mL PFOA
fit
data
FT k²χ (Modulus and Imaginary)
distance (A)

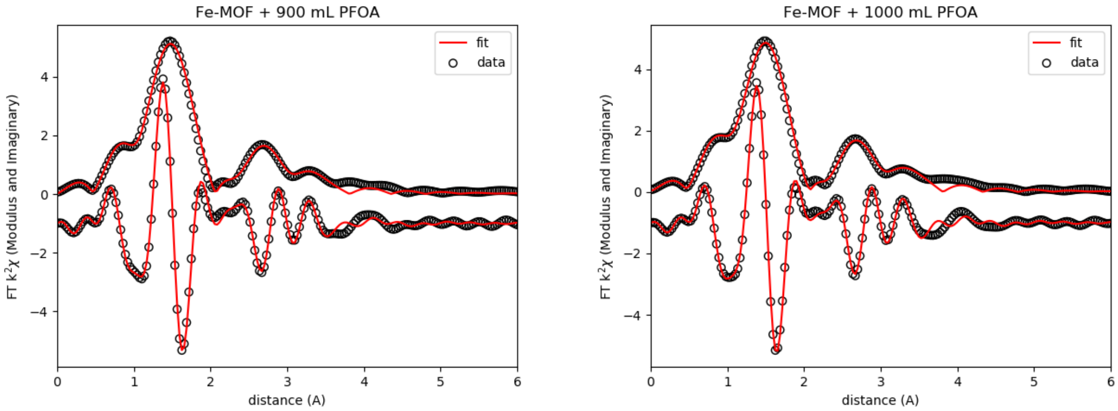

Figure S23. k^2-weighted χ(r) and Img [χ(r)] Fe EXAFS spectra of Fe-MOF-808, after addition of specific volumes of a 100 ppm aqueous PFOA solution, along with the fit calculated from the proposed DFT model.

Table S5. The corresponding fitting parameters for all related Fe-MOF-808 samples.

Sample	α		β		γ		γ1	
	value	error	value	error	value	error	value	error
0 ml PFOA	0.003	0.002	-0.039	0.008	-0.062	0.002	0.079	0.000
50 mL PFOA	0.007	0.010	-0.033	0.041	-0.053	0.008	0.082	0.001
100 mL PFOA	0.013	0.006	-0.026	0.025	-0.051	0.005	0.088	0.001
200 mL PFOA	0.012	0.011	-0.033	0.050	-0.052	0.008	0.084	0.001
300 mL PFOA	0.014	0.008	-0.030	0.035	-0.052	0.007	0.085	0.001
400 mL PFOA	0.012	0.009	-0.030	0.040	-0.052	0.008	0.084	0.001
500 mL PFOA	0.011	0.009	-0.032	0.040	-0.052	0.007	0.086	0.001
600 mL PFOA	0.011	0.007	-0.030	0.030	-0.049	0.005	0.086	0.001
700 mL PFOA	0.012	0.006	-0.029	0.023	-0.048	0.004	0.094	0.000
800 mL PFOA	0.011	0.006	-0.025	0.024	-0.048	0.004	0.094	0.000
900 mL PFOA	0.006	0.009	-0.033	0.035	-0.053	0.008	0.085	0.001
1000 mL PFOA	0.012	0.006	-0.030	0.023	-0.049	0.004	0.094	0.000

Sample	s^2 (O)		s^2 (C)		s^2 (Fe)		s^2 (Zr)	
	value	error	value	error	value	error	value	error
0 ml PFOA	0.006	0.002	0.006	0.004	0.013	0.006	0.007	0.010
50 mL PFOA	0.007	0.005	0.007	0.003	0.012	0.026	0.009	0.076
100 mL PFOA	0.007	0.004	0.007	0.012	0.012	0.016	0.008	0.039
200 mL PFOA	0.007	0.006	0.007	0.003	0.011	0.031	0.011	0.014

300 mL PFOA	0.007	0.004	0.007	0.004	0.012	0.023	0.011	0.090
400 mL PFOA	0.007	0.005	0.007	0.006	0.012	0.029	0.011	0.011
500 mL PFOA	0.007	0.005	0.007	0.006	0.011	0.027	0.011	0.012
600 mL PFOA	0.007	0.004	0.007	0.004	0.012	0.024	0.012	0.018
700 mL PFOA	0.007	0.004	0.007	0.003	0.010	0.016	0.014	0.083
800 mL PFOA	0.006	0.004	0.006	0.006	0.011	0.018	0.012	0.077
900 mL PFOA	0.006	0.004	0.006	0.008	0.012	0.028	0.009	0.085
1000 mL PFOA	0.007	0.004	0.007	0.006	0.011	0.017	0.015	0.084

Supplementary Note 11. Theoretical Calculations

A multi-scale computational methodology is applied in order to elucidate the structural characteristics of the binuclear iron species incorporated in the MOF-808, and their structural, energetics and thermodynamic properties towards the capture of PFOA. Initially, Density Functional Theory (DFT) calculations will be used to elucidate the structure of the binuclear iron species, and subsequently a combination of DFT, Molecular Dynamics (MDs) and Grand Canonical Monte Carlo (GCMC) simulations to give insights into the interaction of PFOA with the Fe-MOF-808.

Models

Two distinct models were employed for MOF-808 and its functionalized analogue Fe-MOF-808: i) a cluster and ii) a periodic model. The cluster model used for DFT calculations of pristine MOF-808 consists of two hydroxo/aquo-terminated Zr_6O_8 clusters linked by benzene-1,3,5-tricarboxylate (BTC) ligands, as reported in our previous works.[3, 10] The periodic model used in the GCMC and MD simulations is taken from previously reported MOF-808 structures, which contain aqua (–OH_2) and hydroxo (–OH) ligands.[11] The periodic Fe-MOF-808 model is constructed by inserting one $Fe_2(\mu_2\text{-OH})_2$ moiety on top of the terminal Zr-(OH) and Zr-(OH_2) groups, removing one proton from the aqua ligands, and adjusting the distances and angles between the oxygen atom of the Zr-OH group and the iron atom of the $Fe_2(\mu_2\text{-OH})_2$ as close as to the optimized DFT geometry. It should be noted that this Fe-MOF-808 model cell used in the GCMC simulations includes three different cases with one, two and four Fe_2 sites per pore (i.e.,

0.5, 1 and 2 Fe per Zr_6 core). In the MD simulations the Fe-MOF-808 model cell includes one Fe_2 site per pore (i.e., 0.5 Fe per Zr_6 core). Although these cases represent a lower iron incorporation than that observed experimentally, the model still provides valuable insights into the enhancement of PFOA uptake.

i) Details for the DFT calculations: The geometries of all cluster models are optimized using the Gaussian 16 program, revision C1.[12] The M06-L density functional[13] is employed in conjunction with the Stuttgart-Dresden (SDD) pseudopotentials[14] and their corresponding basis sets for Zr atoms, while the 6-31G(d,p) basis set[15] is used for all other atoms. In the frequency calculations, MOF framework atoms are treated as inactive, while only the ligand atoms belonging to PFOA, OH^-, H_2O and CH_3COO^- that participate in the replacement reactions reported in Section v, are considered active. During all geometry optimisations, some restrictions have to be applied in order to mimic the crystal environment. Two (2) of the zirconium atoms at the edges of the molecular cluster, and twenty-four (24) oxygen atoms that belong to the BTC ligands are kept frozen. The optimized structures are confirmed as true minima based on frequency analysis, as no imaginary frequencies were detected. For the isolated molecules (PFOA, H_2O, CH_3COOH), a final electronic energy calculation was performed in the liquid phase, with water as the solvent, modelled using the implicit universal solvation model (SMD).[16] Free energy calculations are computed assuming conditions of T = 298.15 K and P = 1 atm, and applying the quasi rigid-rotor oscillator approximation to the vibrational partition functions.[17] Gibbs free energies are computed using the Shermo program.[18] The binding free energies ΔG are calculated in kJ/mol by means of Density Functional Theory (DFT) with the following equation taking into account the concentrations of the molecular species:

$$\Delta G = G_{aq}^{0}[PFOA_xMOF808\,model] + zG_{aq}^{0}[H_2O] + yG_{aq}^{0}[CH_3COOH] - G_{aq}^{0}[MOF808\,model] - xG_{aq}^{0}[PFOA] + zRTln\left(C_{H_2O}\right) + yRTln\left(C_{CH_3COOH}\right) - xRTln(C_{PFOA})$$

where $MOF808$ represents the MOF-808 and Fe-MOF-808 cluster models that were explained above, $PFOA_xMOF808$ represents the MOF-808 or Fe-MOF-808 cluster models interacting with x PFOA molecules and G is the corresponding calculated free energies from the optimised structures. The symbols z and y represent the amount of H_2O and/or CH_3COOH molecules that

are released as products, and the terms RTln(C) in the equation correspond to the free energy change of 1 mol of H_2O, CH_3COOH and PFOA from 1 bar pressure in the gas phase to a solution of 1M concentration. For example, the term $zRTln(C_{H_2O})$ in the equation corresponds to the free-energy change of z moles of gaseous H_2O from the liquid state (where water has concentration of 55.34 M) to a solution of 1 M. The concentration of both PFOA and CH_3COOH is 0.00025M.

The analysis of the interactions of PFOA with the Fe-MOF-808 models is performed by using the Interaction Region Indicator (IRI) method,[19] as implemented within the Multiwfn 3.8(dev) wavefunction analysis code.[20]

ii) Details for the GCMC calculations: Parameters for the GCMC simulations are taken from our previous work.[1] The van der Waals non-bonding interactions are described using the Lennard–Jones (LJ) potential, while electrostatic interactions are modelled using the Coulomb potential, as described by the following equation:

$$V_{ij} = 4\varepsilon_{ij}\left[\left(\frac{\sigma}{r_{ij}}\right)^{12} - \left(\frac{\sigma}{r_{ij}}\right)^{6}\right] + \frac{1}{4\pi\varepsilon_0}\frac{q_i q_j}{r_{ij}}$$

where V is the total energy of the interacting atoms i and j, r_{ij} is their distance, σ_{ij} is their equilibration distance, ε_{ij} is the well depth, q_i and q_j are the partial charges of the interacting atoms, and ε_0 is the dielectric constant in vacuum. Lennard–Jones (LJ) parameters are adopted from the Universal Force Field (UFF),[21] while cross-interactions are determined using Lorentz–Berthelot mixing rules. Partial atomic charges for MOF atoms are obtained from calculations with the PACMAN code.[22] For electrostatic and van der Waals interactions between PFOA molecules and the host materials, force field parameters from a study by Erkal et al. are employed.[23] The van der Waals interactions are modelled using a Lennard–Jones potential with a cut-off distance of 10.0 Å, without the inclusion of tail corrections. Electrostatic interactions are treated using the Ewald summation method to account for long-range effects.

All Grand Canonical Monte Carlo (GCMC) simulations are performed using the RASPA molecular simulation software.[24] A periodic simulation box consisting of a 1 × 1 × 1 unit cell was constructed, ensuring that its dimensions in each direction are at least twice the cut-off distance. For equilibration over 10^6 cycles are applied, followed by an additional 10^7 cycles for each thermodynamic state in order to compute the desired system properties. Each cycle comprises N

trial moves, where N corresponds to the number of guest molecules present in the simulation box at that moment, or was set to 20, if fewer than 20 molecules were present. Trial moves include creation, deletion, translation, and rotation of particles, each considered with equal probability. Guest molecules are allowed to rotate and translate, while the MOF framework atoms are remained fixed throughout the simulations.

iii) Details for the MD simulations: The Molecular Dynamics simulations employ a force field incorporating both bonded and non-bonded interactions. For the PFOA, the CF_3 and CF_2 units are modelled as united atoms, whereas the atoms on the carboxyl group are treated explicitly. Force field parameters for the PFOA are taken from a previous MD study of PFOA capture with MOFs.[23] The van der Waals interactions are described using the Lennard–Jones (LJ) potential, while electrostatic interactions are modelled using the Coulomb potential, consistent with the Grand Canonical Monte Carlo (GCMC) simulations. Bonded interactions are described using harmonic potentials for bond stretching and dihedral angles, while angle interactions with a hybrid Fourier and cosine/periodic potential. Improper dihedral interactions are modelled with a Fourier potential. LJ parameters, along with all bonded interaction parameters, are obtained from the Universal Force Field (UFF), while cross-interactions are determined using Lorentz–Berthelot mixing rules. For water molecules, the TIP4P model is used.[25] Partial atomic charges for MOF atoms are derived from PACMAN calculations. For the van der Waals interactions, a cut-off distance of 12.0 Å is applied to the Lennard–Jones potentials without incorporating tail corrections. Electrostatic interactions are computed using the particle-particle particle-mesh (PPPM) solver method.

All MD simulations are conducted using the LAMMPS molecular simulation software.[26] A periodic simulation box consisting of a 2 × 2 × 2 supercell is constructed, ensuring that its dimensions in each direction are at least twice the cut-off distance. A series of systems were constructed to investigate the interactions between PFOA molecules and the MOF frameworks. For both MOF-808 and Fe–MOF-808, three configurations were prepared containing 1, 2, and 4 PFOA molecules per pore, respectively. This variation in loading was chosen to assess how different PFOA concentrations influence the adsorption behavior and interaction mechanisms as well as the positioning of molecules inside the pore with increasing uptake. Additionally, one system was generated for each MOF type containing 1 PFOA molecule and 4 water molecules per

pore. These mixed systems enable the evaluation of competitive adsorption between water and PFOA, as well as the effects of water on the PFOA–MOF interaction dynamics in comparison with the "dry" systems.

The PFOA and water molecules are randomly introduced into the simulation box at the start of the simulation. Following an initial geometric minimization, an annealing process is carried out using the NVT ensemble. A timestep of 1 fs is maintained throughout the simulation. The system is gradually heated over 0.5 ns to 1000 K, where it remains for an additional 0.5 ns, with the MOF structure held fixed to preserve its integrity. Subsequently, the system is cooled to 300 K over 0.5 ns and equilibrated at room temperature for an additional 1 ns under NVT conditions, during which both the MOF and PFOA molecules are allowed to move freely. This annealing procedure ensures a well-equilibrated system, with PFOA molecules randomly distributed within the pores of the MOF.

In the final stage, the algorithm is switched to the NPT ensemble at room temperature (300 K) and 1 atm, allowing both atomic positions and the simulation box dimensions to fluctuate. The total NPT simulation is 100 ns, with statistical analysis based on data collected from the final 50 ns of the trajectory to ensure the system is well equilibrated before extracting conclusions.

DFT Results

iv) Structural elucidation of Fe-MOF-808: Initially, we investigated the structural and energetic characteristics of the deposition of two Fe(III) atoms on the nodes of the MOF-808. Three different models were constructed by considering different binding modes of the acetate and hydroxide ligands to the iron bi-metallic core: i) each acetate binds in a bidentate manner to an iron atom and the hydroxides are bridged between the two iron atoms, ii) one acetate binds in a monodentate manner to an iron atom and the hydroxides are bridged between the two iron atoms, and iii) each acetate binds via bidentate manner to two iron atom, while the hydroxide anions binds as terminal ligands to the iron atoms. To reduce the complexity of the system, due to many possible combinations to couple the unpaired electrons of the Fe(III)/Fe(III) atoms, we decided to study only the deposition of the high-spin ferromagnetically coupled Fe(II)-Fe(II) pairs with a total spin multiplicity of 11. The optimized geometries are shown in Figure S24.

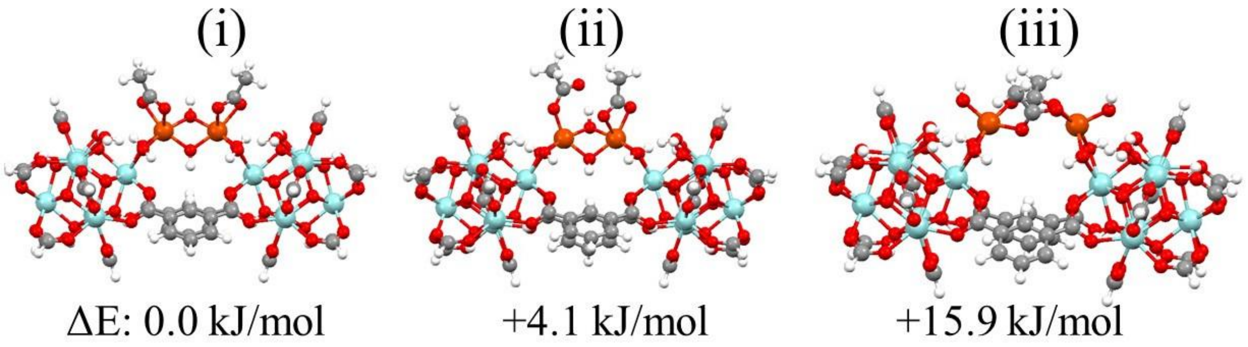

Figure S24. Optimized geometries of three different $Fe_2(hydroxo)_2(acetate)_2$ models deposited on the MOF-808 model. Their relative energy differences (in kJ/mol) are also shown.

Model (i) is the most stable with the other two being energetically higher by 4.1 and 15.9 kJ/mol, and with the important structural properties (Fe···Fe, Fe···Zr and Fe-O distances) being significantly different than the experimentally observed ones.

v) Thermodynamics for the PFOA adsorption: Subsequently, the thermodynamics of all possible reactions were investigated for pristine MOF-808 and Fe-MOF-808 using the M06-L density functional with the SDD pseudopotentials and 6-31G(d,p) basis set. The first case of reactions involves the substitution of a hydroxo ligand in MOF-808 by a perfluorooctanoic acid (PFOA) molecule, forming (PFOA)-MOF-808, where the PFOA molecules are bound in a monodentate and bidentate mode respectively. The second reaction entails the replacement of two hydroxo ligands with two PFOA molecules, and the replacement of two hydroxo and aqua ligands, forming $(PFOA)_2$-MOF-808, where the PFOA molecules are bound in a monodentate and bidentate mode respectively. The corresponding reaction equations are:

(1a) MOF-808 + PFOA → (PFOA)-MOF-808 + H_2O (monodentate)

(1b) MOF-808 + PFOA → (PFOA)-MOF-808 + H_2O (bidentate)

(2a) MOF-808 + 2PFOA → $(PFOA)_2$-MOF-808 + $2H_2O$ (monodentate)

(2b) MOF-808 + 2PFOA → $(PFOA)_2$-MOF-808 + $2H_2O$ (bidentate)

The calculated free energies for these reactions are -103.8 (1a), -90.2 (1b), -151.6 (2a) and -164.6 (2b) kJ/mol, respectively, indicating a thermodynamically favourable process. Notably, the results

suggest a strong and favourable interaction between PFOA molecules and the Zr_6O_8 clusters, as reflected in the increasingly negative reaction energies. Moreover, the two binding modes; mono- versus bi-dentate, are close in energy. While enthalpies favour the monodentate binding mode, entropic contributions from the release of an additional water molecule favour the bidentate mode. Subsequently, similar calculations were performed to investigate the reactions involving the iron and zirconium sites of the Fe-MOF-808 and the substitution of its ligands with PFOA molecules. This analysis aimed to elucidate the contributions of the iron and zirconium centers to the PFOA adsorption. The first reaction entails the substitution of an acetate ligand in the Fe_2-hydroxo cluster of Fe-MOF-808 with a perfluorooctanoic acid (PFOA) molecule, forming Fe(PFOA)-MOF-808. In the second reaction, both acetate ligands are replaced by two PFOA molecules, forming $Fe(PFOA)_2$-MOF-808. A third possibility is considered, where water displaces PFOA from the iron site. The corresponding reaction equations are:

(3a) Fe-MOF-808 + PFOA → Fe(PFOA)-MOF-808 + CH_3COOH

(3b) Fe-MOF-808 + 2PFOA → $Fe(PFOA)_2$-MOF-808 + $2CH_3COOH$

(3c) Fe(PFOA)-MOF-808 + H_2O → Fe(OH)-MOF-808 + PFOA

The calculated free energies for these reactions are -35.7 , -81.8 and +66.1 kJ/mol, respectively, indicating a less favourable (less negative) interaction between PFOA molecules and the iron sites in the Fe-MOF-808 compared to the pristine MOF-808 system. Moreover, the displacement of PFOA by water from the iron site is not predicted to be favourable, since the free energy change is positive (+66.1 kJ/mol).

The next step involved investigating the substitution reactions, in which a PFOA molecule replaces a hydroxo ligand of the Zr_6O_8 cluster in Fe-MOF-808, forming (PFOA)-Fe-MOF-808. A similar reaction was examined in which two PFOA molecules replace two hydroxo ligands of the Zr_6O_8 node, resulting in the formation of $(PFOA)_2$-Fe-MOF-808 bound in a monodentate manner. The corresponding reaction equations are:

(4) Fe-MOF-808 + PFOA → (PFOA)-Fe-MOF-808 + H_2O

(5) Fe-MOF-808 + 2PFOA → $(PFOA)_2$-Fe-MOF-808 + $2H_2O$

The calculated free energies for these reactions are -89.3 kJ/mol and -189.9 kJ/mol, respectively, indicating a strong and thermodynamically favorable (more negative) interaction between PFOA molecules and the Zr_6O_8 clusters, rather than with the iron sites in Fe-MOF-808. Furthermore, the interactions between PFOA and the Zr_6O_8 cluster are even stronger in Fe-MOF-808 compared to pristine MOF-808. This suggests that the presence of the Fe_2-hydroxo functionalization enhances the binding of PFOA primarily by strengthening weak interactions rather than through direct coordination with PFOA molecules.

To further investigate this interaction, the simultaneous binding of one PFOA molecule to the Zr_6O_8 cluster and another to the Fe_2-hydroxo cluster in Fe-MOF-808 was considered. The binding sites remain same as in previous cases, where a hydroxo ligand is replaced in the Zr_6O_8 cluster and an acetate ligand in the Fe_2-hydroxo cluster. This results in the formation of an alternative configuration of (PFOA)-Fe(PFOA)-MOF-808 compared to the previously studied cases. The corresponding reaction equation is:

(6) Fe-MOF-808 + 2PFOA → PFOA-Fe(PFOA)-MOF-808 + CH_3COOH + H_2O

Two distinct binding configurations were considered: one in which the PFOA molecules attach on opposite sides of the Fe-MOF-808 model and another where they bind on the same side. The calculated free energies for these configurations are -115.2 kJ/mol and -101.5 kJ/mol, respectively. Notably, these interactions exhibit energetics that fall between those observed for pristine MOF-808 and Fe-MOF-808 in their interactions with PFOA molecules.

Finally, the last case is the incorporation of three PFOA molecules into the Fe-MOF-808 system. Given the multiple potential binding sites, two distinct configurations were examined. In the first configuration, two hydroxo groups are replaced by PFOA molecules, which bind near the Zr atoms, while one acetate group of the Fe_2-hydroxo cluster substituted by a third PFOA molecule, which binds to an iron atom. In the second configuration, the replacements are reversed: two acetate groups and one hydroxo group are substituted by three PFOA molecules, such that two PFOAs interact with the iron atoms, and one interacts with a Zr atom. The corresponding reaction stoichiometries are:

(7a) Fe-MOF-808 + 3PFOA → $(PFOA)_2$-Fe(PFOA)-MOF-808 + CH_3COOH + $2H_2O$

(7b) Fe-MOF-808 + 3PFOA → PFOA)-Fe$(PFOA)_2$-MOF-808 + $2CH_3COOH$ + H_2O

The calculated electronic energies for these reactions are -210.2 kJ/mol and -145.7 kJ/mol, respectively. A key observation is the energy difference between the two systems, despite both containing the same number of PFOA molecules. The more favorable (more negative) interaction occurs in the configuration, when two PFOA molecules are bound to the Zr_6O_8 nodes, further confirming the findings from earlier models with one or two PFOA molecules.

All results are summarized in the Table S5 below. The calculated enthalpies and free energies of adsorption indicate that the incorporation of one or two PFOA molecules into both pristine MOF-808 and Fe-MOF-808 is exothermic and exergonic (i.e., spontaneous), as evidenced by the negative energy values in the reactions of the respective models. Focusing on the Fe-MOF-808 models, a clear preference for PFOA interacting with Zr atoms over Fe atoms is observed. This trend is further evident in the models with three PFOA molecules, where the interaction energy for the configuration with two PFOA molecules bound to Zr is approximately ~65 kJ/mol lower (stronger) than the alternative configuration of two PFOA molecules bound to the iron sites. Another key observation is that the zirconium sites of the Fe-MOF-808 exhibit stronger interactions with PFOA molecules compared to pristine MOF-808. The lowest energy for the adsorption of two PFOA molecules at the zirconium sites of a pristine MOF-808 model is -151.6 kJ/mol, whereas the interaction energy for a similar binding configuration of the Fe-MOF-808 model is -194.0 kJ/mol. This suggests that the addition of the Fe_2-hydroxo functional group to pristine MOF-808 enhances the affinity for PFOA binding.

Table S6. Reaction enthalpies and free energies (in kJ/mol) for the adsorption of 1, 2 and 3 PFOA molecules to the pristine MOF-808 and Fe-MOF-808.

	**Reaction Path**	**ΔH (kJ/mol)**	**ΔG (kJ/mol)**
1a	*MOF-808 + PFOA → (PFOA)-MOF-808 + H_2O (monodentate)*	-161.9	-103.8
1b	*MOF-808 + PFOA → (PFOA)-MOF-808 + H_2O (bidentate)*	-111.2	-90.2
2a	*MOF-808 + 2PFOA → $(PFOA)_2$-MOF-808 + 2H_2O (monodentate)*	-260.4	-151.6
2b	*MOF-808 + 2PFOA → $(PFOA)_2$-MOF-808 + 4H_2O (monodentate)*	-204.7	-164.6
3a	*Fe-MOF-808 + PFOA → Fe(PFOA)-MOF-808 + CH_3COOH*	-53.4	-35.7
3b	*Fe-MOF-808 + 2PFOA → $Fe(PFOA)_2$-MOF-808 + 2CH_3COOH*	-112.0	-81.8
3c	*Fe(PFOA)-MOF-808 + H_2O → Fe(OH)-MOF-808 + PFOA*	+125.9	+66.1
4	*Fe-MOF-808 + PFOA → PFOA-Fe-MOF-808 + H_2O*	-148.1	-89.3
5	*Fe-MOF-808 + 2PFOA → $(PFOA)_2$-Fe-MOF-808 + 2H_2O*	-318.4	-189.9
6a	*Fe-MOF-808 + 2PFOA → (PFOA)-Fe(PFOA)-MOF-808 + CH_3COOH + H_2O (opposite side)*	-196.7	-115.2
6b	*Fe-MOF-808 + 2PFOA → 2PFOA-Fe-MOF-808 + CH_3COOH + H_2O (same side)*	-174.4	-101.5
7a	*Fe-MOF-808 + 3PFOA → $(PFOA)_2$-Fe(PFOA)-MOF-808 + CH_3COOH + 2H_2O*	-352.4	-210.2
7b	*Fe-MOF-808 + 3PFOA → (PFOA)-$Fe(PFOA)_2$-MOF-808 + 2CH_3COOH + H_2O*	-247.9	-145.7

For further analysis, the two most thermodynamically favorable configurations with two PFOA molecules adsorbed on the zirconium sites of pristine MOF-808 and Fe-MOF-808, were examined using the Interaction Region Indicator (IRI) method. In the case of pristine MOF-808, the structure from case (2a) (shown in Table S5) with a Gibbs free energy change of ΔG = –151.6 kJ/mol was selected. For Fe-MOF-808, the model from case (5b) with ΔG = –189.9 kJ/mol was chosen. Notably, in both models, the PFOA molecules are coordinated near the Zr nodes in a similar fashion, thereby allowing the specific influence of the Fe_2-hydroxo functional group to be isolated and more clearly assessed in Fe-MOF-808. The nature of the interactions was qualitatively

explored through IRI analysis and excluding covalent bond regions, with the resulting isosurfaces depicted in the Figure S25. The IRI plots reveal regions of van der Waals (vdW) interactions between the PFOA molecules and surrounding framework atoms, represented by green isosurfaces. Regions of stronger, typically covalent, interactions appear as dark blue isosurfaces, whereas repulsive or steric effects are highlighted in red. A significant difference is observed between the two MOF structures: for MOF-808 (part A of Figure S25). The green vdW interaction regions between the PFOA molecules and the framework are relatively sparse. On the contrary, more attractive areas are calculated in the Fe-MOF-808 model (part B of Figure S25). The spatial arrangement of the two PFOA molecules around the Fe_2-hydroxy group generates additional regions of vdW interactions, contributing to the overall stabilization of the system, as supported by the more favourable DFT energy values.

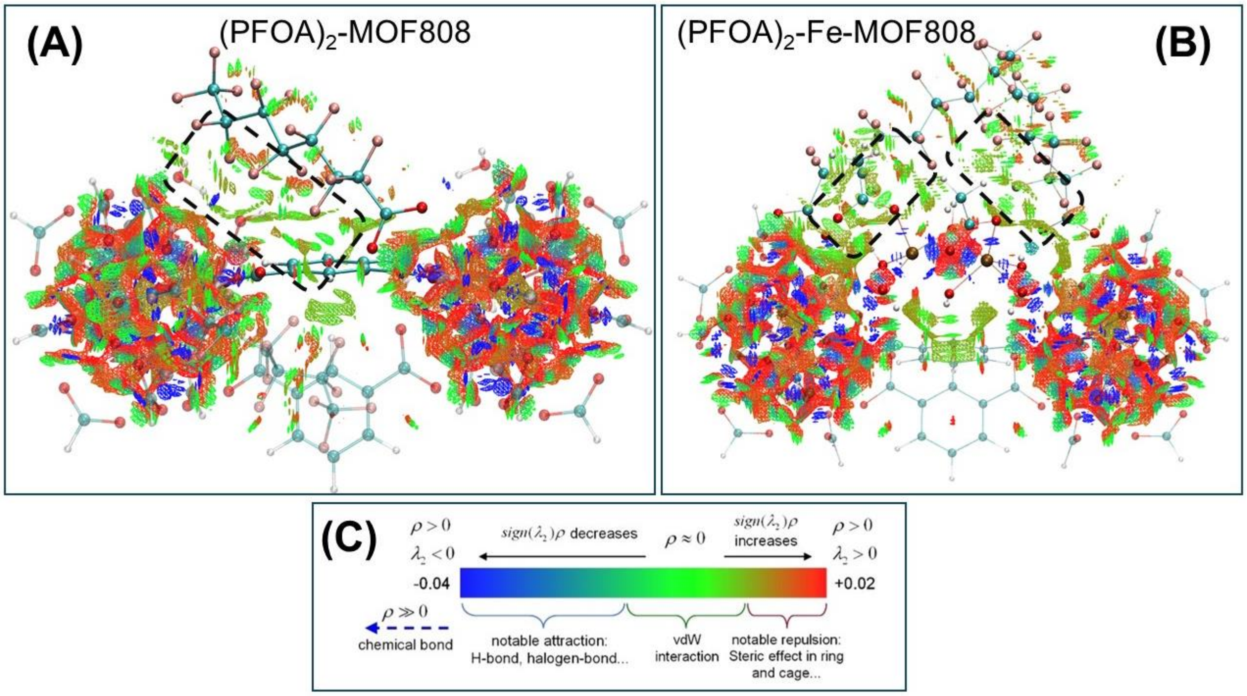

Figure S25. Interaction Region Indicator (IRI) Plots of two PFOA molecules interacting with the zirconium sites of: A) the pristine MOF-808 (left) and B) the Fe-MOF-808 (right). C) Standard colouring method and chemical explanation of the IRI isosurfaces (bottom part). The areas of significant interactions between PFOA and the MOF atoms are emphasized in black dotted rectangles.

vi) GCMC results: The gravimetric uptakes of PFOA in both pristine and modified Fe–MOF-808 systems were determined using GCMC simulations, as presented in Figure S26. As previously described, the Fe–MOF-808 model employed in these simulations comprises three configurations containing one, two, and four Fe_2 sites per pore (corresponding to 0.5, 1, and 2 Fe per Zr_6 core, respectively). Systems with a higher number of Fe_2 sites display increased PFOA adsorption at low pressures (shown at Figure S26 b)), attributable to the stronger interactions between PFOA molecules and the Fe_2 centers, consistent with the DFT results. Conversely, at high pressures, the total uptake decreases due to the reduced free pore volume caused by the additional Fe_2 sites. In the high-loading regime, the PFOA uptake exhibits a clear correlation with the accessible pore volume. It should be mentioned that these GCMC simulations underestimate the real gravimetric capacity, because they don't include the PFOA molecules that are chemisorbed either on the ZrO_2 nodes or on the Fe sites. Therefore, the computed values for the capacity include only PFOA molecules physisorbed in the pores.

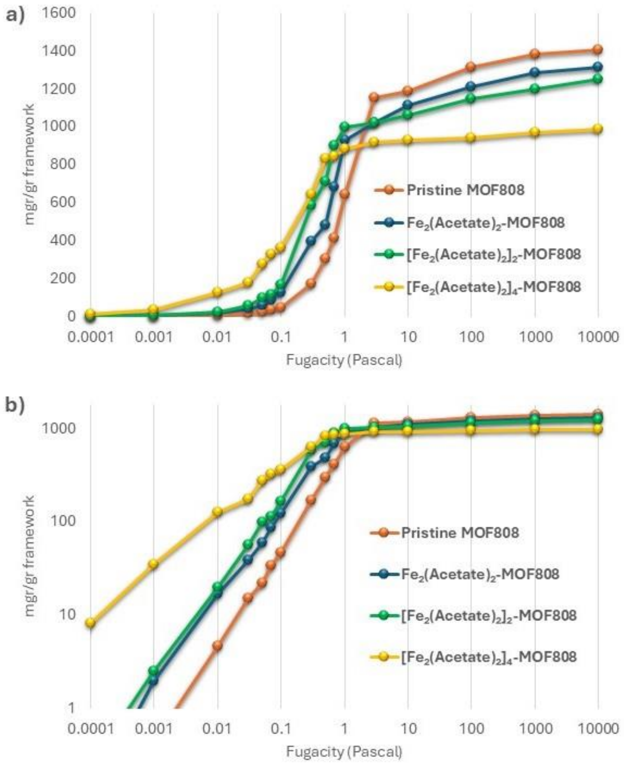

Figure S26. a) Gravimetric uptake of PFOA at 298K b) Log scale of gravimetric uptake of PFOA at 298K, by increasing the iron percentage inside the simulation cell.

vii) MDs results: The main findings from the MD simulations are presented in the Radial Distribution Function (RDF) graphs in in Figures S27-S29. The first set of systems includes one PFOA molecule in the pores of MOF-808 and Fe-MOF-808 respectively (Figure S27, Parts A and B). The radial distribution of different atoms or groups of the PFOA molecule are plotted relative to the zirconium atoms of the pristine MOF-808 and Fe-MOF-808. We have used the oxygen atoms, the terminal -CF_3 group or the center of mass as the reference points of the PFOA molecule. Conclusions are similar regardless the chosen reference point, but the discussion will be focused on the oxygen atoms of the PFOA as the reference point. For pristine MOF-808 (Figure S27, part A), a prominent peak at about 5 Å (shown in dashed lines) marks the optimal interaction distance between the PFOA and the zirconium site for pristine MOF-808. PFOA approaches the zirconium sites of the MOF via its oxygen atoms. The same peak exists for Fe-MOF-808 (Figure S27, part B), however a double peak at 4.5 and 5.1 Å (Figure S27, part C) means that PFOA may also interact with the iron sites.

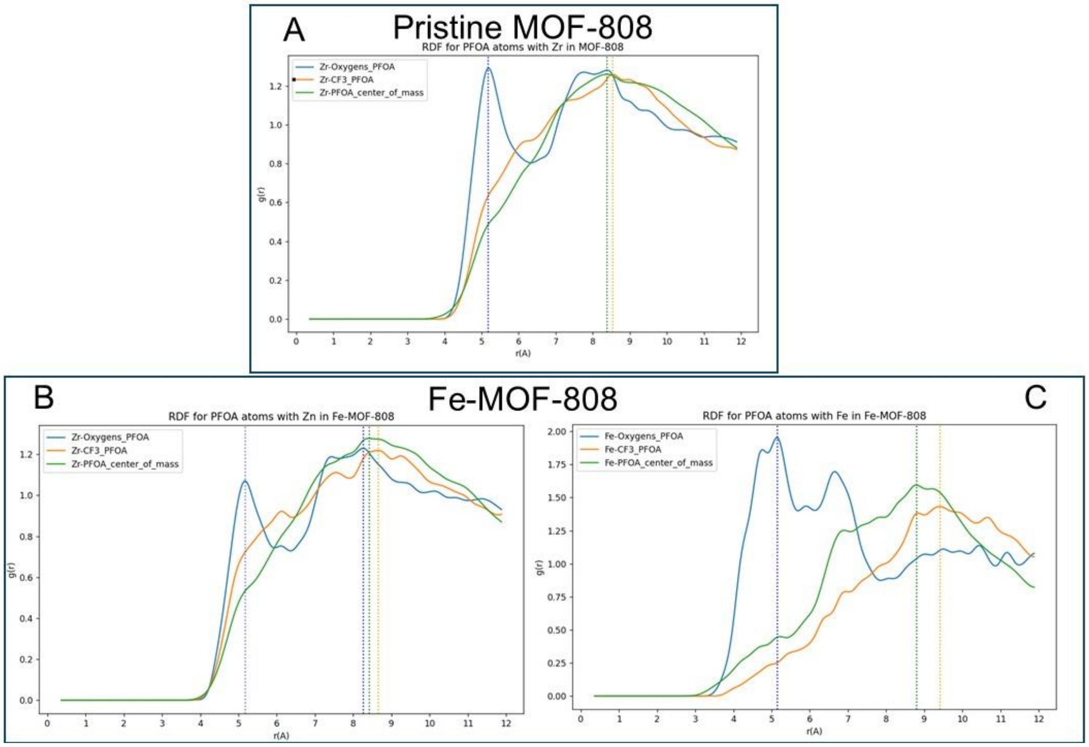

Figure S27. Radial Distribution Functions (RDFs) of PFOA molecule with respect to the A) zirconium atoms in pristine MOF-808, B) zirconium atoms in Fe-MOF-808, and C) iron atoms in the Fe-MOF-808.

By comparing the relative intensities of the oxygen RDF peaks at 5 Å (Figure S27, parts A and B), we can conclude that the intensity of the Zr-$O_{(PFOA)}$ pairs in the Fe-MOF-808 is reduced, which can be explained by the increased number of interaction points in the functionalized pores. PFOA molecules can now interact with zirconium or iron sites.

Subsequently, we studied the insertion of multiple PFOA molecules inside the pores, and the simultaneous insertion of 1 PFOA and 4 H_2O inside the pores of MOF-808 (Figure S28) and Fe-MOF-808 (Figure S29).

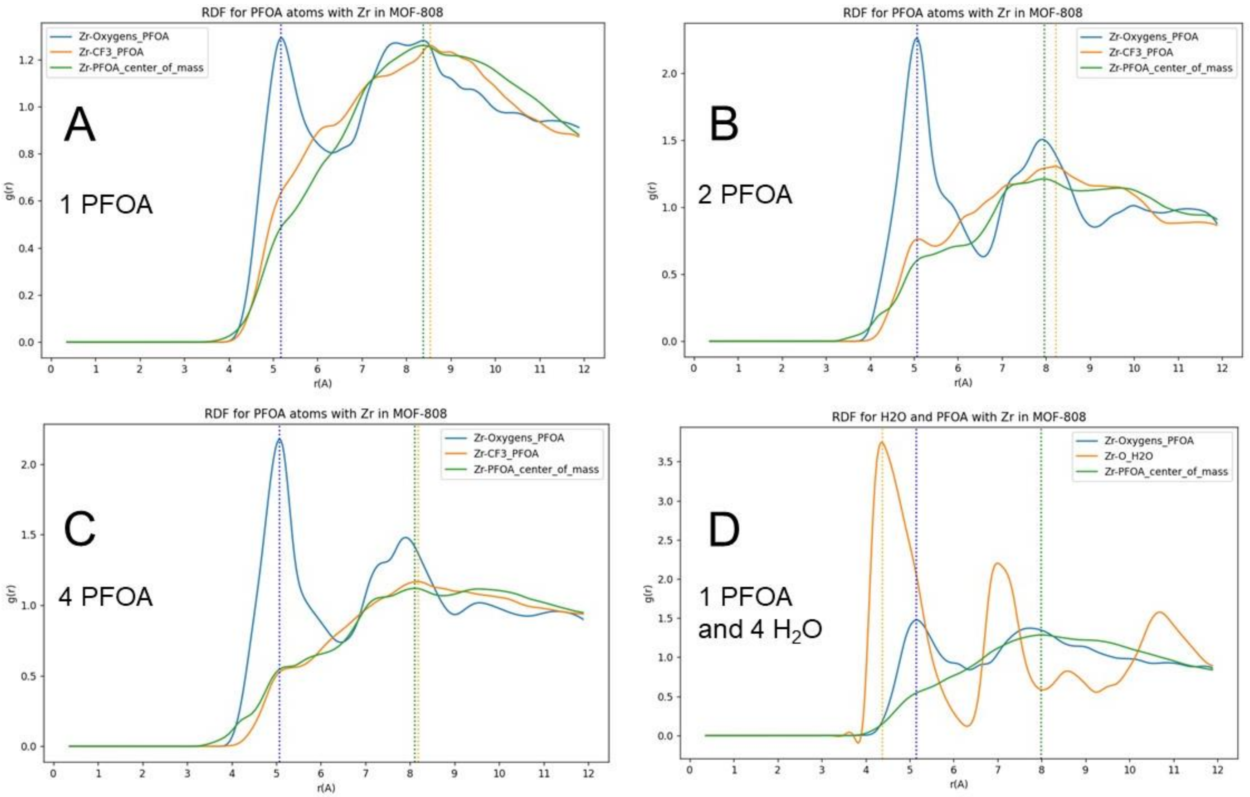

Figure S28. A), B) and C) Radial Distribution Functions (RDFs) of PFOA with respect to the zirconium atoms in pristine MOF-808 with 1, 2 and 4 PFOAs per unit cell respectively, D) RDF of zirconium and oxygen atoms of PFOA and H_2O in pristine MOF-808, with 1 PFOA and 4 water molecules per unit cell.

In Figure S28, panels A–C correspond to the systems containing 1, 2, and 4 PFOA molecules per pore, respectively, without the presence of water. In all three cases, a distinct peak appears at

approximately 5 Å, corresponding to the optimal interaction distance between the oxygen atoms of PFOA and the Zr sites of the framework (blue line). This distance remains unchanged regardless of the PFOA loading, indicating that the PFOA–Zr interaction is sufficiently strong to remain stable even as the pore becomes increasingly occupied. Furthermore, the intensity of this peak increases with higher PFOA loadings (2 and 4 molecules per pore), suggesting that all PFOA molecules preferentially interact with the Zr sites in a similar manner, rather than interacting with other atoms of the pristine MOF-808 structure.

In part D of Figure S28, which represents the system with one PFOA molecule and four water molecules per pore, a similar trend is observed. The characteristic PFOA–Zr interaction peak appears at the same distance as in the corresponding "dry" system (one PFOA, no water, in part A of Figure S28), demonstrating that the presence of water does not disrupt this primary interaction. Water molecules also interact with the open Zr sites, exhibiting a shorter interaction distance of ~4.3 Å and a more intense peak, consistent with their higher concentration in the pore. However, the characteristics of the RDF (blue lines in parts A and D of Figure S28) that correspond to the $Zr\text{-}O_{(PFOA)}$ pair, remain nearly identical. This indicates that the presence of the water molecules does not significantly alter the PFOA binding inside the MOF-808.

Similar analysis is also performed for the Fe-MOF-808. The RDF graphs for the systems that contain multiple PFOAs (parts A, B and C of Figure S29) and 1 PFOA with 4 H_2O (part D of Figure S29) in the pore of Fe-MOF-808 are presented.

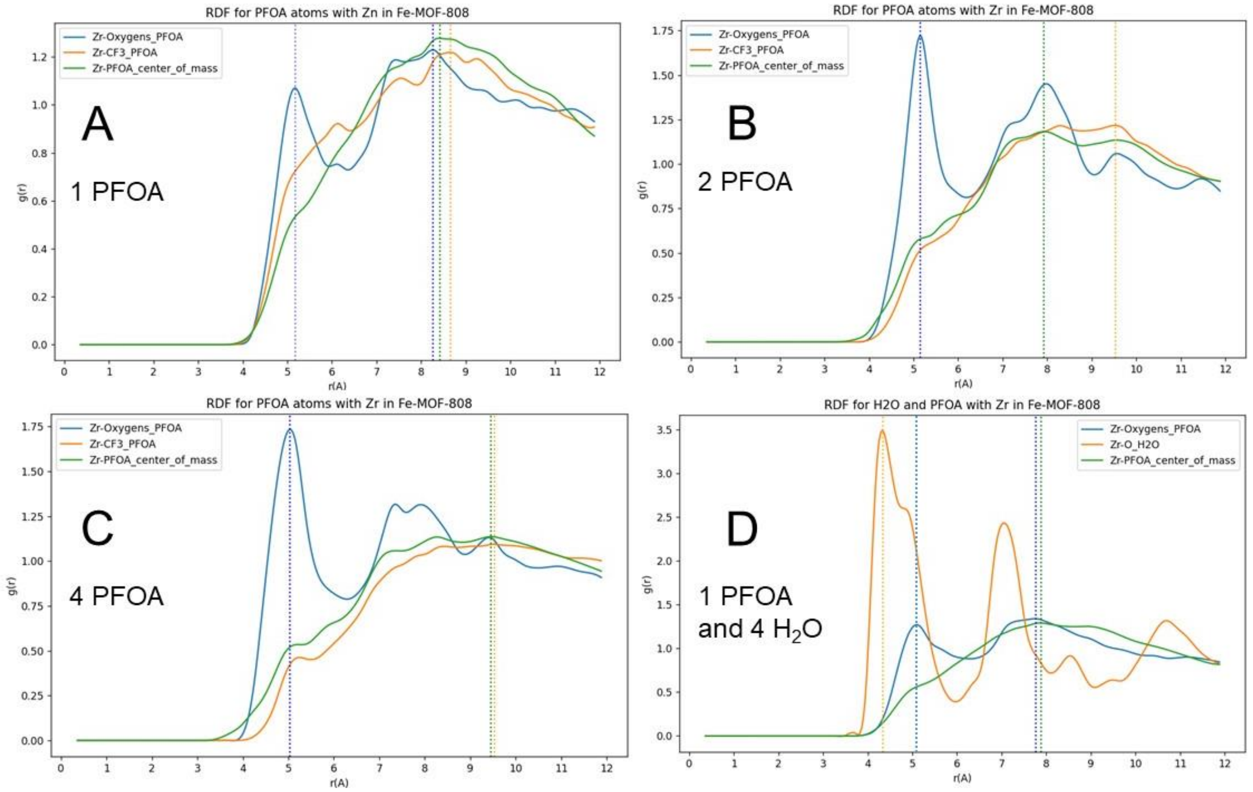

Figure S29. A), B) and C) Radial Distribution Functions (RDFs) of PFOA with respect to the zirconium atoms in Fe-MOF-808 with 1, 2 and 4 PFOAs per unit cell respectively, D) RDF of PFOA and H_2O with respect to the zirconium atoms in Fe-MOF-808 in the system with 1 PFOA and 4 water molecules per unit cell.

In the case of the system containing one PFOA molecule (part A of Figure S29), the peak at ~5 Å is not the most intense, as previously observed in part B of Figure S27. However, upon increasing the PFOA loading, a clear trend emerges: the interaction with Zr sites becomes more pronounced, and the peak at 5 Å reappears as the dominant feature. This behaviour is consistent with the DFT results, which showed that in Fe–MOF-808, configurations with a greater number of PFOA molecules preferentially coordinated to Zr sites were energetically more favourable than those bound to Fe sites.

Upon increasing PFOA concentrations in the port, the intensity of the peak at ~5 Å increases (parts B and C of Figure S29), suggesting that interaction of with the Zr sites is favoured for all PFOA molecules. In Figure part D of Figure S29, which represents the system containing one PFOA and four water molecules per pore, a similar trend is observed as in the pristine MOF-808 system (part

D of Figure S28). The characteristic Zr-$O_{(PFOA)}$ interaction peak remains at the same distance as in the corresponding "dry" system (one PFOA, no water; part A in Figure S29), confirming that the presence of water does not disrupt this key interaction. Additionally, water molecules also interact with the open Zr sites, displaying a shorter interaction distance of ~4.3 Å and a higher peak intensity, which can be attributed to their greater abundance. Concluding, the presence of water molecules does not significantly alter the PFOA binding inside the Fe-MOF-808.

Supplementary Note 12. PFAS capture experiments and analysis

General method for experiments in ppm pollutant concentrations: All capture tests were carried out by adding a predetermined amount of MOF adsorbent into a 20 mL glass capped vial containing an aqueous PFAS solution (either PFOA, PFHxA or PFBA) of already known concentration. The mixture was stirred at 25 ºC and the capture was studied at different PFAS initial concentrations. For PFOA, depending on the concentration of pollutant, the pH of the initial solution was found to be in the 3-5 range. For the experiments using competing ions, the water matrix also contained corresponding ionic species (detailed in Table S8) in a general ion:PFOA concentration ratio of 1:2.5. Based on previous studies in the literature initial pollutant concentrations were set at 100 mg/L, and the reaction time was set at 24 h.[27] All experiments were performed at least twice to ensure reproducibility. After the end of the designated time, the supernatant was then filtered through a 0.22 μm nylon filter (preliminary experiments confirmed no pollutant is captured during this step).

Post-capture analysis of solution: To evaluate the amount of fluorinated pollutant remaining in the supernatant, quantitative ^{19}F-NMR was then performed using a TFE/D_2O internal standard of predetermined concentration. Each NMR spectra was collected using a tube containing 700 μL of the solution and 50 μL of the TFE/D_2O standard. The concentration of PFAS was related to the one of TFE using the following equation:

$$[PFAS] = \frac{I_{PFAS}}{I_{TFE}} \cdot [TFE] \quad (1)$$

In this equation [PFAS] and [TFE] are molar concentrations, while I_{PFAS} and I_{TFE} are the integrals of each compound from the ^{19}F-NMR spectrum. The $–CF_3$ peak of the pollutant (-80.7 ppm for PFOA, -75 ppm for TFA) was always used for these calculations. The following parameters were used to ensure quantitative integration, as already reported in the literature: R0 = 0 Hz, NS = 16 scans, D1 = 20 s, TE = 300 K.[28] Capture performance was then analysed by calculating the difference in PFAS concentration before (blank experiment) and after the reaction. To evaluate capacity (mg_{PFAS}/g_{MOF}), the amount of contaminant adsorbed at equilibrium (q_e) was calculated from the following mass balance equation:

$$q_e = \frac{(C_0 - C_e)}{m} \cdot V \qquad (2)$$

where C_0 and C_e are the respective pollutant concentrations (mg/L) before and after the experiment, V is the volume (mL) of the PFAS solution and m is the mass (g) of the adsorbent used.

Adsorption isotherm studies for selected MOF materials were performed by fitting the calculated capacities at different PFAS concentrations (0-1200 ppm) using the Langmuir (equation 3) and Freundlich (equation 4) models:

$$q_e = \frac{(b \cdot C_e \cdot q_m)}{(1 + b \cdot C_e)} \qquad (3)$$

$$q_e = K_f \cdot C_e^{1/n} \qquad (4)$$

where C_e is the equilibrium PFAS concentration (mg/L), q_e is the equilibrium adsorption capacity (mg/g), q_m is the maximum adsorption capacity, b is bonding energy constant or Langmuir constant (L/mg), K_f [(mg/g) (L/mg)$^{1/n}$] is the Freundlich constant at equilibrium and 1/n (L/g) is the Freundlich exponential factor.

Post-capture analysis of the solid material: After the capture experiment was complete and the supernatant was filtered, the solid was decanted and washed with water and acetone (three times each), then dried at 60 ºC for 24 h. The material was then characterized with PXRD, NMR, SEM, FT-IR, TGA and PDF techniques (See relevant Sections).

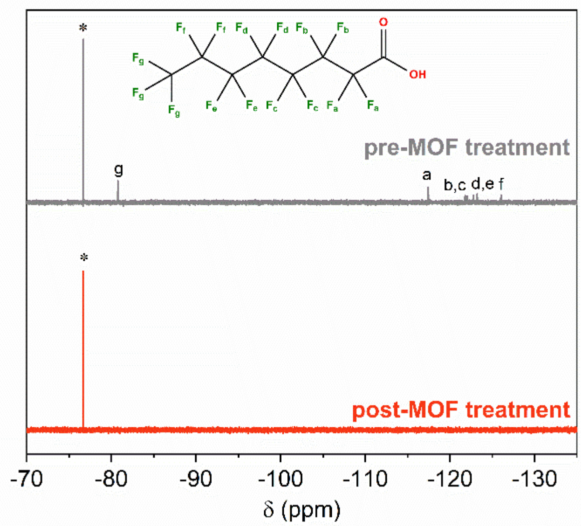

Figure S30. ^{19}F-NMR spectra of an aqueous PFOA solution (C_0 = 100 ppm) before and after treatment with Fe-MOF-808, showing complete pollutant removal. The internal standard (TFE) is marked with an asterisk.

General method for experiments in ppb PFAS concentrations: Initially, a mixture of the target PFAS analytes (listed in Table S6) was made, with an initial concentration of 0.1 mg/mL for each individual PFAS. Individual PFAS standards in either salt or liquid forms were dissolved in methanol (MeOH) and then combined into a single PFAS stock solution, which was then diluted with MilliQ water to reach the desired concentration.

The capture experiments were then carried out as follows: 20 mg of MOF adsorbent were first added into a 50 mL falcon tube, followed by 20 mL of MilliQ water pre-spiked with 0.1 mL of the PFAS mixture solution (final concentration for each individual PFAS: 0.25 μg/mL). The tube was shaken at 25 ºC for 24 hours. All experiments were performed at least twice to ensure reproducibility. After the end of the designated time, the supernatant was then separated via centrifugation (3000 rpm for 15 min). An aliquot (0.25 mL) of the supernatant was then transferred to a chromatographic glass vial containing 0.20 mL of methanol and 0.05 mL of the internal standard. ^{13}C mass-labelled internal standards for PFAS, as detailed in Table S6, were used throughout the analysis.

The analysis of PFAS was performed as described elsewhere[29] using an ultra-high performance liquid chromatography with tandem mass spectrometry (UPLC-MS/MS) instrument, a Sciex TQ-S 3500 triple quadrupole mass spectrometer furnished with a heated electrospray ionization source. Chromatographic separation was performed in a Phenomenex Kinetix® C18 pre-column (1.7 µm, 100 A). For analysis, a Phenomenex Gemini® 3 µm C18 HPLC analytical column (2 x 2.1 mm, 3 µm, 110 A) with a Phenomenex KJ0-4282 analytical guard column was used. Chromatographic separation was achieved with Milli-Q water 10 mM ammonium acetate (A) and pure MeOH (B), as mobile phases at a constant flow rate of 600 µL min^{-1} and constant column temperature of 40ºC. The autosampler temperature was held constant at 15°C. The mobile phase gradient program was as follows: starting: 0.0 min 5% B, 0.0-0.1 min 55% B, 0.1-4.4 min 99% B, 4.5-8-0 min 99% B,8.0 to 8.5 min 5% B, 8.5 to 12 min 5% B. The injection volume was 10 µL. Analytes were ionized under negative ionization mode (ESI). Fits of $R^2 > 0.99$ were obtained from the generated calibration curves.

Table S7. Determination parameters of PFAS target analytes in the UPLC-MS-MS instrument. Mass labelled internal standards were available for most individual analytes.

PFAS analyte	Formula	Precursor mass (Q1, m/z)	Product mass (Q3, m/z)	Retention time (min)	Declustering potential (V)	Collision energy (V)	Collision cell exit Potential (V)
PFBA	$C_4HF_9O_2$	213	168.9	1.06	-20	-12	-5
PFPeA	$C_5HF_9O_2$	262.9	218.9	1.27	-5	-12	-11
PFHxA	$C_6HF_{11}O_2$	313.0	268.9	1.56	-35	-10	.14
PFHpA	$C_7HF_{13}O_2$	362.9	318.9	1.93	-40	-14	-11
			169.0			-24	-5
PFOA	$C_8HF_{15}O_2$	413.0	369.1	2.32	-45	-16	-15
			169.1			-24	-5
6:2 FTSA	$C_8H_5F_{13}O_3S$	429.0	81.0	2.28	-90	-70	-7
PFOS	$C_8F_{17}O_3S$	499.2	80.0	2.71	-130	-110	-7
			98.9		-115	-94	-9
PFNA	$C_9HF_{17}O_2$	463.0	219.0	2.71	-50	-24	-9
			168.9			-26	-9
PFDA	$C_{10}HF_{19}O_2$	513.0	268.9	3.09	-55	-26	-11
			218.9		-50	-26	-9
8:2 FTSA	$C_{10}H_5F_{15}O_3S$	527.2	506.9	3.08	-125	-38	-15
			81		-110	-88	-7
PFUnDA	$C_{11}HF_{21}O_2$	563.0	268.9	3.43	-60	-18	-9
					-55	-26	-11
PFDoDA	$C_{12}HF_{23}O_2$	613.0	569.0	3.74	-65	-18	-11
			318.9			-28	-13

Internal standards used							
**Acronym**	**Molecular formula**	**Precursor mass (Q1, m/z)**	**Product mass (Q3, m/z)**	**Retention time (min)**	**Declustering potential (V)**	**Collision energy (V)**	**Collision cell exit Potential (V)**
13C4-PFBA	$^{13}C_4F_7O_2$	217.0	172.0	1.06	-25	-14	-9
13C5-PFPeA	$^{13}C_5F_9O_2$	268.0	223.0	1.27	30	-12	-9
13C5-PFHxA	$^{13}C_5{}^{12}C_1F_{11}O_2$	318.0	273.0	1.56	-35	-14	-13
13C4-PFHpA	$^{13}C_4{}^{12}C_3F_{12}O_2$	367.0	172.0	1.93	-40	-24	-7
13C8-PFOA	$^{13}C_8F_{15}O_2$	421.0	376.0	2.32	-45	-16	-17
13C2-6:2 FTSA	$^{13}C_2{}^{12}C_6H_4F_{13}SO_3$	429	81.0	2.28	-90	-70	-7
13C8-PFOS	$^{13}C_8F_{17}SO_3$	507.0	80.0	2.71	-135	-108	-7
13C9-PFNA	$^{13}C_9F^{17}O_2$	472.0	172.0	2.71	-50	-26	-9
13C6-PFDA	$^{13}C_6{}^{12}C_4F_{19}O_2$	519.0	474.0	3.09	-75	-14	-19
13C2-8:2 FTSA	$^{13}C_2{}^{12}C_8H_4F_{17}SO_3$	529.0	81.0	3.08	-120	-86	-7
13C7-PFUnDA	$^{13}C_7{}^{12}C_4F_{21}O_2$	570.0	525.0	3.43	-90	-18	-9
13C3-PFDoDA	$^{13}C_2{}^{12}C_{10}F_{23}O_2$	615.0	570.0	3.74	-65	-18	-11

Regeneration and Recyclability Tests: To regenerate Fe-MOF-808 after a PFOA capture experiment, the material was immersed in a 0.15 M Na_2SO_4 solution for 16 h at 50 °C. Non-coordinating PFOA molecules can be also removed by simply washing the material with water and acetone (three times each). Recycling capture studies for non-coordinating PFOA were performed for a total of 8 cycles; during each cycle, an aqueous PFOA solution of 100 ppm and the same dosing described in the general method were employed. Considering the adsorption kinetics results, the adsorbent was immersed to the aqueous PFOA solution for 2 hours per cycle. The isolated solid material was washed (three times with water, three times with acetone) and dried under vacuum. Occasional characterizations of the MOF with PXRD and ICP were also performed at selected cycles (see Figure S3 and Table S1 respectively), to verify possible issues of stability or iron leaching.

Table S8. PFOA adsorption capacity values during screening tests for the materials of this study. Experimental conditions: 3 mg of MOF were immersed to 20 mL of ~100 mg/L PFOA solutions. All experiments were performed at least two times and average values are reported.

MOF material	Removal Efficiency (%)	Adsorption Capacity (mg_{PFOA} / g_{MOF})
MOF-808[a]	30	282.5
Fe-MOF-808	100	744

[a] Reported value from reference 1.

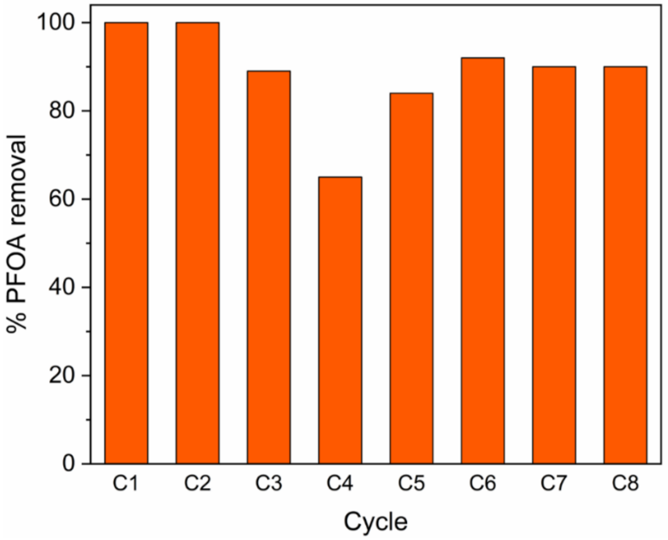

Figure S31. PFOA removal efficiency of Fe-MOF-808 after multiple cycles. Regeneration and recyclability conditions are detailed at the beginning of this Supplementary Note. Stability of the material in various cycles was evaluated by PXRD (Figure S3) and ICP (Table S1). Considering that the MOF remains stable and no leaching is observed, the drop in performance during C4 is attributed to insufficient washing during reactivation (likely resulting in residual PFOA remaining within the MOF).

Table S9. Actual concentrations of ionic species used for the PFOA capture test with competing ions.

**Compound used**	**Concentrations (ppm)**
$Mg(NO_3)_2$	40 / 400
$CaSO_4$	38 / 385
NaCl	48 / 405
$FeCl_3$	42 / 390
$NaNO_3$	44 / 420
$Al(NO_3)_3$	42 / 400
Na_2SO_4	38 / 460

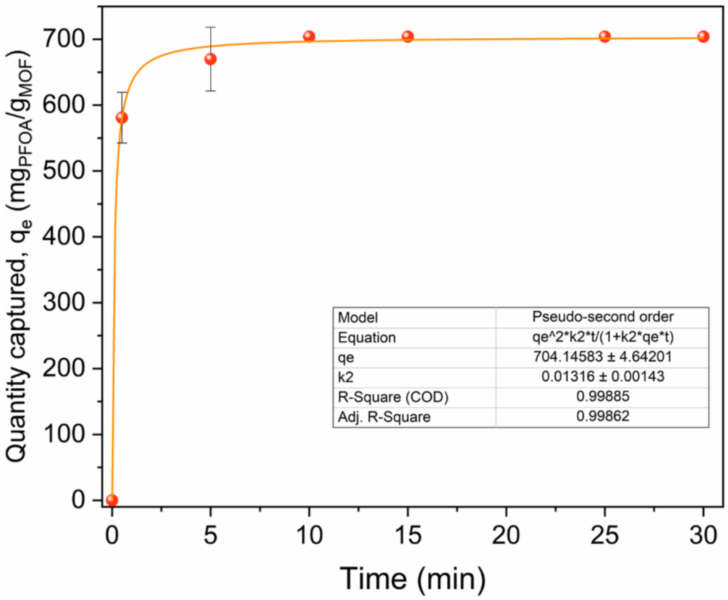

Figure S32. Pseudo-second order fit for the kinetics of PFOA adsorption using Fe-MOF-808.

Table S10. Capacity values over time from the PFOA adsorption kinetics test using Fe-MOF-808. All experiments were performed at least two times and average values are reported.

**Time (min)**	**Adsorption Capacity (mg_{PFOA} / g_{MOF})**
0	0
0.5	581
5	670
10	704
15	704
25	704
30	704

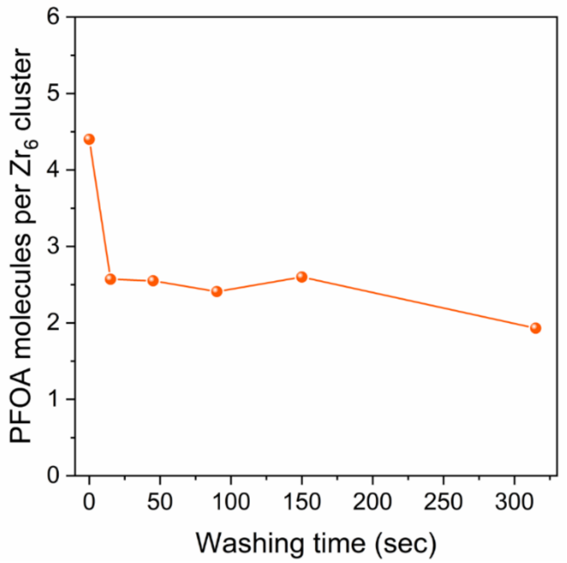

Figure S33. PFOA desorption kinetics after a capture experiment using 100 ppm of pollutant. Work-up conditions and detailed values are provided in Table S10.

Table S11. Work-up conditions and corresponding chemical formula analyses (determined by ^{1}H- and ^{19}F-NMR) for the desorption kinetics results of Figure S29.

Time (sec)	Work-up	Acetate molecules per Zr_6 cluster	PFOA molecules per Zr_6 cluster
0	Isolate solid, no washing	0.9	4.4
15	Isolate solid, 1 H_2O wash	1.0	2.57
45	Isolate solid, 1 H_2O wash, 30 sec centrifugation	0.6	2.55
90	Isolate solid, 2 H_2O washes, 30 sec centrifugation per wash	0.5	2.41
150	Isolate solid, 2 H_2O washes, 60 sec centrifugation per wash	0.6	2.60
315	Isolate solid, 3 H_2O washes, 90 sec centrifugation per wash	0.5	1.93

Table S12. PFOA adsorption kinetics parameters of the highest-performing MOF materials of the literature, including the one of Fe-MOF-808 presented in this work.

MOF material	PFOA concentration (ppm)	PFOA adsorption kinetics (t_e = time until equilibrium)	Ref.
Fe-MOF-808	100	t_e = 10 min<br>$k_2 = 1.32\times10^{-2}$ g mg^{-1} min^{-1}	This work

NU-1000	100	t_e = 1 min	30
UiO-66-$N(CH_3)_3^+$	500	t_e = N/A<br>$k_2 = 0.93\times10^{-4}$ g mg^{-1} h^{-1}	31
PCN-999	1000	t_e = 12 h<br>$k_2 = 0.23\times10^{-4}$ g mg^{-1} min^{-1}	32
Cr-MIL-101-QDMEN	1000	t_e < 60 min<br>$k_2 = 0.21\times10^{-3}$ g mg^{-1} min^{-1}	33
Cr-MIL-101	1000	t_e < 60 min<br>$k_2 = 0.14\times10^{-3}$ g mg^{-1} min^{-1}	33
Cr-MIL-101-DMEN	1000	t_e < 60 min<br>$k_2 = 0.11\times10^{-3}$ g mg^{-1} min^{-1}	33
Cr-MIL-101-NMe_3	1000	t_e < 60 min<br>$k_2 = 0.16\times10^{-3}$ g mg^{-1} min^{-1}	33
Cr-MIL-101-NH_2	1000	t_e < 60 min<br>$k_2 = 0.15\times10^{-3}$ g mg^{-1} min^{-1}	33
UiO-67	500	t_e < 60 min<br>$k_2 = 0.61\times10^{-3}$ g mg^{-1} min^{-1}	34
UiO-66	500	t_e = 10 min<br>$k_2 = 0.273\times10^{-2}$ g mg^{-1} min^{-1}	35
UiO-66-F4	1000	t_e = 10 min<br>$k_2 = 0.257\times10^{-2}$ g mg^{-1} min^{-1}	35
ZIF-L	207	t_e > 60 min	36
ZIF-8	207	t_e > 60 min	36
ZIF-7	207	t_e > 60 min	36

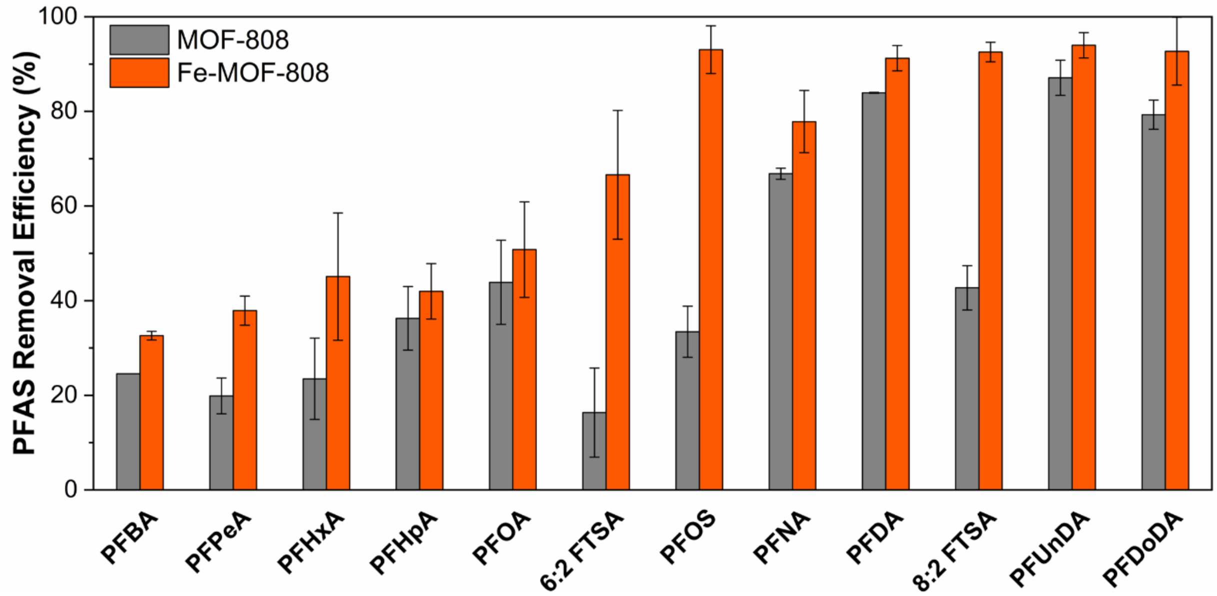

Figure S34. PFAS removal efficiencies of MOF-808 and Fe-MOF-808 at ppb levels ($[PFAS]_{indiv.}$ = 250 ppb). All experiments were performed at least two times and average values are reported.

Table S13. PFBA and PFHxA adsorption performance in ppm levels using Fe-MOF-808. Experimental conditions: 3 mg of MOF were immersed to 20 mL of 100 mg/L PFAS solutions. All experiments were performed at least two times and average values are reported.

MOF material	Removal Efficiency / Adsorption Capacity (mg_{PFAS} / g_{MOF})	
	PFBA	PFHxA
Fe-MOF-808	20 % / 175	50 % / 393

Table S14. Capacity values for Fe-MOF-808 after PFOA adsorption in various pollutant concentrations. Experimental conditions: 3 mg of MOF were immersed to 20 mL of ~100-1200 mg/L PFOA solutions. All experiments were performed at least two times and average values are reported.

MOF material	PFOA concentration (ppm)	Adsorption Capacity (mg_{PFOA} / g_{MOF})
Fe-MOF-808	109	744
	243	1147
	496	1410
	1011	2081
	1131	2371

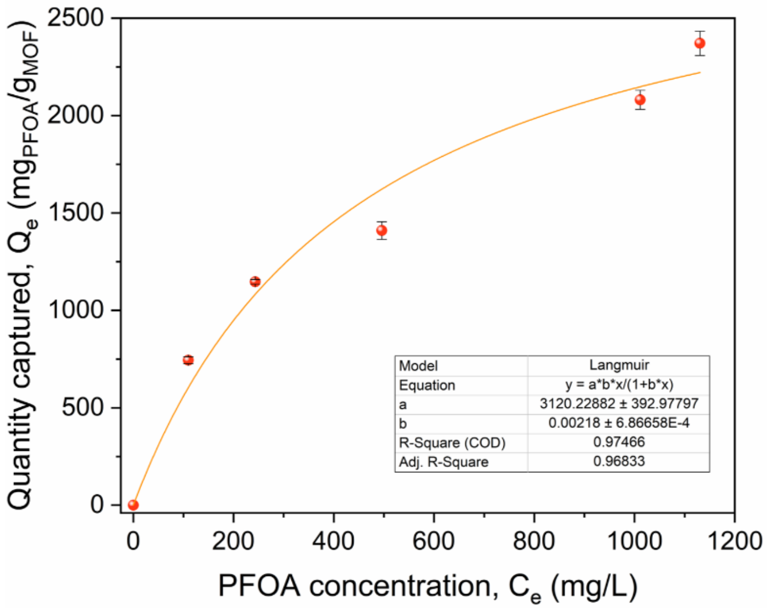

Figure S35. Langmuir fit for PFOA adsorption using Fe-MOF-808.

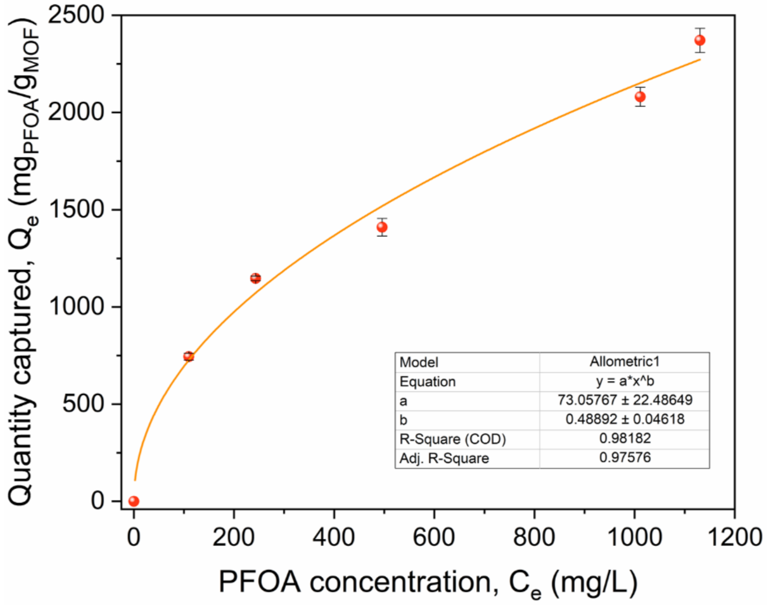

Figure S36. Freundlich fit for PFOA adsorption using Fe-MOF-808.

Table S15. Comparable PFOA adsorption capacity parameters of the highest-performing reported MOF materials, including the one presented in this work.

**MOF material**	**Experimental PFOA uptake (mg/g) at 1000 ppm concentration (ppm)**	**Maximum PFOA adsorption capacity[a] (mg/g) / Concentration range (ppm)**	**Ref.**
Fe-MOF-808	2081	3120 / 100-1200	This work
TFA-MOF-808	1786	2496 / 100-800	[1]
MOF-808	1091	1581 / 100-800	[1]
UiO-67-F2	928	3060 / 150-3232	[37]
PCN-999	764	1089 / 100-5000	[32]
Cr-MIL-101-QDMEN	754	0-250	[33]
UiO-67	872	1589 / 500-3232	[37]
UiO-66-$N(CH_3)_3^+$	665	1178 / 0-1000	[31]
Cr-MIL-101-DMEN	534	0-250	[33]
Cr-MIL-101-NMe_3	493	0-250	[33]
UiO-66-L_3	-	403 / 0-100	[38]
DUT-5-2-Al	-	98 / 10-140	[39]
UiO-66-F4	-	467 / 0-350	[35]
Cr-MIL-101	460	0-250	[33]
Fe-BTC	418	548 / 50-1000	[40]
UiO-66	-	388 / 0-600	[35]
MIL-101-Fe	370	490 / 50-1000	[40]
MIL-100-Fe	349	427 / 50-1000	[40]
MIL-96-RHPAM2	270	340 / 0-1000	[41]
Cr-MIL-101-NH_2	290	0-250	[33]
Ce-BTC	210	-	[40]
Mn-BTC	130	-	[40]

[a] As calculated by the Langmuir model.

Table S16. PFOA adsorption performance of Fe-MOF-808 in context to other high-performing porous materials of the literature.

Material	PFOA adsorption capacity (mg/g)	Ref.
Fe-MOF-808	3120	This work
MELEM-COF	2500	[42]
TG-PD COF	>2500	[43]
Granular activated carbon (GAC)	426.5	[44]
Bamboo-derived GAC	161.5	[45]
Powder activated carbon (PAC)	277.4	[44]
Anion-exchange resin AI400	1209	[44]
PAF-1-NDMB	2000	[46]
DFB β-cyclodextrin polymer	33	[47]
Crosslinked β-cyclodextrin	457	[48]

Table S17. PFBA and PFHxA adsorption performance of all reported porous materials, including the ones presented in this work.

MOF material	Pollutant	PFAS concentration (ppm)	PFAS adsorption capacity (mg/g)	Reference
TFA-MOF-808	PFBA	100	311	[1]
NU-1000	PFBA	100	274	[30]
Hollow CysCOF	PFBA	350	246	[49]
Fe-MOF-808	PFBA	100	175	This work
pDADMAC-modified GAC	PFBA	0-10000	165	[50]
CTF	PFBA	7-204	92	[51]
Microporous AC	PFBA	7-204	51	[51]
Monofunct. PEGDA	PFBA	106	43	[52]
Bifunct. PEGDA	PFBA	106	34	[52]
β-zeolite CP811C	PFBA	5-500	28	[53]
all-silica zeolite β	PFBA	500	25	[54]
TFA-MOF-808	PFHxA	100	436	[1]
Fe-MOF-808	PFHxA	100	393	This work
CTF	PFHxA	7.2-217	377	[51]

Microporous AC	PFHxA	7.2-217	236	[51]
all-silica zeolite β	PFHxA	500	200	[54]

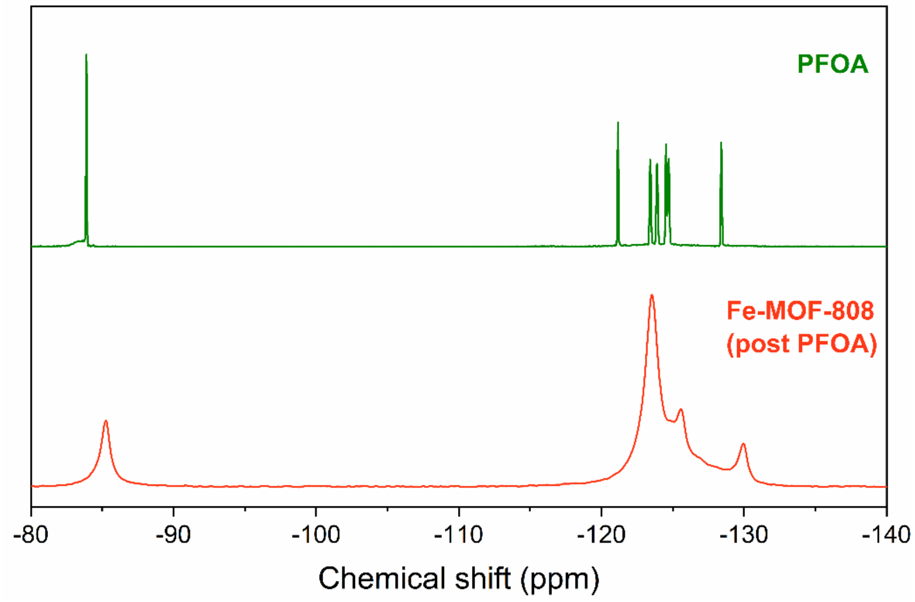

Figure S37. Solid-state ^{19}F-NMR spectra of PFOA and Fe-MOF-808 after PFOA sorption.

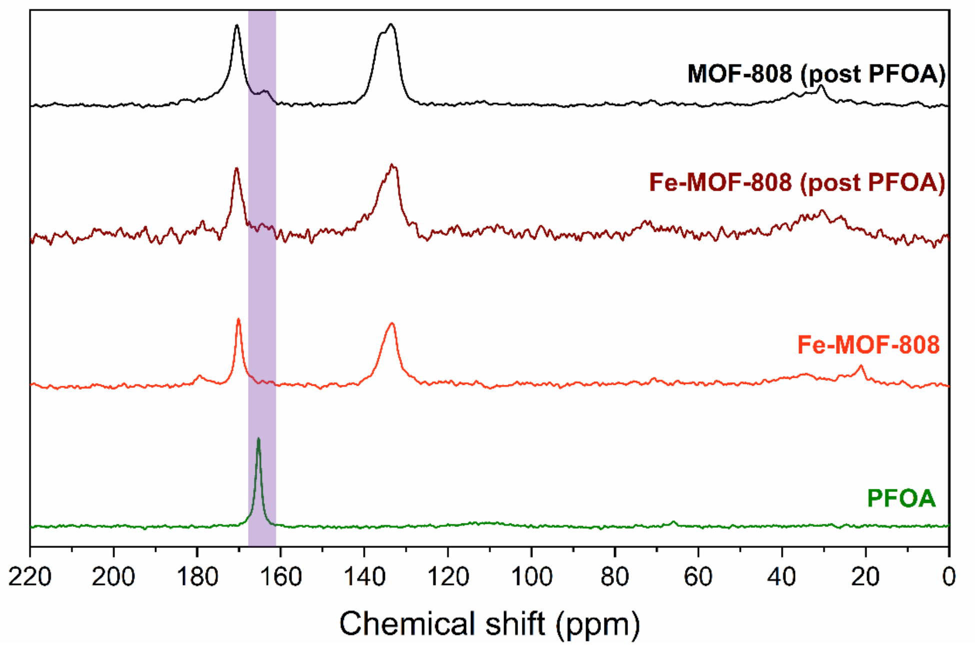

Figure S38. Solid-state ^{13}C-NMR spectra of PFOA, as-made Fe-MOF-808, Fe-MOF-808 and MOF-808 after PFOA sorption.

Table S18. Chemical analysis results for Fe-MOF-808 materials after PFOA adsorption in various pollutant concentrations, using ^{1}H and ^{19}F-NMR. Experimental conditions: 3 mg of MOF were immersed to 20 mL of ~100-1200 mg/L PFOA solutions. All experiments were performed two times and average values are reported.

[PFOA] (ppm)	Formula	Molecules PFOA / Zr_6 cluster	Molecules PFOA found in pore
0	$Zr_6Fe_{3.65}O_4(OH)_4(BTC)_2(HCO_2)_{0.59}(OAc)_{3.38}(H_2O)_{12.98}(OH)_{12.98}$	-	-
100	$Zr_6Fe_{3.65}O_4(OH)_4(BTC)_2(PFOA)_{2.76}(HCO_2)_{0.8}(OAc)_{0.35}(H_2O)_{13.04}(OH)_{13.04}$	2.76	1.8
200	$Zr_6Fe_{3.65}O_4(OH)_4(BTC)_2(PFOA)_{3.11}(HCO_2)_{0.9}(OAc)_{0.4}(H_2O)_{12.54}(OH)_{12.54}$	3.11	4.1
400	$Zr_6Fe_{3.65}O_4(OH)_4(BTC)_2(PFOA)_{3.60}(HCO_2)_{0.8}(OAc)_{0.2}(H_2O)_{12.35}(OH)_{12.35}$	3.60	5.5
1000	$Zr_6Fe_{3.65}O_4(OH)_4(BTC)_2(PFOA)_{3.92}(HCO_2)_{0.9}(OAc)_{0.14}(H_2O)_{11.99}(OH)_{11.99}$	3.92	10.8
1150	$Zr_6Fe_{3.70}O_4(OH)_4(BTC)_2(PFOA)_{5.34}(HCO_2)_{0.5}(OAc)_1(H_2O)_{10.26}(OH)_{10.26}$	5.34	15.2

Supplementary References

1. Loukopoulos, E.; Marugán-Benito, S.; Raptis, D.; Tylianakis, E.; Froudakis, G. E.; Mavrandonakis, A.; Platero-Prats, A. E., Chemically Tailored Metal-Organic Frameworks for Enhanced Capture of Short- and Long-Chain Per- and Polyfluoroalkyl Substances from Water. *Adv. Funct. Mater.* **2024,** *34* (51), 2409932. DOI: 10.1002/adfm.202409932.
2. Macrae, C. F.; Edgington, P. R.; McCabe, P.; Pidcock, E.; Shields, G. P.; Taylor, R.; Towler, M.; van De Streek, J., Mercury: visualization and analysis of crystal structures. *J. Appl. Crystallogr.* **2006,** *39,* 453-457. DOI: 10.1107/S002188980600731x.
3. Castillo-Blas, C.; Romero-Muñiz, I.; Mavrandonakis, A.; Simonelli, L.; Platero-Prats, A. E., Unravelling the local structure of catalytic Fe-oxo clusters stabilized on the MOF-808 metal organic-framework. *Chem Commun* **2020,** *56* (100), 15615-15618. DOI: 10.1039/d0cc06134d.
4. Jumpathong, W.; Pila, T.; Lekjing, Y.; Chirawatkul, P.; Boekfa, B.; Horike, S.; Kongpatpanich, K., Exploitation of missing linker in Zr-based metal-organic framework as the catalyst support for selective oxidation of benzyl alcohol. *APL Materials* **2019,** *7* (11). DOI: 10.1063/1.5126077.
5. Otake, K. I.; Ahn, S.; Knapp, J.; Hupp, J. T.; Notestein, J. M.; Farha, O. K., Vapor-Phase Cyclohexene Epoxidation by Single-Ion Fe(III) Sites in Metal-Organic Frameworks. *Inorganic Chemistry* **2021,** *60* (4), 2457-2463. DOI: 10.1021/acs.inorgchem.0c03364.
6. Yang, J.; Gao, M.; Wang, S.; Zhang, M.; Chen, L.; Su, J.; Huang, Y.; Zhang, Y.; Wang, X.; Shen, B., Experimental and Simulation Studies of the Adsorption of Methylbenzene by Fe(III)-Doped NU-1000 (Zr). *ACS Applied Materials and Interfaces* **2022,** *14* (35), 40052-40061. DOI: 10.1021/acsami.2c11700.

7. Juhás, P.; Davis, T.; Farrow, C. L.; Billinge, S. J. L., PDFgetX3: A rapid and highly automatable program for processing powder diffraction data into total scattering pair distribution functions. *J Appl Crystallogr* **2013,** *46* (2), 560-566. DOI: 10.1107/S0021889813005190.
8. Ravel, B.; Newville, M., ATHENA, ARTEMIS, HEPHAESTUS: Data analysis for X-ray absorption spectroscopy using IFEFFIT. *Journal of Synchrotron Radiation* **2005,** *12*, 537-541. DOI: 10.1107/S0909049505012719.
9. Rehr, J. J.; Kas, J. J.; Vila, F. D.; Prange, M. P.; Jorissen, K., Parameter-free calculations of X-ray spectra with FEFF9. *Physical Chemistry Chemical Physics* **2010,** *12* (21), 5503-5513. DOI: 10.1039/b926434e.
10. del Castillo-Velilla, I.; Sousaraei, A.; Romero-Muñiz, I.; Castillo-Blas, C.; S. J. Méndez, A.; Oropeza, F. E.; de la Peña O'Shea, V. A.; Cabanillas-González, J.; Mavrandonakis, A.; Platero-Prats, A. E., Synergistic binding sites in a metal-organic framework for the optical sensing of nitrogen dioxide. *Nature Communications* **2023,** *14* (1). DOI: 10.1038/s41467-023-38170-9.
11. Yang, B.; Wheeler, J. I.; Sorensen, B.; Steagall, R.; Nielson, T.; Yao, J.; Mendez-Arroyo, J.; Ess, D. H., Computational determination of coordination structure impact on adsorption and acidity of pristine and sulfated MOF-808. *Materials Advances* **2021,** *2* (13), 4246-4254. DOI: 10.1039/D1MA00330E.
12. Frisch, M. J.; Trucks, G. W.; Schlegel, H. B.; Scuseria, G. E.; Robb, M. A.; Cheeseman, J. R.; Scalmani, G.; Barone, V.; Petersson, G. A.; Nakatsuji, H.; Li, X.; Caricato, M.; Marenich, A. V.; Bloino, J.; Janesko, B. G.; Gomperts, R.; Mennucci, B.; Hratchian, H. P.; Ortiz, J. V.; Izmaylov, A. F.; Sonnenberg, J. L.; Williams; Ding, F.; Lipparini, F.; Egidi, F.; Goings, J.; Peng, B.; Petrone, A.; Henderson, T.; Ranasinghe, D.; Zakrzewski, V. G.; Gao, J.; Rega, N.; Zheng, G.; Liang, W.; Hada, M.; Ehara, M.; Toyota, K.; Fukuda, R.; Hasegawa, J.; Ishida, M.; Nakajima, T.; Honda, Y.; Kitao, O.; Nakai, H.; Vreven, T.; Throssell, K.; Montgomery Jr., J. A.; Peralta, J. E.; Ogliaro, F.; Bearpark, M. J.; Heyd, J. J.; Brothers, E. N.; Kudin, K. N.; Staroverov, V. N.; Keith, T. A.; Kobayashi, R.; Normand, J.; Raghavachari, K.; Rendell, A. P.; Burant, J. C.; Iyengar, S. S.; Tomasi, J.; Cossi, M.; Millam, J. M.; Klene, M.; Adamo, C.; Cammi, R.; Ochterski, J. W.; Martin, R. L.; Morokuma, K.; Farkas, O.; Foresman, J. B.; Fox, D. J. *Gaussian 16 Rev. C.01*, Wallingford, CT, 2016.
13. Zhao, Y.; Truhlar, D. G., A new local density functional for main-group thermochemistry, transition metal bonding, thermochemical kinetics, and noncovalent interactions. *The Journal of Chemical Physics* **2006,** *125* (19). DOI: 10.1063/1.2370993.
14. Andrae, D.; Häußermann, U.; Dolg, M.; Stoll, H.; Preuß, H., Energy-adjustedab initio pseudopotentials for the second and third row transition elements. *Theoretica chimica acta* **1990,** *77* (2), 123-141. DOI: 10.1007/BF01114537.
15. Hehre, W. J.; Ditchfield, R.; Pople, J. A., Self—Consistent Molecular Orbital Methods. XII. Further Extensions of Gaussian—Type Basis Sets for Use in Molecular Orbital Studies of Organic Molecules. *The Journal of Chemical Physics* **1972,** *56* (5), 2257-2261. DOI: 10.1063/1.1677527.
16. Marenich, A. V.; Cramer, C. J.; Truhlar, D. G., Universal Solvation Model Based on Solute Electron Density and on a Continuum Model of the Solvent Defined by the Bulk Dielectric Constant and Atomic Surface Tensions. *The Journal of Physical Chemistry B* **2009,** *113* (18), 6378-6396. DOI: 10.1021/jp810292n.
17. Grimme, S., Supramolecular Binding Thermodynamics by Dispersion-Corrected Density Functional Theory. *Chemistry – A European Journal* **2012,** *18* (32), 9955-9964. DOI: https://doi.org/10.1002/chem.201200497.

18. Lu, T.; Chen, Q., Shermo: A general code for calculating molecular thermochemistry properties. *Computational and Theoretical Chemistry* **2021,** *1200*, 113249. DOI: https://doi.org/10.1016/j.comptc.2021.113249.
19. Lu, T.; Chen, Q., Interaction Region Indicator: A Simple Real Space Function Clearly Revealing Both Chemical Bonds and Weak Interactions. *Chemistry–Methods* **2021,** *1* (5), 231-239. DOI: https://doi.org/10.1002/cmtd.202100007.
20. Lu, T., A comprehensive electron wavefunction analysis toolbox for chemists, Multiwfn. *The Journal of Chemical Physics* **2024,** *161* (8), 082503. DOI: 10.1063/5.0216272.
21. Rappe, A. K.; Casewit, C. J.; Colwell, K. S.; Goddard, W. A., III; Skiff, W. M., UFF, a full periodic table force field for molecular mechanics and molecular dynamics simulations. *Journal of the American Chemical Society* **1992,** *114* (25), 10024-10035. DOI: 10.1021/ja00051a040.
22. Zhao, G.; Chung, Y. G., PACMAN: A Robust Partial Atomic Charge Predicter for Nanoporous Materials Based on Crystal Graph Convolution Networks. *Journal of Chemical Theory and Computation* **2024,** *20* (12), 5368-5380. DOI: 10.1021/acs.jctc.4c00434.
23. Erkal, T. S.; Shamsuddin, N.; Kirmizialtin, S.; Yazaydin, A. O., Computational Investigation of Structure–Function Relationship in Fluorine-Functionalized MOFs for PFOA Capture from Water. *The Journal of Physical Chemistry C* **2023,** *127* (6), 3204-3216. DOI: 10.1021/acs.jpcc.2c07737.
24. Dubbeldam, D.; Calero, S.; Ellis, D. E.; Snurr, R. Q., RASPA: molecular simulation software for adsorption and diffusion in flexible nanoporous materials. *Molecular Simulation* **2016,** *42* (2), 81-101. DOI: 10.1080/08927022.2015.1010082.
25. Abascal, J. L. F.; Vega, C., A general purpose model for the condensed phases of water: TIP4P/2005. *The Journal of Chemical Physics* **2005,** *123* (23), 234505. DOI: 10.1063/1.2121687.
26. Thompson, A. P.; Aktulga, H. M.; Berger, R.; Bolintineanu, D. S.; Brown, W. M.; Crozier, P. S.; in 't Veld, P. J.; Kohlmeyer, A.; Moore, S. G.; Nguyen, T. D.; Shan, R.; Stevens, M. J.; Tranchida, J.; Trott, C.; Plimpton, S. J., LAMMPS - a flexible simulation tool for particle-based materials modeling at the atomic, meso, and continuum scales. *Computer Physics Communications* **2022,** *271*, 108171. DOI: https://doi.org/10.1016/j.cpc.2021.108171.
27. Ahrens, L.; Bundschuh, M., Fate and effects of poly- and perfluoroalkyl substances in the aquatic environment: A review. *Environ. Toxicol. Chem* **2014,** *33* (9), 1921-1929. DOI: 10.1002/etc.2663.
28. Vanoursouw, T. M.; Rottiger, T.; Wadzinski, K. A.; Vanderwaal, B. E.; Snyder, M. J.; Bittner, R. T.; Farha, O. K.; Riha, S. C.; Mondloch, J. E., Adsorption of a PFAS Utilizing MOF-808: Development of an Undergraduate Laboratory Experiment in a Capstone Course. *Journal of Chemical Education* **2023,** *100* (2), 861-868. DOI: 10.1021/acs.jchemed.2c00910.
29. Nassazzi, W.; Lai, F. Y.; Ahrens, L., A novel method for extraction, clean-up and analysis of per- and polyfluoroalkyl substances (PFAS) in different plant matrices using LC-MS/MS. *J. Chromatogr. B* **2022,** *1212*, 123514. DOI: 10.1016/j.jchromb.2022.123514.
30. Li, R.; Alomari, S.; Stanton, R.; Wasson, M. C.; Islamoglu, T.; Farha, O. K.; Holsen, T. M.; Thagard, S. M.; Trivedi, D. J.; Wriedt, M., Efficient Removal of Per- And Polyfluoroalkyl Substances from Water with Zirconium-Based Metal-Organic Frameworks. *Chem Mater* **2021,** *33* (9), 3276-3285. DOI: 10.1021/acs.chemmater.1c00324.
31. Dalapati, R.; Shi, J.; Hunter, M.; Zang, L., Dual-functional metal-organic framework for efficient removal and fluorescent detection of perfluorooctanoic acid (PFOA) from water. *Journal of Materials Chemistry C* **2025,** *13* (32), 16753-16762. DOI: 10.1039/d5tc01765c.

32. Liang, R. R.; Xu, S.; Han, Z.; Yang, Y.; Wang, K. Y.; Huang, Z.; Rushlow, J.; Cai, P.; Samorì, P.; Zhou, H. C., Exceptionally High Perfluorooctanoic Acid Uptake in Water by a Zirconium-Based Metal-Organic Framework through Synergistic Chemical and Physical Adsorption. *J. Am. Chem. Soc.* **2024,** *146* (14), 9811-9818. DOI: 10.1021/jacs.3c14487.
33. Liu, K.; Zhang, S.; Hu, X.; Zhang, K.; Roy, A.; Yu, G., Understanding the Adsorption of PFOA on MIL-101(Cr)-Based Anionic-Exchange Metal-Organic Frameworks: Comparing DFT Calculations with Aqueous Sorption Experiments. *Environ Sci Technol* **2015,** *49* (14), 8657-65. DOI: 10.1021/acs.est.5b00802.
34. Sini, K.; Bourgeois, D.; Idouhar, M.; Carboni, M.; Meyer, D., Metal-organic frameworks cavity size effect on the extraction of organic pollutants. *Materials Letters* **2019,** *250*, 92-95. DOI: 10.1016/j.matlet.2019.04.113.
35. Sini, K.; Bourgeois, D.; Idouhar, M.; Carboni, M.; Meyer, D., Metal-organic framework sorbents for the removal of perfluorinated compounds in an aqueous environment. *New Journal of Chemistry* **2018,** *42* (22), 17889-17894. DOI: 10.1039/c8nj03312a.
36. Chen, M. J.; Yang, A. C.; Wang, N. H.; Chiu, H. C.; Li, Y. L.; Kang, D. Y.; Lo, S. L., Influence of crystal topology and interior surface functionality of metal-organic frameworks on PFOA sorption performance. *Microporous and Mesoporous Materials* **2016,** *236*, 202-210. DOI: 10.1016/j.micromeso.2016.08.046.
37. Hedbom, D.; Gaiser, P.; Günther, T.; Cheung, O.; Strømme, M.; Åhlén, M.; Sjödin, M., A fluorinated zirconium-based metal-organic framework as a platform for the capture and removal of perfluorinated pollutants from air and water. *J. Mater. Chem. A* **2024,** *13* (3), 1731-1737. DOI: 10.1039/d4ta06167e.
38. Luo, J.; Luo, F.; Li, H.; Mao, C.; Pan, Y.; Fang, Z.; Yu, D.; Liu, H.; Fu, K., Structure-Oriented Metal-Organic Framework Activation via Proximal Oligoalkyl Quaternary Ammonium Grafting Enhances Long-Chain PFAS Sorption. *Angew. Chem. Int. Ed.* **2025**, e202514746. DOI: 10.1002/anie.202514746.
39. Hu, Y.; Guo, M.; Zhang, S.; Jiang, W.; Xiu, T.; Yang, S.; Kang, M.; Dongye, Z.; Li, Z.; Wang, L., Microwave synthesis of metal-organic frameworks absorbents (DUT-5-2) for the removal of PFOS and PFOA from aqueous solutions. *Microporous and Mesoporous Materials* **2022,** *333*. DOI: 10.1016/j.micromeso.2022.111740.
40. Yang, Y.; Zheng, Z.; Ji, W.; Xu, J.; Zhang, X., Insights to perfluorooctanoic acid adsorption micro-mechanism over Fe-based metal organic frameworks: Combining computational calculation with response surface methodology. *Journal of Hazardous Materials* **2020,** *395*. DOI: 10.1016/j.jhazmat.2020.122686.
41. Mohd Azmi, L. H.; Williams, D. R.; Ladewig, B. P., Polymer-assisted modification of metal-organic framework MIL-96 (Al): influence of HPAM concentration on particle size, crystal morphology and removal of harmful environmental pollutant PFOA. *Chemosphere* **2021,** *262*. DOI: 10.1016/j.chemosphere.2020.128072.
42. Zadehnazari, A.; Khosropour, A.; Zarei, A.; Khazdooz, L.; Amirjalayer, S.; Auras, F.; Abbaspourrad, A., Viologen-Derived Covalent Organic Frameworks: Advancing PFAS Removal Technology with High Adsorption Capacity. *Small* **2024,** *20* (46). DOI: 10.1002/smll.202405176.
43. Jrad, A.; Das, G.; Alkhatib, N.; Prakasam, T.; Benyettou, F.; Varghese, S.; Gándara, F.; Olson, M.; Kirmizialtin, S.; Trabolsi, A., Cationic covalent organic framework for the fluorescent sensing and cooperative adsorption of perfluorooctanoic acid. *Nat Commun* **2024,** *15* (1), 10490 DOI: 10.1038/s41467-024-53945-4.

44. Yu, Q.; Zhang, R.; Deng, S.; Huang, J.; Yu, G., Sorption of perfluorooctane sulfonate and perfluorooctanoate on activated carbons and resin: Kinetic and isotherm study. *Water Research* **2009,** *43* (4), 1150-1158. DOI: 10.1016/j.watres.2008.12.001.
45. Deng, S.; Nie, Y.; Du, Z.; Huang, Q.; Meng, P.; Wang, B.; Huang, J.; Yu, G., Enhanced adsorption of perfluorooctane sulfonate and perfluorooctanoate by bamboo-derived granular activated carbon. *Journal of Hazardous Materials* **2015,** *282,* 150-157. DOI: 10.1016/j.jhazmat.2014.03.045.
46. Liu, X.; Zhu, C.; Yin, J.; Li, J.; Zhang, Z.; Li, J.; Shui, F.; You, Z.; Shi, Z.; Li, B.; Bu, X. H.; Nafady, A.; Ma, S., Installation of synergistic binding sites onto porous organic polymers for efficient removal of perfluorooctanoic acid. *Nature Communications* **2022,** *13* (1). DOI: 10.1038/s41467-022-29816-1.
47. Xiao, L.; Ling, Y.; Alsbaiee, A.; Li, C.; Helbling, D. E.; Dichtel, W. R., β-Cyclodextrin Polymer Network Sequesters Perfluorooctanoic Acid at Environmentally Relevant Concentrations. *Journal of the American Chemical Society* **2017,** *139* (23), 7689-7692. DOI: 10.1021/jacs.7b02381.
48. Wang, R.; Lin, Z. W.; Klemes, M. J.; Ateia, M.; Trang, B.; Wang, J.; Ching, C.; Helbling, D. E.; Dichtel, W. R., A Tunable Porous β-Cyclodextrin Polymer Platform to Understand and Improve Anionic PFAS Removal. *ACS Central Science* **2022,** *8* (5), 663-669. DOI: 10.1021/acscentsci.2c00478.
49. Huang, J.; Shi, Y.; Xu, J.; Zheng, J.; Zhu, F.; Liu, X.; Ouyang, G., Hollow Covalent Organic Framework with "Shell-Confined" Environment for the Effective Removal of Anionic Per- and Polyfluoroalkyl Substances. *Advanced Functional Materials* **2022**. DOI: 10.1002/adfm.202203171.
50. Ramos, P.; Singh Kalra, S.; Johnson, N. W.; Khor, C. M.; Borthakur, A.; Cranmer, B.; Dooley, G.; Mohanty, S. K.; Jassby, D.; Blotevogel, J.; Mahendra, S., Enhanced removal of per- and polyfluoroalkyl substances in complex matrices by polyDADMAC-coated regenerable granular activated carbon. *Environmental Pollution* **2022,** *294*. DOI: 10.1016/j.envpol.2021.118603.
51. Wang, B.; Lee, L. S.; Wei, C.; Fu, H.; Zheng, S.; Xu, Z.; Zhu, D., Covalent triazine-based framework: A promising adsorbent for removal of perfluoroalkyl acids from aqueous solution. *Environmental Pollution* **2016,** *216,* 884-892. DOI: 10.1016/j.envpol.2016.06.062.
52. Huang, P. J.; Hwangbo, M.; Chen, Z.; Liu, Y.; Kameoka, J.; Chu, K. H., Reusable Functionalized Hydrogel Sorbents for Removing Long- and Short-Chain Perfluoroalkyl Acids (PFAAs) and GenX from Aqueous Solution. *ACS Omega* **2018,** *3* (12), 17447-17455. DOI: 10.1021/acsomega.8b02279.
53. Hossain, S.; Stuart, T. D.; Ramarao, B. V.; VanLeuven, C. C.; Wriedt, M.; Kiemle, D.; Satchwell, M.; Kumar, D., Investigation into Cationic Surfactants and Polyelectrolyte-Coated β-Zeolites for Rapid and High-Capacity Adsorption of Short- and Long-Chain PFAS. *Industrial and Engineering Chemistry Research* **2023,** *62* (21), 8373-8384. DOI: 10.1021/acs.iecr.3c00468.
54. Lauwers, A.; Vercammen, J.; De Vos, D., Adsorption of PFAS by All-Silica Zeolite β: Insights into the Effect of the Water Matrix, Regeneration of the Material, and Continuous PFAS Adsorption. *ACS Applied Materials and Interfaces* **2023**. DOI: 10.1021/acsami.3c12321.
